# *C16ORF70/*MYTHO promotes healthy aging in *C.elegans* and prevents cellular senescence in mammals

**DOI:** 10.1172/JCI165814

**Published:** 2024-06-13

**Authors:** Anais Franco-Romero, Valeria Morbidoni, Giulia Milan, Roberta Sartori, Jesper Wulff, Vanina Romanello, Andrea Armani, Leonardo Salviati, Maria Conte, Stefano Salvioli, Claudio Franceschi, Viviana Buonomo, Casey O. Swoboda, Paolo Grumati, Luca Pannone, Simone Martinelli, Harold B.J. Jefferies, Ivan Dikic, Jennifer van der Laan, Filipe Cabreiro, Douglas P. Millay, Sharon A. Tooze, Eva Trevisson, Marco Sandri

**Affiliations:** 1 Department of Biomedical Sciences, University of Padova, Padova, Italy.; 2 Veneto Institute of Molecular Medicine, Padova, Italy.; 3 Clinical Genetics Unit, Department of Women’s and Children’s Health, University of Padova, Padova, Italy.; 4 Pediatric Research Institute (IRP) - Fondazione Città della Speranza, Padova, Italy.; 5 Department of Cardiac Surgery, University Hospital Basel and Department of Biomedicine, University of Basel, Basel, Switzerland.; 6 Institute of Biochemistry II, Goethe University Frankfurt - Medical Faculty, University Hospital, Frankfurt am Main, Germany.; 7 Department of Medical and Surgical Science (DIMEC), University of Bologna, Bologna, Italy.; 8 IRCCS Azienda Ospedaliero-Universitaria di Bologna, Bologna, Italy.; 9 Institute of Information Technologies, Mathematics and Mechanics, Lobachevsky State University, Nizhny Novgorod, Russia.; 10 Telethon Institute of Genetics and Medicine (TIGEM), Pozzuoli, Italy.; 11 Division of Molecular Cardiovascular Biology, Cincinnati Children’s Hospital Medical Center, Cincinnati, Ohio, USA.; 12 Division of Biomedical Informatics, Cincinnati Children’s Hospital Medical Center, Cincinnati, Ohio, USA.; 13 Department of Clinical Medicine and Surgery, University of Naples Federico II, Naples, Italy.; 14 Department of Oncology and Molecular Medicine, Istituto Superiore di Sanità, Rome, Italy.; 15 The Francis Crick Institute, Molecular Cell Biology of Autophagy, London, United Kingdom.; 16 Buchmann Institute for Molecular Life Sciences, Goethe University Frankfurt - Riedberg Campus, Frankfurt am Main, Germany.; 17 CECAD Research Cluster, University of Cologne, Cologne, Germany.; 18 Institute of Clinical Sciences, Imperial College London, Hammersmith Hospital Campus, London, UK.; 19 Department of Pediatrics, University of Cincinnati College of Medicine, Cincinnati, Ohio, USA.; 20 Myology Center, University of Padova, Padova, Italy.; 21 Department of Medicine, McGill University, Montreal, Canada.

**Keywords:** Aging, Cell biology, Autophagy, Cellular senescence, Skeletal muscle

## Abstract

The identification of genes that confer either extension of life span or accelerate age-related decline was a step forward in understanding the mechanisms of aging and revealed that it is partially controlled by genetics and transcriptional programs. Here, we discovered that the human DNA sequence *C16ORF70* encodes a protein, named MYTHO (macroautophagy and youth optimizer), which controls life span and health span. MYTHO protein is conserved from *Caenorhabditis* *elegans* to humans and its mRNA was upregulated in aged mice and elderly people. Deletion of the orthologous *myt-1* gene in *C*. *elegans* dramatically shortened life span and decreased animal survival upon exposure to oxidative stress. Mechanistically, MYTHO is required for autophagy likely because it acts as a scaffold that binds WIPI2 and BCAS3 to recruit and assemble the conjugation system at the phagophore, the nascent autophagosome. We conclude that *MYTHO* is a transcriptionally regulated initiator of autophagy that is central in promoting stress resistance and healthy aging.

## Abstract 2

*C16ORF70*, encoding the MYTHO protein, controls autophagy and is central in promoting stress resistance to ensure healthy aging.

## Graphical abstract

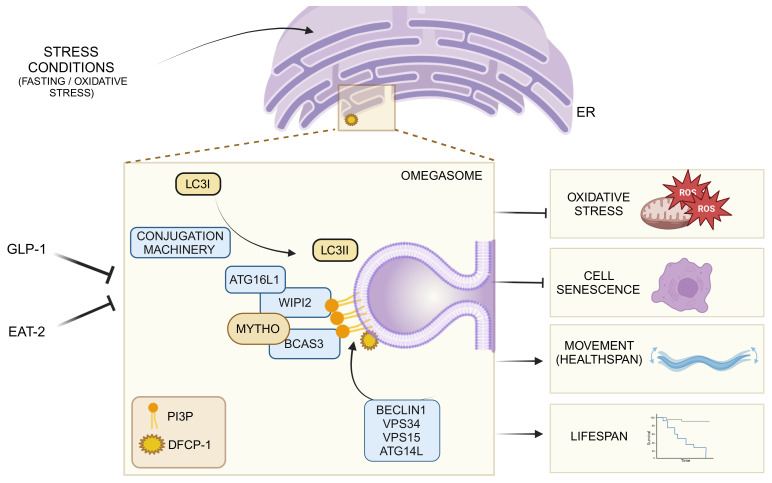

## Introduction

In nature, organisms are continuously exposed to environmental stresses that challenge their survival. The species that quickly and efficiently adapt to hostile conditions are positively selected. This response is regulated by evolutionary conserved signaling pathways that promote transcriptional changes, which in turn limit tissue damage and foster repair and stress resistance (1). *Caenorhabditis* *elegans* is an invaluable animal model commonly employed to decipher the molecular and genetic bases for longevity and aging. Two of the first identified long-lived *C*. *elegans* mutants carried mutations in genes involved in the insulin/IGF-1 signaling pathway: *daf-2* and *age-1* (2, 3). Mutation in *daf-2*, which is the *C*. *elegans* homolog of the mammalian insulin/IGF-1 receptor, was demonstrated to more than double life span. This expanded longevity requires the activity of the FoxO transcription factor *daf-16* (4, 5). In this scenario, FoxOs regulate several stress response pathways and consequently are critical to restrain aging and promote longevity (6, 7). Another longevity player of the insulin/IGF-1 signaling pathway is *age-1*. It encodes the catalytic subunit of class I phosphatidylinositol 3-kinase, which catalyzes conversion of phosphatidylinositol 4,5-bisphosphate (PIP_2_) into phosphatidylinositol 3,4,5-trisphosphate (PIP_3_) (8).

A general cause of cellular senescence and organism aging is the progressive accumulation of dysfunctional organelles and cellular damage. Impairment of proteostasis alters the protein quality control systems, leading to the accumulation of aberrant and dysfunctional macromolecules and is considered among the primary hallmarks of aging (9). All cells take advantage of an array of mechanisms to preserve the stability and functionality of their proteins or to remove them when they are irreversibly damaged. One of the most important cellular housekeeping and prosurvival pathways is macroautophagy, hereafter named autophagy, whose main action is to remove damaged proteins/organelles and generate molecules that sustain cellular core metabolism. Autophagy contributes to prolonging life span and health span in mammals (10–13) by removing damaged organelles, allowing for the rejuvenation of cellular components. Consistently, inhibition of autophagy results in disease onset and premature senescence in mammals (12). The process of autophagosome formation is catalyzed by a complex machinery that contains protein and lipid kinases, membrane-binding and lipid transfer proteins, and ubiquitin-like conjugation systems (14). How these components are assembled and act in an ordered manner to generate autophagosomes is still unclear (15, 16). Interestingly, the environmental clues that promote autophagy activation are also potent stimulators of FoxO in yeast and mammalian cells (17).

However, how context-dependent molecular networks contribute to limit tissue damage, promote repair and, ultimately, longevity remains unclear. This is partially a consequence of the fact that many potential protein-encoding genes in the human genome are still uncharacterized. Indeed, recent meta-analysis studies show that of our 20,000 protein-encoding genes, more than 5,000 are still uncharacterized (18). Here, we report on the identification of a gene that is conserved from *C*. *elegans* to humans and plays a critical role in promoting autophagy, stress resistance, and healthy aging.

## Results

### MYTHO is a highly conserved gene that is induced in aging.

**Figure 1:**
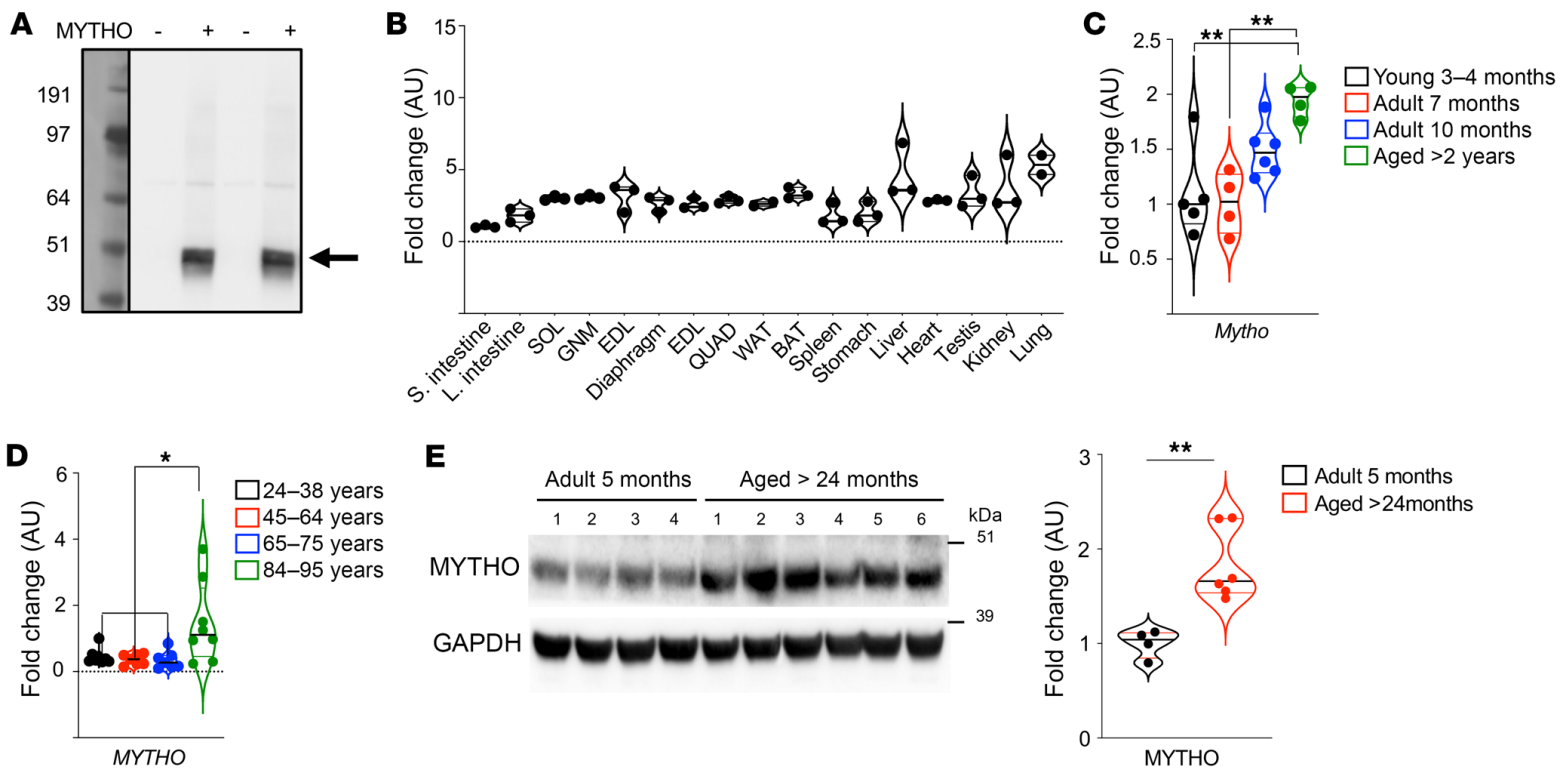
*C16orf70* encodes a protein named MYTHO that is expressed in different tissues and upregulated in aging. (**A**) PBI-eGFP/3xFlag-MYTHO vector or PBI-eGFP/3xFlag–empty vector was transfected into HEK293A cells. 3×FLAG-MYTHO expression was observed using an anti-FLAG antibody. The blot shown in the image represents the results of 4 independent transfections. (**B**) Quantitative RT-PCR of *Mytho* in different organs and muscles from 5-month-old male mice. mRNA expression was calculated by the ΔCt method and expressed as fold increase from the tissue where *Mytho* is less expressed (small intestine). SOL, soleus muscle; GNM, gastrocnemius muscle; EDL, extensor digitorum longus muscle; TA, tibialis anterior muscle; QUAD, quadriceps muscle; WAT, white adipose tissue; BAT, brown adipose tissue. *n* = 3 for all tissues, *n* = 2 only for WAT and lung. (**C**) Quantitative real-time PCR of *Mytho* from mice of different ages (3–4 months, *n* = 5; 7 months, *n* = 4; 10 months, *n* = 6; and >2 years old, *n* = 4). Expression was normalized to that of *Gapdh* and is expressed as fold increase (1-way ANOVA with Tukey’s multiple-comparison test). (**D**) Quantitative real-time PCR of *MYTHO* in muscle biopsies from patients of different ages: 24–38 years old (*n* = 8), 45–64 years old (*n* = 7), 67–75 years old (*n* = 7), and 84–95 years old who underwent surgery for hip replacement (*n* = 8). All data were normalized to *GAPDH* and are expressed as fold increase from the 24- to 38-year-old control group (1-way ANOVA with Tukey’s multiple-comparison test). (**E**) Immunoblot of homogenates from GNM muscle from 5-month-old mice (*n* = 4) and >24-month-old (*n* = 6) mice. Anti-C16orf70 antibody was used to detect MYTHO endogenous protein. Normalization was performed using GAPDH and data are expressed as fold increase (2-tailed Student’s *t* test). All bars indicate SEM. *P < 0.05; **P < 0.01.

**Figure 2:**
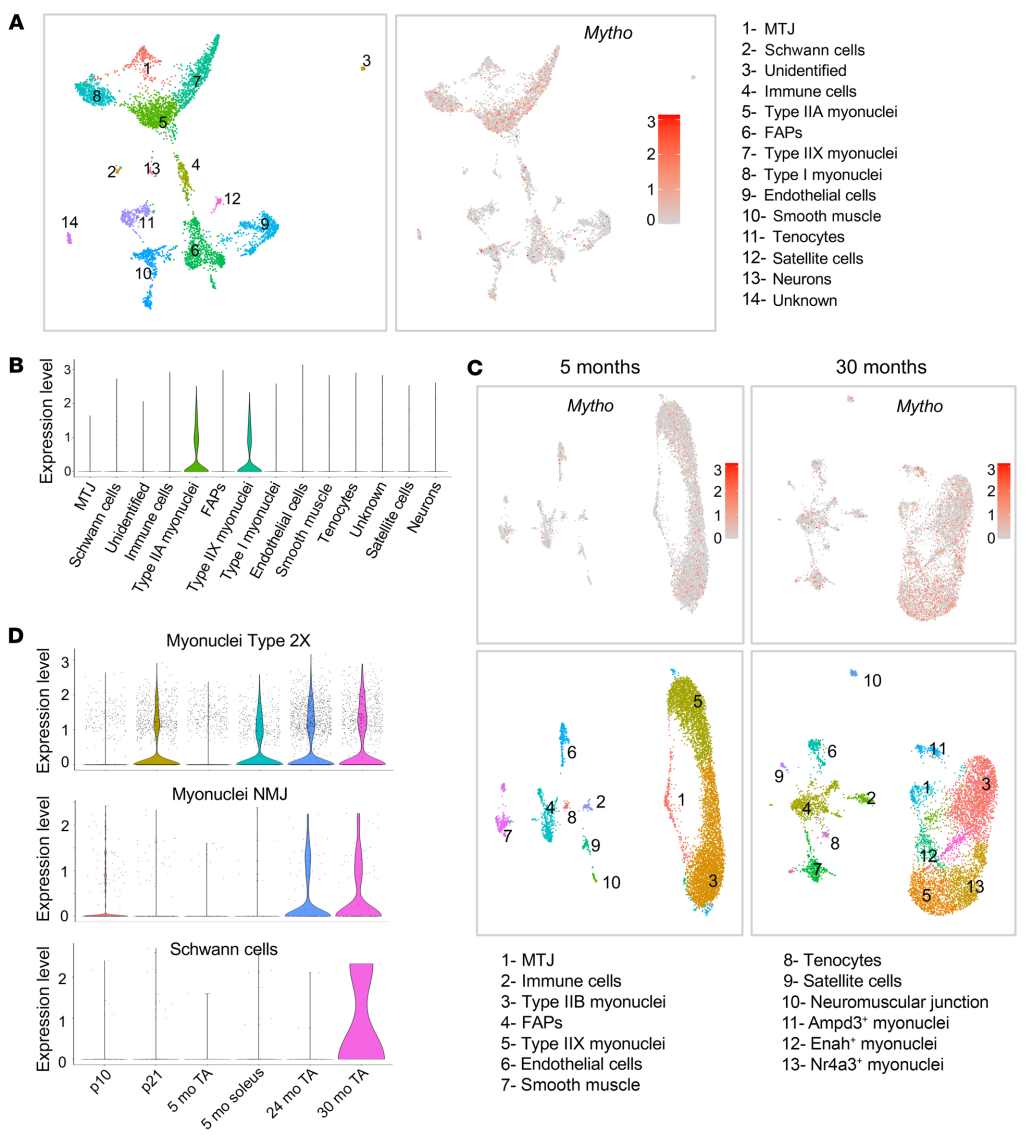
*Mytho* expression increases in muscle during aging. snRNA-seq data re-elaborated from Myoatlas (https://research.cchmc.org/myoatlas/). (**A**) UMAP showing snRNA-seq expression of *Mytho* in different cell population from 5-month-old SOL muscle. MTJ, myotendinous junction; FAPs, fibro-adipogenic progenitors. (**B**) Violin plots with the quantification of *Mytho* expression in the different cell types. (**C**) UMAP of the different population of snRNA-seq showing *Mytho* expression at 5 months and 30 months. (**D**) Violin plots showing nuclear transcriptomic profiles of *Mytho* gene in myonuclei type 2X, myonuclei NMJ, and Schwann cells of animals at different ages (p10, postnatal day 10; p21, postnatal day 21; 5 mo, 5 months; 24 mo, 24 months; 30 mo, 30 months; TA, tibialis anterior).

To identify uncharacterized factors that control aging and proteostasis, we screened our published transcriptomic profiles (19) for DNA sequences with unknown function that were upregulated in conditions of enhanced protein breakdown (e.g., during fasting), but not when proteolysis was blocked by inhibition of FoxOs (19). Then, DNA sequences were further screened using bioinformatics tools for the presence of an open reading frame (ORF) and conservation across species. To further reduce the number of candidates, we searched for either the presence of autophagy-related LC3 interaction regions/GABARAP interaction motifs (LIRs/GIMs) in the coding region or for published evidence of an interaction with autophagy proteins. This screening identified one candidate, *D230025D16Rik* (mouse)/*C16ORF70* (human), which we named *MYTHO* (macroautophagy and youth optimizer). This gene appeared highly conserved across species from *C*. *elegans* to human (37% amino acid sequence homology) and showed 95% amino acid sequence homology between human and mouse (Supplemental Figure 1; supplemental material available online with this article; https://doi.org/10.1172/JCI165814DS1). To determine whether the *MYTHO* gene encoded a functional protein, we cloned the DNA sequence in an expression vector and transfected it into HEK293T cells. A FLAG-tagged 48 kDa protein was expressed in transfected cells that agrees with the predicted molecular weight of a 422–amino acid protein (Figure 1A). Quantitative RT-PCR revealed that *Mytho* is expressed in several tissues such as lung, liver, and heart, and in different skeletal muscles, including mitochondria-poor (tibialis anterior and gastrocnemius) and mitochondria-rich muscles (soleus) (Figure 1B). Since we screened for genes that may be important for longevity, we checked *Mytho* transcript expression during aging and found an upregulation in muscles of very old mice (Figure 1C). We then checked the expression of *MYTHO* in human vastus lateralis muscle biopsies from patients of different ages. Similar to mice, we found that the oldest group (84–96 years) displayed a significantly higher *MYTHO* expression level (Figure 1D). Finally, we monitored MYTHO protein in muscle of 25-month-old mice and confirmed an increased expression (Figure 1E). To establish which nuclei of muscle tissue express *Mytho*, we consulted a published data set of single-nucleus RNA sequencing (snRNA-seq) from tibialis anterior and soleus. Importantly, this data set also contained information on nuclear transcriptomic profiles of animals at different ages such as postnatal days 10 and 21, 5 months, 24 months, and 30 months (20). When we tested which types of nuclei among the several clusters express *Mytho*, myonuclei showed the highest enrichment. In soleus, the highest expression was found in myonuclei of type 2A and 2X fibers (Figure 2, A and B), while in tibialis anterior the transcript was less abundant in adulthood but showed an age-related pattern (Figure 2C). Indeed, *Mytho* was transiently expressed in type 2X fibers at postnatal day 21 and was reinduced at 24 and 30 months of age (Figure 2D). Interestingly, myonuclei of the neuromuscular junction (NMJ) also showed a transiently higher expression at postnatal day 10 and a further induction in aged animals (Figure 2D). In addition, the neuronal part of the NMJ, constituted by the Schwann cells, also showed the upregulation of *Mytho* at 30 months of age (Figure 2D). These findings confirm that the *Mytho* gene is regulated during neonatal life and in aging mainly in myonuclei and nuclei belonging to the presynaptic and postsynaptic site of the NMJ and to the neuronal axons.

Because this gene is conserved across species and because *C*. *elegans* is an established animal model for aging studies, we checked whether the *C*. *elegans* homologous gene *T01G9.2*, hereafter named *myt-1*, showed a similar pattern of tissue and temporal expression. Therefore, we generated a transgenic *C*. *elegans* line that expressed the *gfp* coding sequence from the *myt-1* endogenous promoter (see Methods). Confocal microscopy analyses revealed that *myt-1* was mostly expressed in body wall muscles, neurons, and intestine early during development (Supplemental Figure 2A), while it was mainly expressed in muscles and neurons in adulthood (Supplemental Figure 2, B and C). Next, we tested *myt-1* expression in 11-day-old *C*. *elegans* (counting day 0 at L4 stage) and identified an increased expression in neurons and muscles compared with younger (3-day-old) animals, confirming an age-related regulation also in *C*. *elegans* (Supplemental Figure 2D). Finally, because the initial screening was based on genes induced when nutrients are reduced, we tested whether this expression was affected by 24 hours of starvation. Consistently, GFP signal was dramatically increased by fasting, especially in body wall muscles (Supplemental Figure 2, E and F). Thus, *C*. *elegans* replicates the mammalian tissue-pattern and age-dependent expression of *MYTHO*.

### Inhibition of MYTHO induces cellular senescence and reduces life span in C. elegans.

**Figure 3:**
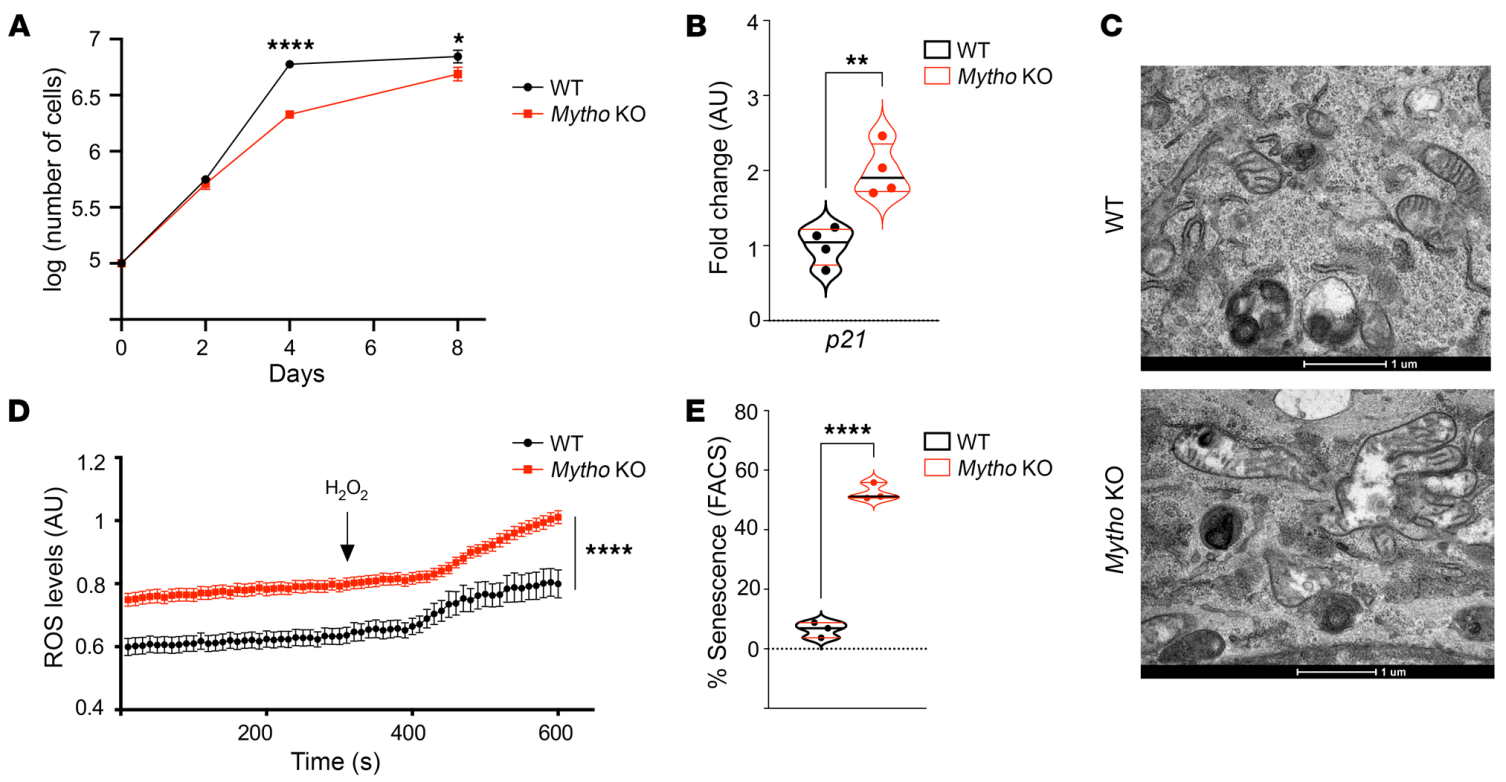
*Mytho* depletion induces cellular senescence. (**A**) The graph shows the logarithm (log) of total number of cells measured at 2 days, 4 days, and 8 days after seeding. Cellular confluence was reached at 4 and 8 days in control and *Mytho*-deficient cells, respectively (*N* = 3) (2-tailed Student’s *t* test). (**B**) Quantitative real-time PCR of *p21* from WT and *Mytho*-KO C2C12 cells normalized to *Gapdh* and expressed as fold increase (*N* = 3) (2-tailed Student’s *t* test). (**C**) Representative electron microscopy images of the cytoplasm of WT (top) and *Mytho*-KO (bottom) C2C12 cells. Abnormal swollen mitochondria are often found in KO cells. Scale bars: 1 μm. (**D**) Mt-roGFP fluorescence was measured in single cells (*n* = 30/condition; *N* = 2). Arrow indicates the addition of H_2_O_2_. (**E**) Percentage senescence in cells measured by FACS after WT and *Mytho*-KO C2C12 cells were incubated with CellEvent Green Senescence Probe (Thermo Fisher Scientific) (*n* = 3) (multiple unpaired *t* test). *N* = number of independent experiments; *n* = total number of individuals. All bars indicate SEM. *P < 0.05, **P < 0.01, ****P < 0.0001.

**Figure 4:**
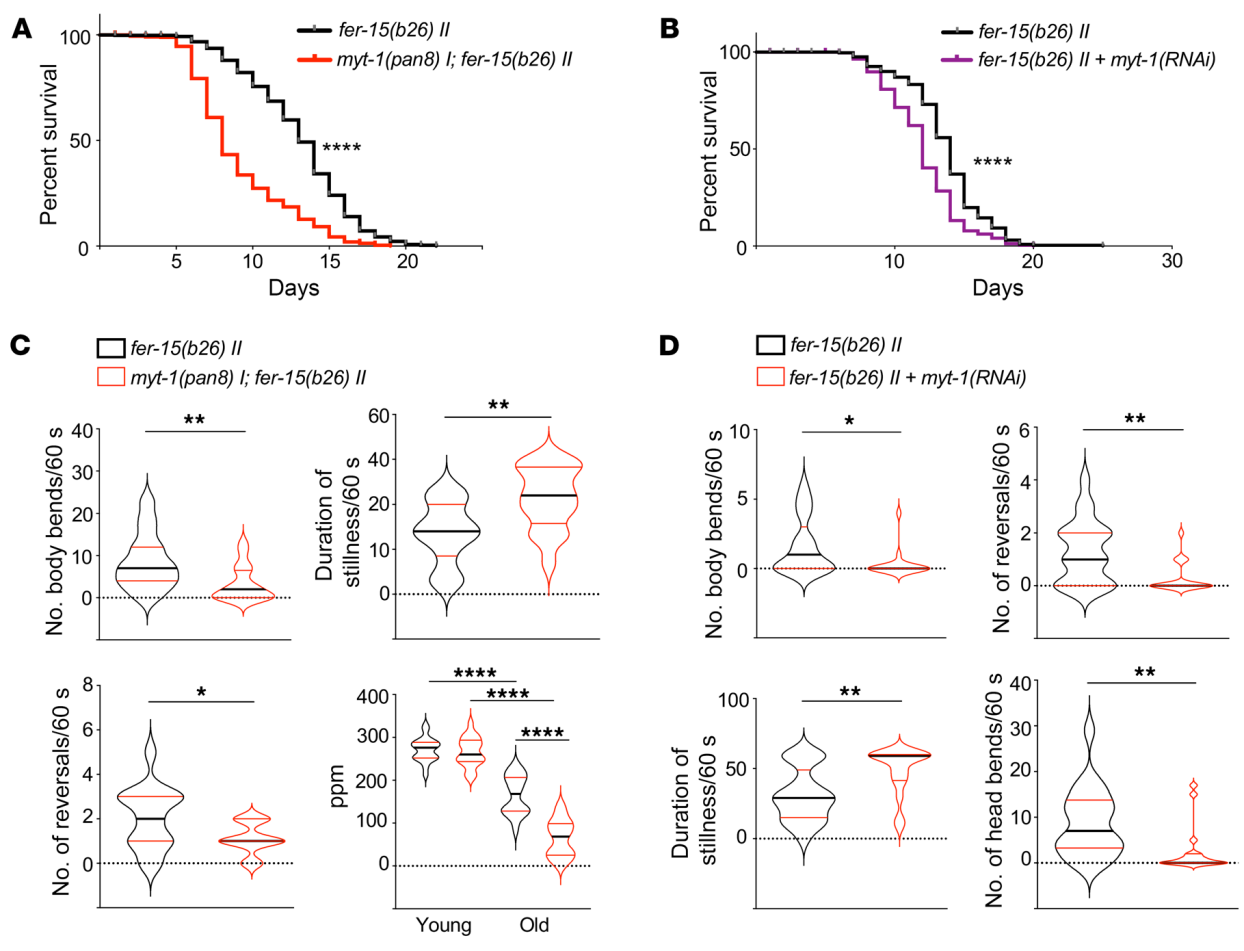
*Mytho* depletion reduces *C*. *elegans* life span and health span. (**A**) Survival curves of *fer-15(b26) II* and *myt-1(pan8) I; fer-15(b26) II* worms (*n* = 521, *N* = 5). (**B**) Survival curves of *fer-15(b26) II* worms fed with either bacteria transformed with pL4440 empty vector (*n* = 252) or with pL4440 containing the *myt-1* coding sequence [*myt-1(RNAi)*, *n* = 253] following a maternal RNAi protocol (see Methods) (*N* = 3). (**C**) Total number of body bends, reversals, and duration of stillness periods calculated for 5-day-old *fer-15(b26) II* (*n* = 15) and *myt-1(pan8) I; fer-15(b26) II* (*n* = 17) animals in spontaneous locomotion (*N* = 3). Pumping rate (pumps/minute) was assessed on day 1 (YOUNG, *n* = 20; *n* = 20) and day 5 (OLD, *n* = 19; *n* = 18) in *fer-15(b26) II* and *myt-1(pan8) I; fer-15(b26) II* animals (*N* = 2). (**D**) Spontaneous locomotion analysis of body and head bends, reversals, and duration of stillness periods in 11-day-old *fer-15(b26) II* animals fed with *myt-1(RNAi)* (*n* = 17) or control bacteria (*n* = 21) (*N* = 2). Log-rank (Mantel-Cox) test was used to compare longevity curves in **A** and **B** (see Supplemental Figure 6A for life span experimental details and statistics). Bars in **C** and **D** indicate SEM. *P < 0.05; **P < 0.01; ****P < 0.0001 (2-tailed Student’s *t* test). *N* = number of independent experiments; *n* = total number of individuals.

The finding that *MYTHO* was upregulated in very old people and mice suggested a potential role of this gene in counteracting aging. Therefore, we tested whether inhibition of *Mytho* would induce a premature cellular senescence. To address this point, we generated a muscle cell line in which *Mytho* was deleted by CRISPR/Cas9 technology (Supplemental Figure 3, A–F) and investigated cellular senescence. Premature cellular aging is typically characterized by low replicative rates due to upregulation of cyclin inhibitors such as p21 and p16, accumulation of dysfunctional mitochondria, proteostasis dysfunction, and DNA damage, among other cellular events. In the absence of *Mytho*, cell proliferation was slowed down, *p21* expression was increased, mitochondria showed abnormal morphology and function, which was revealed by ROS production, and the senescence-associated β-galactosidase activity, a marker of lysosomal impairment, was increased (Figure 3, A–E). To further characterize the functional relevance of this gene in physiology as well in life span of a multicellular organism, we moved back to *C*. *elegans*. By CRISPR/Cas9 technology, we generated 3 independent *C*. *elegans* mutant lines in which the *myt-1* gene was disrupted, i.e., *myt-1(pan8) I*, *myt-1(pan9) I*, and *myt-1(pan10) I* (Supplemental Figure 4, A–E). Phenotypic analyses were performed on *myt-1(pan8) I* and *myt-1(pan9) I* strains. Knockout (KO) animals displayed a problem of egg delivery, as revealed by a higher prevalence of egg retention with internal hatching, called a “bagging” phenotype, compared with controls (Supplemental Figure 5A), possibly due to the physiological role of *myt-1* in vulval muscles. Notably, *myt-1* animals that did not exhibit a bagging phenotype displayed a significantly shortened life span (Supplemental Figure 5B and Supplemental Figure 6A). Next, to better establish *myt-1* involvement in longevity independently of the bagging phenotype, we crossed the *myt-1(pan8)* *I* strain with *fer-15(b26) II* animals. This genetic background confers temperature-dependent sterility. Consistently, the absence of *myt-1* resulted in a dramatic reduction in *C*. *elegans* life span (Figure 4A and Supplemental Figure 6A). Identical results were obtained by crossing a second *myt-1 (pan9)* *I* strain (Supplemental Figure 5C and Supplemental Figure 6A). Reduction of survival was further confirmed by RNAi-mediated knockdown of *myt-1*, which significantly shortened the life span of *C*. *elegans* (Figure 4B and Supplemental Figure 6B). Moreover, when animals were challenged with oxidative stress by paraquat treatment, survival was significantly reduced compared with controls (Supplemental Figure 5D). Because life span does not necessarily match with quality of life, we checked *C*. *elegans*’ movements as a health readout. In fact, body movement is one of the most obvious behavioral abnormalities associated with nematode aging (21). While the worms’ movements were not significantly different in young *myt-1*–KO animals (data not shown), they were dramatically reduced in old worms (Figure 4C). Moreover, pharyngeal pumping was also significantly decreased in *myt-1*–deficient old worms, but not in young animals (Figure 4C). Importantly, a significant reduction in movement in the absence of *myt-1* was also obtained by comparing WT and KO animals at the mean life span, supporting the role of *myt-1* in health span (Supplemental Figure 5E). These findings suggest that muscle maturation/generation was preserved during youth, but that premature aging and decline in muscle function happened in adulthood in *myt-1*–deficient animals. Consistently, similar results were obtained when the *myt-1* gene was knocked down by RNAi and spontaneous or touch-induced worms’ movements were quantified on day 11 (Figure 4D and Supplemental Figure 5F). Next, we tested whether muscle-specific *myt-1* overexpression during aging is beneficial for health span. We analyzed the worms’ movements on day 14 and found a significant increase in body bends and head movements in transgenic animals, as well as a decreased stillness time (Supplemental Figure 5G). Despite the beneficial effect on health span, life span was not improved (Supplemental Figure 6A). Altogether, these findings strongly support the notion that *MYTHO* is required to preserve cellular function during aging.

### MYTHO is recruited to autophagosomes and is required for optimal autophagic flux.

**Figure 5:**
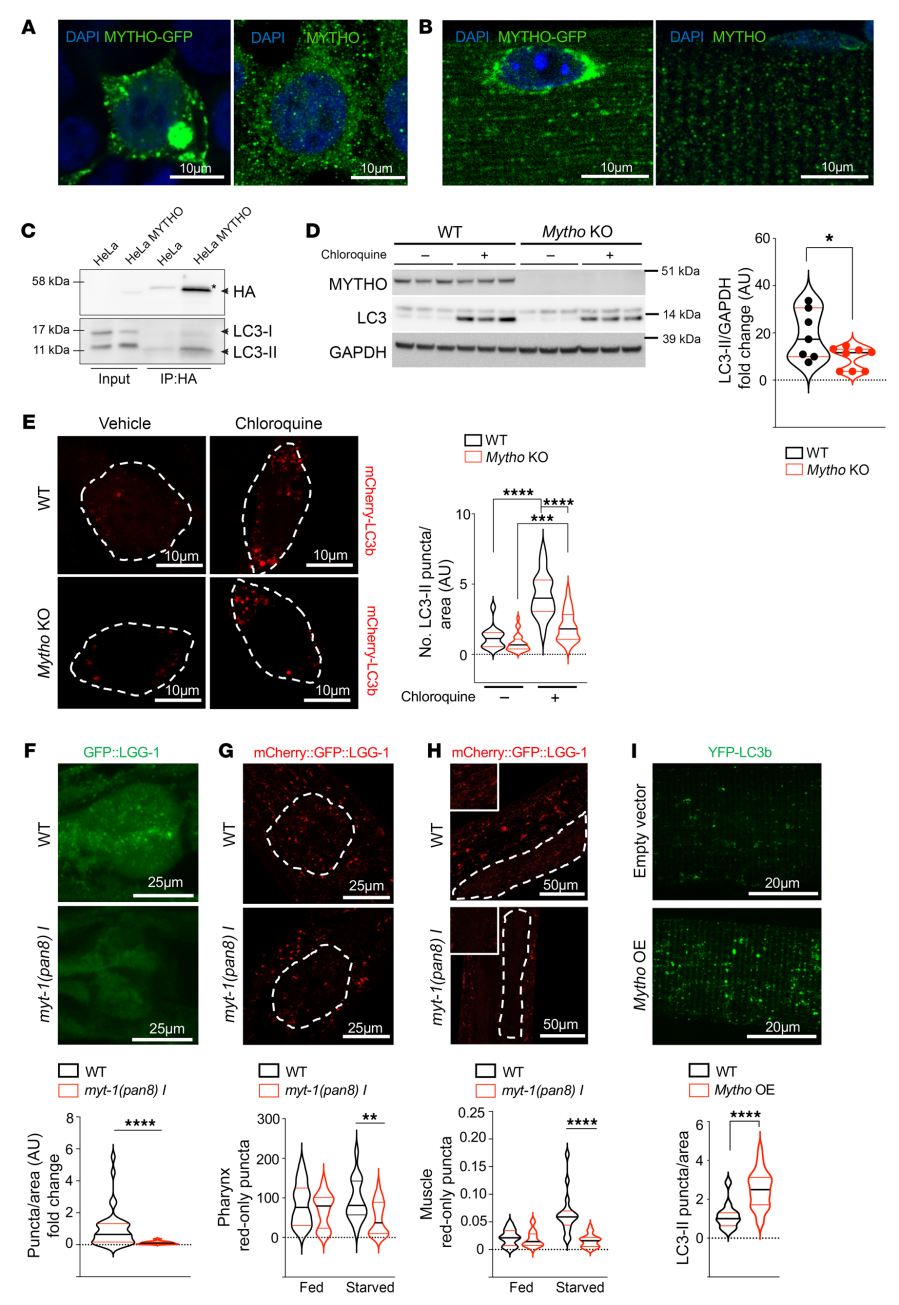
Depletion of *Mytho* reduces autophagic flux in vitro and in vivo. (**A**) Left: Representative images of HEK293 cells transfected with MYTHO-GFP. Right: Representative image of endogenous MYTHO. Scale bars: 10 μm. (**B**) Left: FDB muscles transfected with MYTHO-GFP. Right: Endogenous Mytho in FDB fibers. Scale bars: 10 μm. (**C**) Endogenous HA-tagged Mytho coimmunoprecipitates with LC3B. The asterisk (*) indicates a nonspecific band. (**D**) LC3 lipidation was analyzed by immunoblot in WT and *Mytho*-KO C2C12 cells treated or not with chloroquine. LC3-II band was normalized to GAPDH (*n* = 8) (2-tailed Student’s *t* test). (**E**) Left: Representative fluorescence images of WT and *Mytho*-KO C2C12 cells transfected with Cherry-LC3B and treated with chloroquine or vehicle. Scale bars: 10 μm. Right: Quantification of LC3 puncta/area of the cell in each condition is shown (*n* > 15 cells/condition) (1-way ANOVA with Tukey’s multiple-comparison test). (**F**) Top: Representative fluorescence images of GFP:LGG-1 puncta in the posterior bulb of the pharynx of N2 (WT) and *myt-1(pan8) I* worms. Scale bar: 25 μm. Bottom: Autophagosomal pool quantification in WT (*n* = 26) and *myt-1(pan8)* *I* (*n* = 20) worms (*N* = 3) (2-tailed Student’s *t* test). (**G** and **H**) Top: Representative fluorescence images of mCherry:LGG-1 puncta in the posterior bulb of the pharynx (**G**) and in body wall muscle (**H**) of N2 (WT) and *myt-1(pan8) I* worms. Scale bars: 25 μm (**G**) and 50 μm (**H**). Bottom: Relative quantification of mCherry:LGG-1 puncta in basal condition (Fed) and after 24-hour starvation (Starved) in M9 buffer. WT Fed (*n* = 14), *myt-1(pan8)* *I* Fed (*n* = 22), WT STV 24 h (*n* = 17), *myt-1(pan8) I* STV 24 h (*n* = 27); *N* = 2 (2-tailed Student’s *t* test). (**I**) Top: Representative fluorescence images of single fibers from FDB muscle transfected with YFP-LC3/3xFlagMYTHO or YFP-LC3/Flag–empty vector in basal condition. Scale bars: 20 μm. Bottom: Quantification of LC3 puncta in more than 12 fibers (2-tailed Student’s *t* test). All bars indicate SEM. *P < 0.05; **P < 0.01; ***P < 0.001; ****P < 0.0001. *N* = number of independent experiments; *n* = number of cells/samples.

To identify how *MYTHO* preserves cellular functions during aging, we performed localization studies in vivo and in vitro. Given the high expression level of the gene in muscles of worms and mice, and since genetic manipulation is straightforward in adult mouse muscles (22), in vivo experiments were performed in skeletal muscle. Immunofluorescence analyses on transfected HEK293 cells showed a punctate pattern of staining that was similar to the localization of the endogenous protein (Figure 5A). An identical pattern was also detected when *Mytho* was overexpressed in adult skeletal muscles or when the endogenous protein was revealed (Figure 5B). By CRISPR/Cas12 technology (23), we HA-tagged the endogenous MYTHO protein in cells (Supplemental Figure 7A). Immunofluorescence analyses with an anti-HA antibody showed a minor colocalization of MYTHO with mitochondria and peroxisomes, and almost no colocalization with endoplasmic reticulum (ER) or Golgi in basal conditions (Supplemental Figure 7, B–F). Interestingly, we found that 40% of DFCP1-positive puncta, which under starvation conditions reveal the ER regions enriched with phosphatidylinositol 3-phosphate (PtdIns3P) named omegasomes, colocalized with MYTHO (Supplemental Figure 7G). Because of this finding and since the initial bioinformatic screen suggested several putative LIRs in the protein, we tested whether MYTHO colocalized with LC3B or LAMP2, 2 established markers of autophagosomes and lysosomes, respectively. MYTHO-GFP colocalized with Cherry-LC3B (Supplemental Figure 8A) in vitro, and with LAMP2-Cherry in vivo (Supplemental Figure 8B). Moreover, when we expressed MYTHO-GFP in muscle-specific autophagy-deficient *Atg7*-KO mice, MYTHO localization on lysosomes was completely abrogated, suggesting that it requires autophagy to reach lysosomes (Supplemental Figure 8B). Finally, by pulling down the endogenous tagged MYTHO protein with an anti-HA antibody we confirmed that LC3B-II coimmunoprecipitates (Figure 5C). To further support the critical role of MYTHO in autophagy, we tested the autophagic flux in a *Mytho*-KO C2C12 cell line (Figure 5D) by monitoring the increase in LC3B-II in the presence of chloroquine, a lysosome inhibitor. Indeed, the accumulation of the lipidated LC3B (LC3B-II) protein during lysosomal inhibition was proportional to the number of autophagosomes that are generated and docked to the lysosomes. Western blots for LC3 lipidation and immunofluorescence analyses of LC3-positive puncta revealed that chloroquine treatment did not increase LC3-II lipidation in *Mytho*-KO cells as in control cells, indicating that basal autophagic flux is reduced in the absence of *Mytho* (Figure 5, D and E). Consistently, the number and size of p62-positive puncta were significantly increased in *Mytho*-deficient cells (Supplemental Figure 8C). Finally, when we crossed *myt-1*–KO nematodes with animals expressing a GFP-tagged version of LGG-1 (the worm ortholog of ATG8/GABARAP), we noticed a significant decrease in the number of LGG-1–positive puncta localized at the posterior bulb of the pharynx in KO animals compared with WT, suggesting that *myt-1* is required for autophagy also in *C*. *elegans* (Figure 5F). To further support *myt-1* involvement in autophagic flux regulation in vivo, we crossed *myt-1*–KO worms with nematodes expressing the tandem reporter mCherry:GFP:LGG-1 and quantified red puncta in the pharynx and body wall muscles. Because green fluorescence was blunted by the acidic environment of lysosomes, the red signal revealed the number of autophagosomes fused with lysosomes. Consistent with cell culture data, red puncta were significantly decreased in the pharynx and muscles from *myt-1*–deficient worms upon starvation (Figure 5, G and H), suggesting that autophagic flux is also reduced in *C*. *elegans*. Conversely, overexpression of *Mytho* in adult mouse skeletal muscles was sufficient to increase autophagosome numbers (Figure 5I). These findings suggest that *Mytho* is involved in autophagosome formation both in vitro and in vivo.

### MYTHO interacts with WIPI2, allowing the recruitment of the conjugation system at the phagophore.

**Figure 6:**
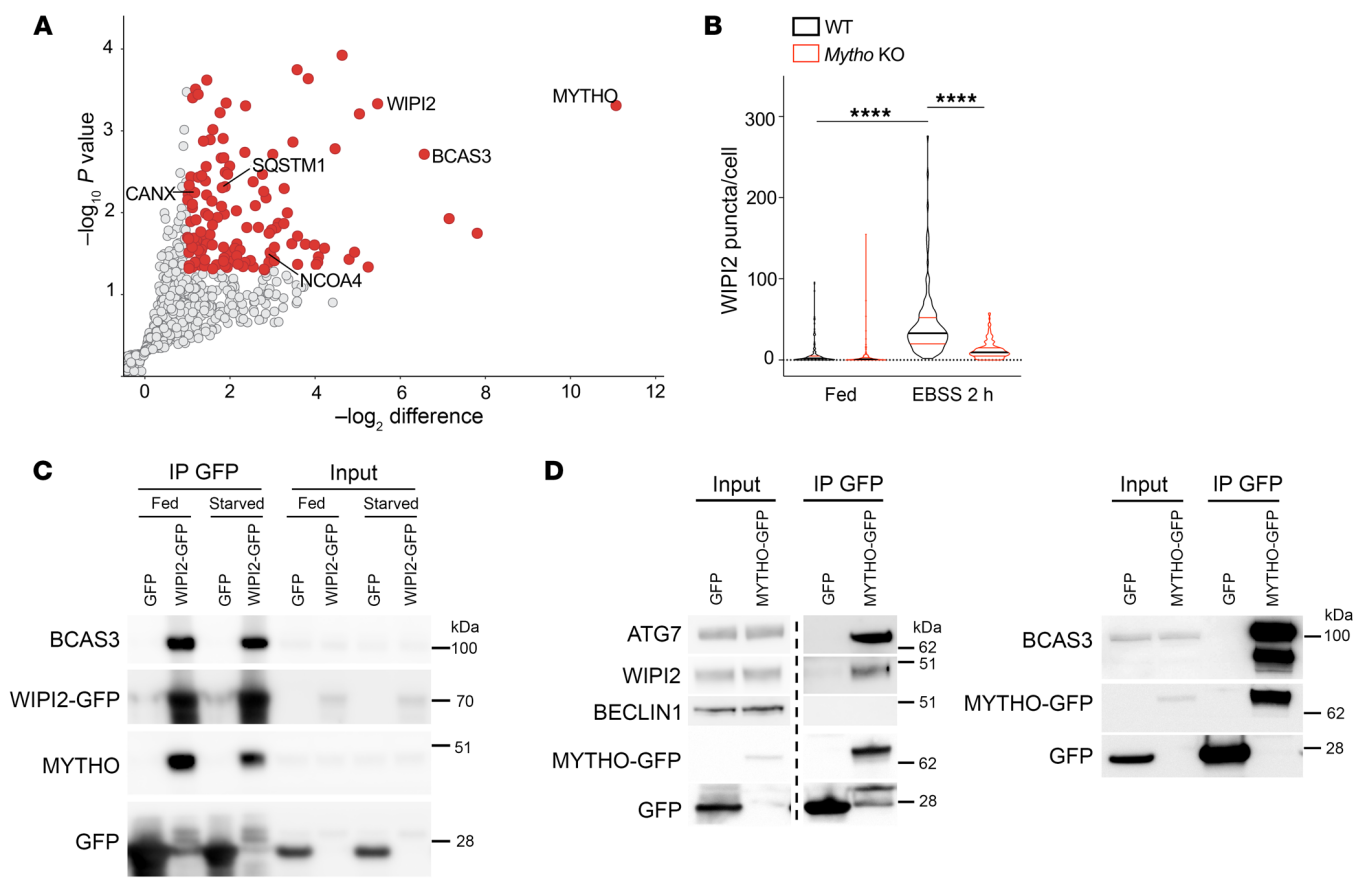
MYTHO interacts with autophagic proteins. (**A**) Mass spectrometry analysis of immunoprecipitated endogenous HA-tagged MYTHO. Significant autophagy-related proteins are shown in the graph. (**B**) Quantification of WIPI2 puncta in Fed and starved (2 hours with Earle’s balanced salt solution [EBSS]) conditions in WT and *Mytho*-KO C2C12 cells in *N* = 3. Fed WT (*n* = 141); Fed *Mytho*-KO (*n* = 144), EBSS 2 h WT (*n* = 139); EBSS 2 h *Mytho*-KO (*n* = 172). Bars indicate SEM. ****P < 0.0001 (1-way ANOVA with Tukey’s multiple-comparison test). (**C**) HEK293 cells transfected with WIPI2-GFP or GFP were immunoprecipitated with GFP-TRAP. The quantification of *N* = 3 (normalized by input) is shown in Supplemental Figure 9E. (**D**) HEK293 cells transfected with MYTHO-GFP or GFP were immunoprecipitated. Endogenous WIPI2, ATG7, and BCAS3 were immunoblotted. In the blot on the left, lanes were run on the same gel but were noncontiguous. The quantification of *N* = 3 (normalized by input) is represented in Supplemental Figure 9F. *N* = number of independent experiments; *n* = number of cells/samples.

To identify how *Mytho* controls autophagy, we pulled down MYTHO and performed proteomic analyses to establish its interactome. Among the different interactors, WIPI2, which plays a critical role in autophagy, as well as different autophagy receptors (e.g., p62/SQSTM1, NCOA) were identified (Figure 6A). Interestingly, another PtdIns3P-binding protein, BCAS3, which has been described to be involved in autophagosome formation (24), was enriched in MYTHO pulldown experiments. We found that WIPI2 puncta were dramatically reduced in starved *Mytho*-deficient cells (Figure 6B and Supplemental Figure 9A) even though total WIPI2 protein was not affected (Supplemental Figure 9B). Accordingly, the recruitment and colocalization of ATG16L1, another WIPI2 partner, with WIPI2 was abolished (Supplemental Figure 9C). Interestingly, BCAS3 puncta were also abolished in the absence of MYTHO during starvation (Supplemental Figure 9D). Furthermore, endogenous MYTHO could be coimmunoprecipitated with GFP-WIPI2 together with BCAS3 (Figure 6C and Supplemental Figure 9E). Moreover, immunoprecipitation of MYTHO-GFP showed an interaction with endogenous WIPI2, BCAS3, and ATG7, the E1 enzyme of the conjugation system, but not BECN1, the protein involved in PtdIns3P generation (Figure 6D and Supplemental Figure 9F). Immunofluorescence analyses with an anti-HA antibody for the endogenous HA-tagged MYTHO protein highlighted the colocalization with WIPI2 and ATG16L1 (Supplemental Figure 10A).

**Figure 7:**
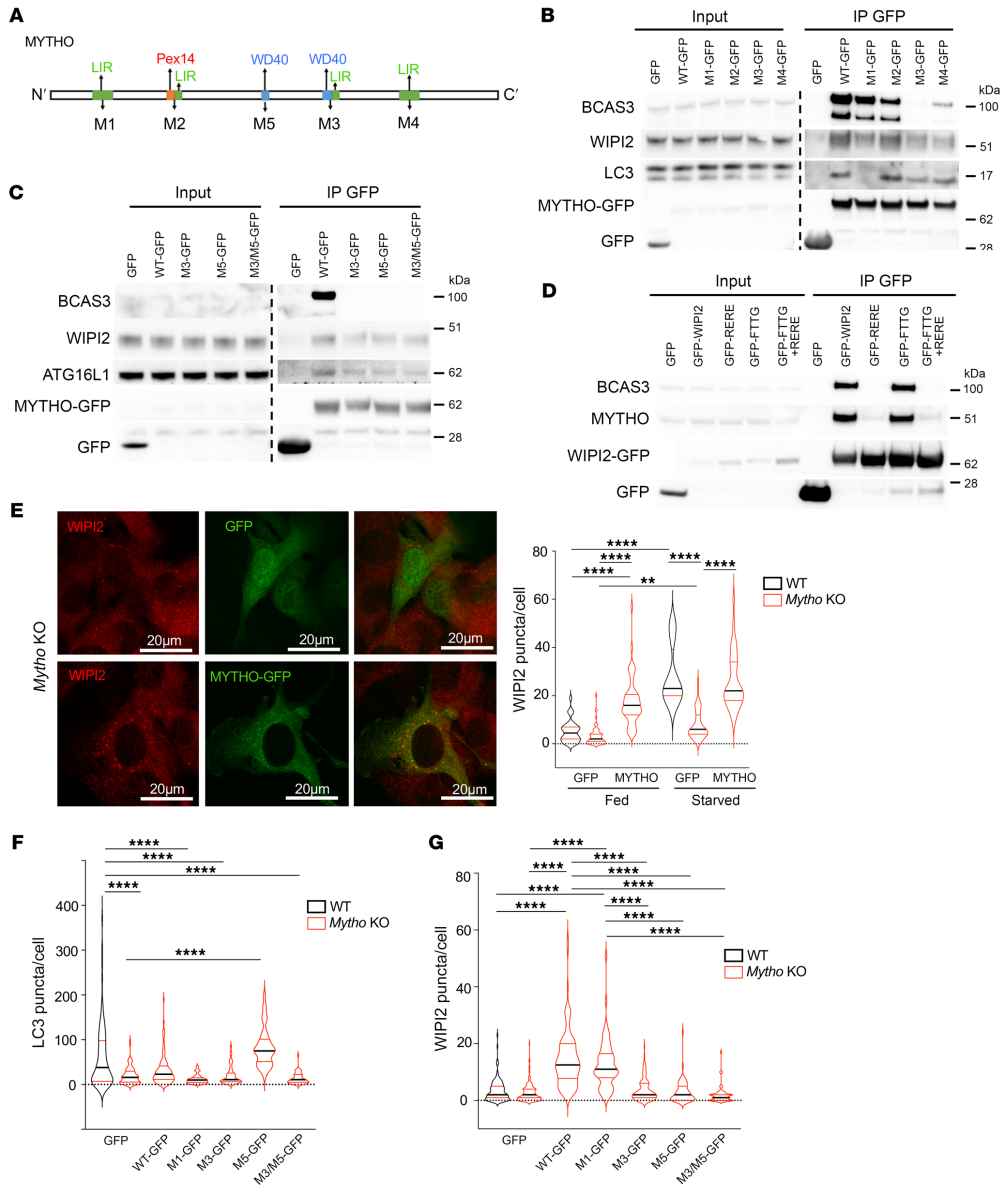
MYTHO is required for WIPI2 and BCAS3 localization on autophagosomes. (**A**) Representative scheme showing predicted LC3 interaction motifs and WD40 domains: Y91A/V94A (M1); F131A/L134A (M2); Y288A/L291A (M3); W351A/I354A (M4); 208delTGPSGLRLRL (M5) or Y288A/L291A + 208delTGPSGLRLRL (M3/M5). (**B** and **C**) HEK293 cells transfected with GFP, MYTHO-GFP, or MYTHO-GFP mutants were lysed and immunoprecipitated with GFP-TRAP, and blotted with indicated markers. All samples were run on the same gel. Quantification of LC3, WIPI2, and BCAS3 enrichment (normalized to input) is shown in Supplemental Figure 10, B–D (*N* = 3). (**D**) HEK293 cells were transfected with the following vectors: empty (GFP), GFP-WIPI2 or WIPI2 mutants (GFP-RERE [R108E/R128E], GFP-FTTG, or double mutant). Immunoprecipitation was performed as in **B** and **C**, and endogenous BCAS3 or MYTHO was blotted. (**E**) Left: Representative fluorescence images of endogenous WIPI2 protein in *Mytho*-KO cells transfected with GFP or MYTHO-GFP vector. Scale bars: 20 μm. Right: Quantification of WIPI2 puncta/cell in Fed and 2-hour starved (STV) (*N* = 3) using ImageJ software. Fed WT + GFP (*n* = 34); Fed *Mytho*-KO + GFP (*n* = 69), Fed *Mytho*-KO + MYTHO-GFP (*n* = 49); HBSS 2h WT + GFP (*n* = 12); HBSS 2h *Mytho*-KO + GFP (*n* = 53), HBSS 2h *Mytho*-KO + MYTHO-GFP (*n* = 33) (1-way ANOVA on ranks [Kruskal-Wallis test]). (**F** and **G**) WT and *Mytho*-KO C2C12 cells were transfected with empty (GFP), MYTHO-GFP (WT), M1-GFP, M3-GFP, M5-GFP, or M3/M5-GFP vector. The quantification of endogenous LC3 (**F**) or WIPI2 (**G**) puncta in the fed condition was performed using ImageJ software (*N* = 3). For LC3 puncta: WT + GFP (*n* = 91); *Mytho*-KO + GFP (*n* = 101), *Mytho*-KO + WT (*n* = 84); *Mytho*-KO + M1 (*n* = 41); *Mytho*-KO + M3 (*n* = 48), *Mytho*-KO + M5 (*n* = 40), *Mytho*-KO + M3/M5 (*n* = 53) (1-way ANOVA with Tukey’s multiple-comparison test). For WIPI2 puncta: WT + GFP (*n* = 107); *Mytho*-KO + GFP (*n* = 119), *Mytho*-KO + WT (*n* = 98); *Mytho*-KO + M1 (*n* = 65); *Mytho*-KO + M3 (*n* = 115), *Mytho*-KO + M5 (*n* = 50), *Mytho*-KO + M3/M5 (*n* = 56) (1-way ANOVA on ranks [Kruskal-Wallis test]). All bars indicate SEM. **P < 0.001; ****P < 0.0001. *N* = number of independent experiments; *n* = number of samples.

To identify the regions of interaction with LC3 and WIPI2, we mutagenized the putative LIR or WD40 domain of MYTHO (Figure 7A) and performed immunoprecipitation experiments. By pulling down the different MYTHO mutants we found that the interaction with lipidated LC3B was abolished when motif 1 (M1) was mutated (Figure 7B and Supplemental Figure 10B). Interestingly, when we checked for the presence of WIPI2 in the MYTHO-immunoprecipitated complex, we showed a reduction in WIPI2 binding to MYTHO when motif 3 (M3) and 4 (M4) were altered, and a slight reduction with mutated M1 (Figure 7B and Supplemental Figure 10C). BCAS3 interaction was also lost by altering M3 and M4 (Figure 7B and Supplemental Figure 10D). Since the mutagenesis of M3 disrupted 1 putative WD40 out of 2, we also mutagenized this second site (M5) and both M3 and M5 motifs and found a reduction in WIPI2 as well as ATG16L1 binding and the absence of BCAS3 interaction (Figure 7C). Thus, M1 is important for the LC3B interaction, while M3 and M4/M5 modulate the WIPI2 interaction. To establish whether the MYTHO-WIPI2 interaction occurs on the PtdIns3P-enriched membrane, we expressed in cells a WIPI2 mutant (WIPI2-FTTG), which is unable to bind PtdIns3P, for pull-down experiments. Interestingly, the WIPI2-FTTG/MYTHO complex was preserved, suggesting that this interaction happened independently of WIPI2 recruitment at the PtdIns3P-enriched membranes (Figure 7D). Next, we asked whether the interaction of MYTHO with WIPI2 depends on the ability of WIPI2 to bind ATG16L. By pulling down the WIPI2-RERE mutant, which is unable to bind to ATG16L1, we could not detect endogenous MYTHO (Figure 7D). Thus, MYTHO binds the WIPI2-ATG16L complex independently of the recruitment of WIPI2 to the PtdIns3P-enriched membranes.

To further support the direct role of MYTHO in the recruitment of the WIPI2 complex to the phagophore, we restored MYTHO protein in KO cells and found that WIPI2 puncta were reestablished in the absence of nutrients (Figure 7E). Consistently, the rescue of MYTHO protein in KO cells restored the levels of LC3-positive puncta (Figure 7F). Finally, when we expressed the M1 and M3 mutants, which showed a reduced LC3 and WIPI2 binding, respectively, WIPI2 puncta were restored by M1 expression (Figure 7G), while LC3 puncta were not rescued by any of the mutants (Figure 7F). Consistently, expression of mutant M5 or M3/M5 did not rescue WIPI2 puncta (Figure 7G and Supplemental Figure 10E). Altogether, these results show that MYTHO plays a fundamental role in WIPI2 recruitment at the phagophore site and in autophagosome formation.

### MYTHO acts in different longevity pathways.

**Figure 8:**
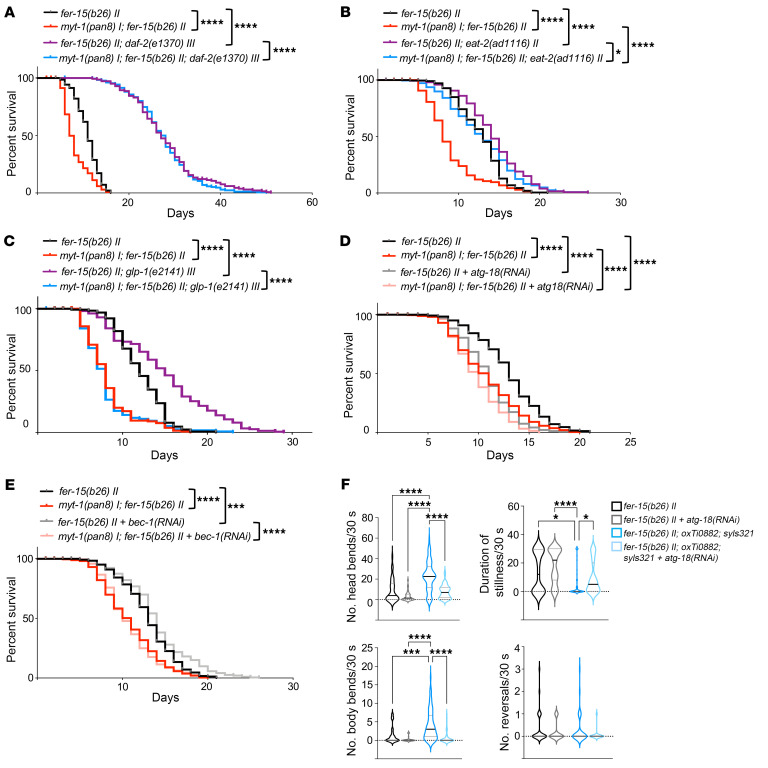
*myt-1* controls longevity through the *eat-2* and *glp-1* signaling pathways. (**A**) Survival curves of *fer-15(b26)* *II* (*n* = 140), *myt-1(pan8) I; fer-15(b26) II* (*n* = 140), *fer-15(b26) II; daf-2(e1370) III* (*n* = 141), and *myt-1(pan8) I; fer-15(b26) II*, *daf-2(e1370) III* worms (*n* = 179) (*N* = 2). (**B**) Survival curves of *fer-15(b26)* *II* (*n* = 216), *myt-1(pan8) I; fer-15(b26) II* (*n* = 244), *fer-15(b26) II; eat-2(ad1116) II* (*n* = 171), and *myt-1(pan8) I; fer-15(b26) II; eat-2(ad1116) II* (*n* = 263) worms (*N* = 2/3). (**C**) Survival curves of *fer-15(b26)* *II* (*n* = 135), *myt-1(pan8) I; fer-15(b26) II* (*n* = 162), *fer-15(b26) II; glp-1(e2141) III* (*n* = 163), *myt-1(pan8) I; fer-15(b26) II; glp-1(e2141) III* (*n* = 162) (*N* = 2). Raw data of *fer-15(b26)* *II* and *myt-1(pan8) I; fer-15(b26) II* worms are the same in **B** and **C** (experiments were performed in parallel). Cox proportional hazards analysis was performed for the interaction of terms genotypes *myt-1* and *daf-2* (0.00018), *eat-2* (0.00004), *glp-1* (0.0007). (**D** and **E**) Life span of young adult *fer-15(b26)* *II* and *myt-1(pan8) I; fer-15(b26) II* worms fed with empty pL4440 vector or pL4440 expressing the *atg-18* coding sequence [*atg-18(RNAi)*] (*n* = 260–340 worms/condition) (**D**) or *bec-1* coding sequence [*bec-1(RNAi)*] (*n* = 228–290 worms/condition) (**E**) following the adulthood RNAi protocol (see Methods) (*N* = 3). Cox proportional hazards analysis was performed for the interaction of terms genotypes *myt-1* and *atg-18* RNAi (*P* < 0.0001), *bec-1* RNAi (*P* = 0.01466). Raw data of *fer-15(b26)* *II* and *myt-1(pan8) I; fer-15(b26) II* worms are the same in **D** and **E** (experiments were performed in parallel). Log-rank test was used to compare longevity curves (see Supplemental Figure 6A for life span experimental details and statistics). (**F**) Body and head bends, reversals, and duration of stillness periods were quantified for 30 seconds in 10-day-old *fer-15(b26) II* animals (WT) and *fer-15(b26) II; oxTi0882; syls321* (OE *myt-1*) worms fed with *atg-18(RNAi)* (*n* = 38 [WT]/*n* = 51 [OE *myt-1*]) or control bacteria (*n* = 33 [WT]/*n* = 76 [OE *myt-1*]) after a harsh touch stimulus at the tail (*N* = 2). *P < 0.05; ***P < 0.001; ****P < 0.0001. *N* = number of independent experiments; *n* = total worm number.

Since *myt-1* inhibition reduced worm survival, we investigated whether *myt-1* is required in any longevity pathway by genetic interaction experiments. Loss-of-function mutations in the insulin receptor *daf-2* increase life span through the activation and translocation to the nucleus of the transcription factor DAF-16 (FoxO) (2, 4). To establish whether *myt-1* mediates the insulin-dependent effect on longevity, we crossed *daf-2(e1370)* *III* animals with *myt-1*–deficient worms and checked life span. Importantly, *myt-1* ablation did not suppress the extended life span of *daf-2(e1370) III* mutants, and therefore is not required for *daf-2*–mediated longevity (Figure 8A and Supplemental Figure 6A). Similar results were obtained when *daf-2* was knocked down in adult *myt-1* mutants (Supplemental Figure 6B and Supplemental Figure 11A). Next, we used a pharyngeal pumping–defective *eat-2* mutant [namely, *eat-2(ad1116) II*], which mimics caloric restriction due to its reduced food intake, leading to extended life span (25). Ablation of *myt-1* significantly reduced the longevity of *eat-2(ad1116)* mutants, suggesting that it was partially indispensable for life span extension due to dietary restriction (Figure 8B and Supplemental Figure 6A), but with a smaller effect size compared with its effect on WT worms, as confirmed by Cox proportional hazards (CPH) analysis (*P* = 0.00004). The Notch family receptor *glp-1* mediates Notch signaling and controls the mitotic proliferation of germline cells (26, 27). The *glp-1(e2141) III* strain carries a *glp-1* loss-of function mutation and shows prolonged life span when maintained at the nonpermissive temperature due to failed germline proliferation (28). Importantly, life span extension of the *glp-1(e2141)* mutant was completely abolished when crossed with *myt-1* mutants, suggesting a critical function of this gene in the longevity pathways activated by *glp-1* deletion (Figure 8C and Supplemental Figure 6A). Similar results were obtained by knocking down *glp-1* in *myt-1* mutants (Supplemental Figure 6B and Supplemental Figure 11B). Altogether, these results indicate that *myt-1* is required for *glp-1*–mediated life span and partially indispensable for *eat-2*–mediated longevity.

Finally, we explored the involvement of MYTHO in the autophagy-mediated effects on life span and specifically on the WIPI2 pathway and BECN1 signaling. Our findings showed that MYTHO interacted with WIPI2, but not with BECN1. We knocked down *atg-18* or *bec-1*, the nematode homologs of human *WIPI2* and *BECN1* genes respectively, in WT and *myt-1*–KO worms and measured their survival. Knockdown of *atg-18* resulted in a decreased life span of control and *myt-1*–KO animals. Interestingly, the reduction in life span was less evident in the *myt-1*–mutant background (Figure 8D and Supplemental Figure 6B). CPH analysis showed a statistically significant interaction between *myt-1* deletion and *atg-18* knockdown (*P* < 0.0001), supporting an epistatic link between ATG-18 and MYT-1. Besides their influence on worm life span, the epistatic interaction between MYTHO and WIPI2 was further supported by other analyses. Indeed, the improved locomotive activity induced by *myt-1* overexpression in muscles [*fer-15(b26) II; oxTi0882; syls321*] was blunted when *atg-18* was knocked down (Figure 8F). Interestingly, knocking down *bec-1* did not affect the survival curves of *myt-1*–KO worms in a significant manner, while having a minor effect on extending WT worm longevity (Figure 8E and Supplemental Figure 6B). However, CPH analysis between *bec-1* knockdown and *myt-1* deletion suggested a minor but significant epistatic interaction between BEC-1 and MYTHO (*P* = 0.015). To verify *bec-1* silencing efficiency, we fed worms that expressed a GFP-tagged version of BEC-1 (FR758 strain) with bacteria expressing dsRNA for *bec-1* (see Supplemental Figure 11C and Methods for strain details), and noticed a clear reduction in GFP signal compared with controls. Moreover, long-lived *eat-2(ad1116)* mutants, when fed with bacteria expressing dsRNA *bec-1*, showed a decrease in life span (Supplemental Figure 6B and Supplemental Figure 11D), as previously reported (29). Overall, these data are consistent with *myt-1* being downstream of the WIPI2/ATG-18 pathway.

**Figure 9:**
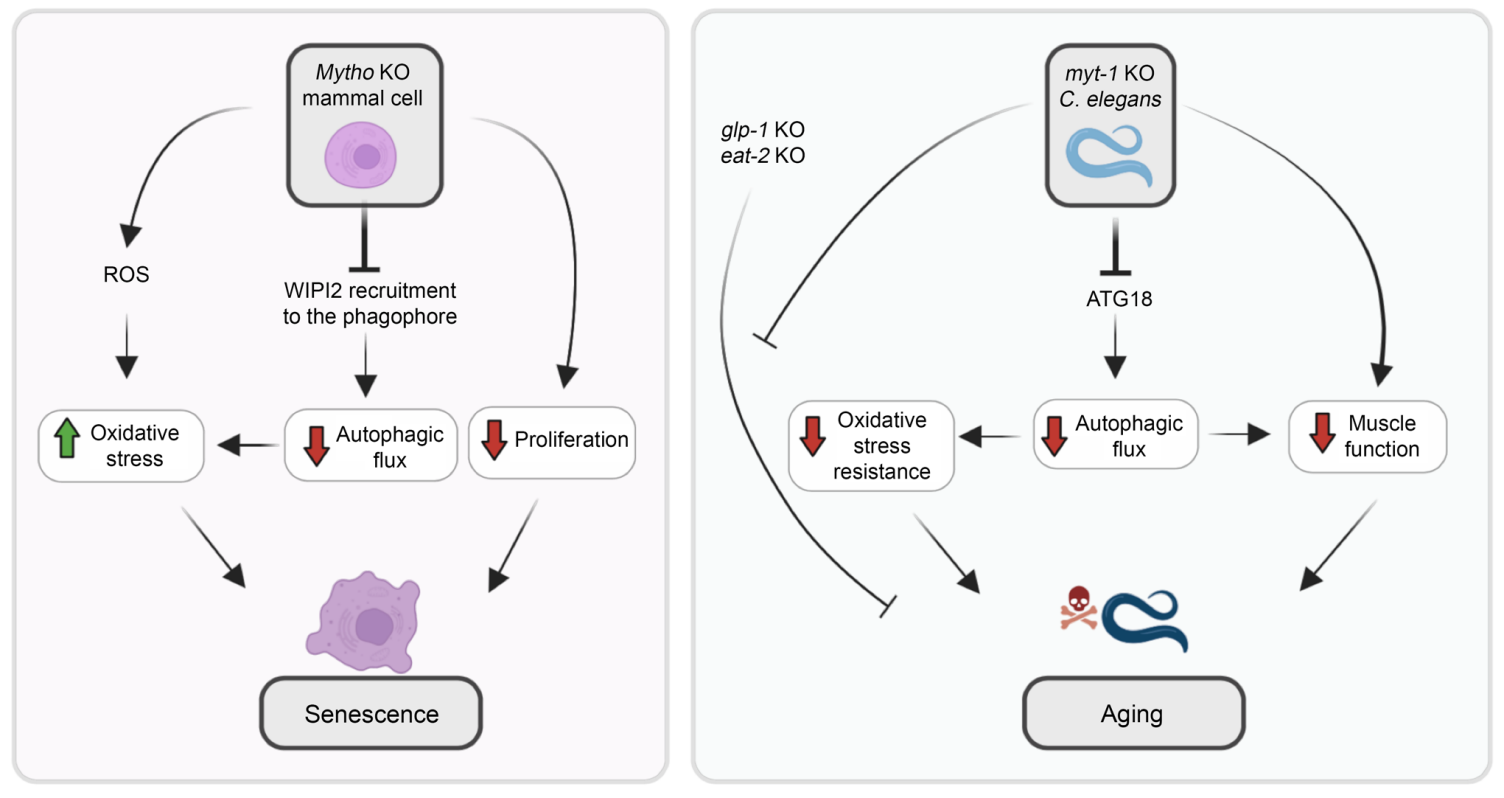
Scheme of MYTHO function in mammalian cells and *C*. *elegans*. Left panel shows the effects of MYTHO inhibition in mammalian cells. The ablation of the *Mytho* gene causes autophagic impairment, mitochondrial dysfunction with increased ROS production, accumulation of β-galactosidase, upregulation of p21, and reduced cell proliferation. These features belong to the hallmarks of aging, supporting a MYTHO role in preventing cellular senescence. The right panel describes the consequences of *myt-1* deletion in *C*. *elegans*. Consistently, autophagic flux, resistance to oxidative stress, life span, and health span were reduced in the absence of *myt-1*. The *myt-1* contribution to life span was dissected by genetic interaction studies that identified *myt-1*’s involvement in *glp-1*– and *eat-2*–mediated longevity.

In conclusion, we determined the function of an uncharacterized gene that is fundamental for life span and health span, being involved in WIPI2 recruitment at the phagophore site and in autophagosome formation. Our findings about MYTHO’s function in mammalian cells and in *C*. *elegans* are summarized in Figure 9.

## Discussion

The identification of genes that confer either extension of life span or accelerate age-related decline has been a step forward in our understanding the mechanisms of senescence and revealed that the aging process is partially controlled by genetics. However, this genetic contribution is only partially understood, likely because many genes are not yet clearly characterized for their possible role in maintenance and repair processes ensuring short or long life spans. To investigate the role of *myt-1* in pro-longevity interventions, we tested the long-lived *glp-1(e2141) III* (which shows reduced proliferation of germline cells) and *eat-2(ad1116) II* (a genetic dietary restriction model) mutants. The findings that the absence of *myt-1* completely blunted the life extension of *glp-1* mutants and partially affected the longevity of *eat-2* mutants suggest that *myt-1* is mediating the response to germline signals and dietary cues, respectively (see Figure 8 legend for CPH analysis of interaction of terms *myt-1* and other genotypes) (Figure 9). Thus, *myt-1* is required for both natural longevity and in specific pro-longevity interventions. Mechanistically, we found that MYTHO/*myt-1* played a critical role in autophagy regulation and particularly, in WIPI2-ATG16L and ATG7 recruitment on the phagophore under stress conditions. Consistent with this hypothesis, MYTHO localized at DFCP1-positive sites. Moreover, *Mytho*-deficient cells showed a significant reduction in WIPI2-positive puncta and autophagosome formation upon nutrient deprivation. The interactome as well as immunoprecipitation and rescue experiments confirmed that MYTHO binds the WIPI2-ATG16L complex via the WD40 domains. Our findings are supported by 2 other independent studies in which MYTHO was found in the interactome of the autophagy protein WIPI2 (Atg18) (30, 31).

WIPI2 functions as a PtdIns3P effector, bridging PtdIns3P production with the recruitment of the ATG5-ATG12-ATG16L complex to permit the covalent binding of LC3B/ATG8 to phosphatidylethanolamine (lipidation reaction) (32). When we mutagenized WIPI2 to hinder its binding to PtdIns3P, we still detected the interaction with MYTHO, suggesting that the complex is formed independently of the recruitment on PtdIns3P-enriched membranes. Importantly, this interaction was disrupted when WIPI2 was mutagenized in the domain for ATG16L binding, suggesting that MYTHO binding happens only when the WIPI2-ATG16L complex is formed. Because *Mytho* ablation did not completely suppress autophagy as well as ATG16L puncta, other mechanisms for the conjugation system recruitment also exist and synergize with the MYTHO-WIPI2 complex to maximally activate the E3 enzyme and the lipidation process (33). For instance, ATG16L has been reported to bind the ATG1-ULK1 complex via FIP200 (34, 35), and directly to PtdIns3P-enriched membranes (33, 36, 37). However, the striking phenotype of worms and the effect on mitochondria, cellular senescence, and oxidative stress underline the important physiological function of *MYTHO* (Figure 9). Since mutations of *WIPI2* caused multiple-organ defects in humans with a premature aging phenotype (38), it will be interesting to explore whether mutations of *MYTHO* also cause disease onset in humans. Interestingly, *MYTHO* has been reported to be fused in frame with *ABCC6* and *ARL16* genes in acute myeloid leukemia and lung squamous cell carcinoma, but the pathogenetic role of the fused transcripts has not yet been explored (39). Consistently, we recently found that when *Mytho* expression was inhibited chronically it resulted in muscle degeneration and myopathy (40). Some of the myopathic features differ from the ones that characterize autophagy failure, suggesting that *MYTHO* is involved in other cellular biological processes that are critical for cell survival. This hypothesis is also supported by the finding that *myt-1* ablation did not shorten the life span of the long-lived *daf-2(e1370)*, while the autophagy genes are required for the life span extension of this mutant (11, 12). This discrepancy suggests that *myt-1* could be involved in other longevity-related functions that are autophagy independent and that will be investigated in future studies.

## Methods

Additional methods are provided in the Supplemental Methods.

### Plasmid cloning

The murine *Mytho* coding sequence (1,239 bp) was amplified by cDNA obtained from skeletal muscles of tumor-bearing mice and cloned in the p3XFlag-Myc-CMV vector (6.4 kb) (Addgene) using KOD Hot Start DNA polymerase (Merck Millipore) and the following primers with sticky ends: Fw 5′-AAAGATCTACTGGACCTGGAGGTGGT-3′ and Rw compl. 5′-TTTGATATCTTAGGGCAGCTCTGCTGTTCT-3′. Vector and insert were digested using the restriction enzymes BglII and EcorV with buffer 3 (New England Biolabs) at 37°C for 2 hours and the digested vector and insert were purified after having excised bands from 1% agarose gels with NucleoSpin Gel and PCR Clean-up (Macherey-Nagel).

The *Mytho* gene was also subcloned in the pEGFP-N3 vector (4.7 kb) (Addgene) with KOD Hot Start DNA polymerase using the following primers with sticky ends: Fw 5′-AAAGCTAGCATGCTGGACCTGGAGGTGGT-3′ and Rw compl. 5′-TAAGGATCCGGGCA GCTCTGCTGTTC-3′.

To monitor LC3-II puncta, we also subcloned *Mytho* in a PBI3xFlag vector that contains a YFP-LC3 expression gene in a different cloning site (PBI YFPLC3-3xFlagMYTHO, Addgene) using KOD Hot Start DNA polymerase. We designed the following primers with sticky ends: Fw 5′-AAAGCTAGCATGCTGGACCTGGAGGTGGT-3′ and Rw compl. 5′-GGTGATATCTTAGGGCAGCTCTGCTGTTCTCA-3′. Vector PBI and insert were digested using the restriction enzymes NheI-HF and EcorV-HF (New England Biolabs) at 37°C for 2 hours and the digested vector and insert were purified after having excised bands from 1% agarose gels using NucleoSpin Gel and PCR Clean-up. Fifty nanograms of vector with 3-fold molar excess of insert were ligated using the Quick Ligation Kit (New England Biolabs).

To generate stable cell lines, *MYTHO* cDNA was cloned into the pDONR223 vector (Addgene) by using the BP Clonase Reaction Kit (Thermo Fisher Scientific) and further recombined into the lentiviral GATEWAY destination vector pLenti-UBC-gate-3xHA-pGK-PUR (107393, AddGene).

Other plasmids used in this project were Cherry-LC3B, LAMP2-Cherry, Golgi-GFP, GFP-WIPI2, ATG16L1-Flag, GFP-WIPI2b RERE (R108E/R128E) mutant, and GFP-WIPI2b FTTG mutant provided in-house (32).

### *C. elegans* strains, growth conditions, and maintenance

The strains Bristol N2 (WT), DH26 [*fer-15(b26) II*], DA2123 [*lgg-1p:GFP:lgg-1+rol-6(su1006)*], MAH215 (*sqIs11* [*lgg-1p:mCherry:GFP:lgg-1 + rol-6*]), DA1116 [*eat-2(ad1116)* *II*], CB4037 [*glp-1(e2141) III*], and CB1370 [*daf-2(e1370) III*] were obtained from the *Caenorhabditis* Genetics Center (University of Minnesota, Minneapolis, Minnesota, USA). FR758 strain (*swEx520[pbec1:BEC-1:GFP + rol-6(su1006)]*) was a gift from Tibor Vellai (Department of Genetics, Eötvös Loránd University, Budapest, Hungary). Strains were grown on nematode growth media (NGM) agar plates at 20°C (or 25°C where indicated), seeded with *E*. *coli* OP50 [or HT115(DE3)] bacteria and genetic crosses were performed as described previously (41). When indicated, age of worms refers to specific larval stage L4, young adult stage, or reproductive adults, starting to count from day 1 of adulthood.

### Genome editing in *C. elegans*

#### Generation of myt-1(pan8) I and myt-1(pan9) I strains.

The human gene *MYTHO* has 1 ortholog in *C*. *elegans*, that is, *T01G9.2* (herein referred to as *myt-1*), which displays 2 isoforms (a [NM_171841.9] and b [NM_059851.6]) differing in the 3 amino acids KFK, from position 21 to 23, that are present only in isoform a.

Genetic ablation of *myt-1* was obtained using CRISPR/Cas9 technology with a modified protocol (42). Briefly, 20 WT animals were injected with a mix containing 750 ng/μL Cas9 (Integrated DNA Technologies, Inc. [IDT]), 700 ng/μL ALT-R CRISPR tracrRNA (IDT), 115 ng/μL dpy-10 crRNA, 37.5 ng/μL ssODN dpy-10, 400 ng/μL *T01G9.2* crRNA (5′-TGAAGAAGATCTGAGCTTCA-3′), and 175 ng/μL of the *T01G9.2* KO ssODN (5′-CATCGAAAATGAATGGCAAACAGCAAGTTACAAAATAACCGTCGACTGAGGAAGACCTAAGCTTCACGTTTGTTTTAAAGTCAAAAAATCAATAATAA-3′), recovered in M9 buffer and incubated at 20°C. Animals with roller or dumpy phenotypes were isolated, as well as pools of 5 WT worms from those plates. To isolate mutant animals, PCR amplification was performed using a single forward primer (5′-TGAAAAGTCGATAAAAATTCAGTAGCA-3′) and 2 reverse primers annealing specifically with the mutated (5′-CTCAGTCGACGGTTATTTTGTA-3′) or the WT sequence (5′-ACCTTTTTACTGTACTTCAATTCGACT-3′). Homozygosity was confirmed by Sanger sequencing using standard techniques. Four null strains were generated. Three of them carried a frameshift mutation predicted to lead to the formation of a premature stop codon at position 36, i.e., *T01G9.2*(*pan8*[S18Tfs*19]); *T01G9.2*(*pan9*[S18Tfs*19]); *T01G9.2*(*pan10*[S18Tfs*19]). Independent strains *T01G9.2*(*pan8*[S18Tfs*19]) and *T01G9.2*(*pan9*[S18Tfs*19]), renamed *myt-1(pan8) I* and *myt-1(pan9) I*, were outcrossed twice to remove possible off-target mutations and used for phenotypic analyses. In particular, experiments were performed using the *myt-1(pan8) I* strain and some results were confirmed using *myt-1(pan9) I*.

#### Generation of a myt-1 translational GFP reporter strain.

The *issEx1 [myt-1p:gfp]* transgenic line was obtained as described previously (43). Briefly, 501 and 864 bp of 5′UTR immediately upstream of the *myt-1* first ATG codon were tested as putative promoter regions. Both sequences were amplified from worm genomic DNA using a Rw primer that contains a 24-nucleotide overlap with the *gfp* sequence. In parallel, the coding sequence of *gfp* and the 3′UTR of the *unc-54* gene were amplified from pPD95.75 vector (Fire Kit, Addgene). The 2 amplicons were then fused by PCR and the correctness of the final product was checked by agarose gel. Five fusion-PCR products were pooled together and injected in the gonads of young adult N2 worms. GFP^+^ worms were observed only with the fusion product containing the longer *myt-1* 5′UTR. These were then selected and isolated to verify transgene transmission in the progeny. Three independent lines were generated and used to analyze *myt-1* expression.

#### Generation of a body wall muscle myt-1–overexpressing line.

We purchased from the Genome Engineering Facility of the Max Planck Institute of Molecular Cell Biology and Genetics (Dresden, Germany) the body wall muscle driver strain PS6936 (*syIs321 [myo-3p:NLS:GAL4SK:VP64:unc-54 3′UTR* + *myo-2p:NLS:mCherry* + *pBlueScript]*) that expresses an mCherry reporter in the pharyngeal muscles (44) and the effector strain *oxTi10882* [*15xUAS-T01G9.2a-SL2-mScarlet-glh-2_3′UTR*], carrying a transgene inserted in chromosome IV consisting of the *myt-1* coding sequence (isoform a) downstream of the UAS element with mScarlet reporter. Progeny resulting from the cross of driver and effector strains is recognizable by the mScarlet fluorescence in body wall muscles. Then, *oxTi10882; syIs321* worms were crossed with DH26 *fer-15(b26) II* animals that have a temperature-sensitive defect in spermatogenesis and are thus sterile at 25°C, in order to obtain *fer-15(b26) II; oxTi10882; syIs321* worms. Two independent lines were generated and used in the experiments.

### Generation of *Mytho*-KO C2C12 cell line using CRISPR/Cas9

The C2C12 cell line was purchased from ATCC and cells were grown in Dulbecco’s modified Eagle medium (DMEM) supplemented with 10% fetal bovine serum (FBS; heat inactivated at 55°C for 1 hour), 1% penicillin/streptomycin, and 1% L-glutamine (reagents for cell cultures were purchased from Gibco/Thermo Fisher Scientific).

To generate the *Mytho*-KO C2C12 line, cells were cotransfected with Transedit CRISPR all-in-one lentiviral expression vectors (pCLIP-ALL-EFS-Puro) containing 2 different CRISPR target sequences of murine *Mytho* (TEVM-1183975 and TEVM-1251117, Transomic Technologies), targeting exon 1 and exon 2, respectively. Transfections were performed using Lipofectamine 2000 (Thermo Fisher Scientific), according to the manufacturer’s protocol.

After 24 hours, cells were selected by the addition of 1 μg/mL puromycin (Gibco/Thermo Fisher Scientific) to the culture medium until the untransfected control cells were all nonviable. To isolate single clones, cells were serially diluted and seeded in 96-well plates. After growth and expansion of clones, genomic DNA was extracted from cells using standard protocols and fragments encompassing the CRISPR target sequences were amplified by PCR. Two different PCR reactions were performed, the first with 2 primers upstream and downstream of the first gRNA target (Fw primer 5′-CCACTTTTGCTGCAGTTGCT-3′ and Rw primer 5′-TGCTGAGACATCGCTGATCC-3′) and the second with the same forward primer and as reverse an oligonucleotide downstream of the second gRNA target (5′-TGAAAAGGCCCCCATGTGAA-3′). PCR reactions were then sequenced and 4 different clones harboring truncating mutations were mixed to reduce the consequences of possible CRISPR/Cas9-mediated off-target effects.

### Endogenous HA tagging of MYTHO in HeLa cells using CRISPR/Cas12

HeLa cells were purchased from ATCC and grown in DMEM supplemented with 10% FBS, 1% penicillin/streptomycin, and 1% L-glutamine. Cells were constantly monitored for mycoplasma contamination. To generate the endogenous HA tag at the C-terminus of MYTHO, we used the protocol described in Fueller et al. (23). Briefly, oligonucleotides were designed (http://www.pcr-tagging.com/) to generate the PCR cassette M1_Mytho:5′-CACCAGGTCATGCAGAACAACCACATTGCCTCGGTGACCCTGTATGGCCCCCCCAGGCCTGGTAGCCACCTGAGAACAGCGGAACTCCCCT CAGGTGGAGGAGGTAGTG-3′M2_Mytho_LbCpf1_TYCV:5′-GAGCAGGATGTGATGCACAGTTCCACGGGACAGAGGGGCATGGGTGGTGGTGTCCAAAAAAATGGGTGGTGGTGTCCCTAGATCT ACACTTAGTAGAAATTAGCTAGCTGCATCGGTACC-′3.

As template of the PCR cassette, plasmid pMaCTag-P27(1×HA) (Addgene) was used. Cells were transfected with the PCR cassette and Cas12 plasmid pcDNA3.1-hLbCpf1(TYCV) (pY230) (Addgene) using Lipofectamine 2000.

Forty-eight hours after transfection, cells were selected by the addition of 2 μg/mL puromycin over a period of 2 weeks. To exclude nonspecific tagging, cells were tested by genomic extraction followed by a PCR amplification of segments targeting the predicted integration of HA at the MYTHO C-terminus. PCR products were analyzed by gel electrophoresis. In addition, the integration was also verified by Western blot, testing cell lysates with an anti-HA antibody (3724, Cell Signaling Technology).

### Generation and propagation of MYTHO-HA stable and inducible cell lines

Stable cell lines were produced using lentiviral infection. Viruses were produced using HEK293T cells, purchased from ATCC. HeLa stable cell lines were generated cloning the cDNAs into pLenti-UBC-gate-3xHA-pGK-PUR lentiviral vector carrying a 3×HA tag at the C-terminus (107393, Addgene).

### Immunofluorescence analysis

Cells were fixed in 4% paraformaldehyde in PBS for 10 minutes, permeabilized with 0.3% Triton X-100 in PBS for 2 minutes, and then blocked using 0.5% bovine serum albumin (BSA)/10% goat serum in PBS. Slides were incubated for 24 hours at 4°C with primary antibodies (1:100) and after 3 washes with secondary antibodies (1:200) for 1 hour at room temperature. Nuclei were stained with Hoescht and mounted with fluorescent mounting medium (Dako Omnis, Agilent). The following primary antibodies were used: anti-FLAG M2 (F3165, Sigma-Aldrich/Merck), anti-C16orf70 (ab181987, Abcam), anti-WIPI2 (purified mouse IgG clone 2A2 from the Tooze lab), anti-HA (H3663, Sigma-Aldrich/Merck), anti-VDAC (D73D12, Cell Signaling Technology), anti-PDI (C81H6, Cell Signaling Technology), anti-PMP70 (P0497, Cell Signaling Technology), and anti-BCAS3 (ab71162, Abcam). Secondary antibodies were anti-rabbit or anti-mouse Cy3, Alexa Fluor 647, or Alexa Fluor 488 (Jackson Immunoresearch).

### Immunoblotting

Forty tibialis anterior cryosections of 20 μm thickness were lysed in 100 μL of lysis buffer (50 mM Tris pH 7.5, 150 mM NaCl, 10 mM MgCl_2_, 0.5 mM DTT, 0.5 mM EDTA, 10% glycerol, 2% SDS, 1% Triton X-100), protease inhibitor cocktail and phosphatase inhibitors cocktail I and II (Roche). Protein quantification was determined using a Pierce BCA kit (Thermo Fisher Scientific).

C2C12 myoblasts or HEK293 cells were washed and lysed with 100 μL RIPA buffer supplemented with protease inhibitors and phosphatase inhibitors (Roche). Cells were incubated in lysis buffer on ice before being scraped and transferred into a clean Eppendorf tube. After 15 minutes of centrifugation at 15,000*g*, the supernatant was collected for quantification with a Pierce BCA kit.

Muscle protein lysate (30 μg) and 10 μg or 20 μg of cell lysate were loaded in SDS-PAGE gels (Thermo Fisher Scientific) and electroblotted onto nitrocellulose membranes. Transfer buffer (Bio-Rad Laboratories) was prepared using 10× Tris-glycine and 20% methanol in H_2_O. Membranes were saturated with blocking buffer (5% nonfat milk powder solubilized in 1× TBS with 0.1% Tween).

The following antibodies were used: anti-GAPDH (32233, Santa Cruz Biotechnology), rabbit anti-FLAG (F7425, Sigma-Aldrich/Merck) or M2 (F3165, Sigma-Aldrich/Merck), anti-LC3B (L7543, Sigma-Aldrich/Merck), anti-WIPI2 (purified mouse IgG clone 2A2 from the Tooze lab), anti-GFP (A11122, Thermo Fisher Scientific), anti-C16orf70 (ab181987, Abcam), anti-ATG16L1 (D6D5, Cell Signaling Technology), anti–Beclin 1 (D40C5, Cell Signaling Technology), anti-ATG7 (D12B11 Cell Signaling Technology), anti-HA (C29F4, Cell Signaling Technology), and anti-BCAS3 (ab71162, Abcam).

### MYTHO site-directed mutagenesis

LIR or WD40 domains were identified by using the iLIR (https://ilir.warwick.ac.uk) (45) and http://elm.eu.org/ (46) databases. Site-directed mutagenesis of some motifs in the MYTHO-GFP plasmid was performed by using a Q5 site-directed mutagenesis kit (New England Biolabs) according to the manufacturer’s instructions. The following primers were designed to mutagenize the crucial amino acids: M1 mutation Y91A/V94A, Fw 5′-AAAGTAAAGTTAAAGGCTTGTGGAGCTCATTTTAACTCTCAGGCC-3′; M2 mutation F131A/L134A, Fw 5′-CTCTTCCACCTCAACGCTCGAGGAGCTTCTTTCTCTTTTCAG-3′; M3 mutation Y288A/L291A, Fw 5′-GACTACTTTTTTAACGCTTTTACTGCTGGAGTGGACATCCTG-3′; M4 mutation W351A/I354A, Fw 5′-ACAACCTACAGCAAGGCTGACAGCGCTCAGGAGCTTCTG-3′; M5 deletion, 208delTGPSGLRLRL Fw 5′-CGCTTGCTCGCTGCAGGTTGTGGA-3′; and Rw compl. 5′-TCCATCTCGAAGAACGTCTACACTTTCAGCA-3′; M3 and M5 mutation (both WD40 regions): Y288A/L291A + 208delTGPSGLRLRL using the primers described above. PCR conditions for gene amplification were 98°C for 30 seconds; then 25 cycles at 98°C 10 seconds, 72°C for 30 seconds, and 72°C for 2.5 minutes; and final extension at 72°C for 2 minutes. After PCR, the product was incubated with the Kinase-ligase-DpnI (KLD) enzyme mix (New England Biolabs) for 5 minutes at room temperature for rapid circularization and template removal. Transformation was performed using the high-efficiency NEB 5-alpha Competent *E*. *coli* (New England Biolabs).

### Immunoprecipitation

Cells (3 × 10^6^) were seeded in 10-cm dishes for Lipofectamine 2000 transfection. Twenty-four hours after transfection, cells were lysed using 1 mL of TNTE lysis buffer (20 mM Tris-HCl pH 7.4, 150 mM NaCl, 5 mM EDTA, and 0.3% Triton X-100) supplemented with 1× PhosSTOP (Roche) and 1× EDTA-free Complete Protease Inhibitor Cocktail (Roche). After lysis, cells were centrifuged at 16,100*g* for 10 minutes at 4°C and 10 μL of supernatant was used as 1% input for Western blot analysis. The remaining sample was immunoprecipitated using 10 μL of GFP-TRAP beads from the iST GFP-Trap kit (ChromoTek/Proteintech Group, Inc.). GFP beads were washed 4 times with buffer TNTE before immunoprecipitation. Lysate was incubated with GFP-TRAP beads for 2–3 hours, rotating at 4°C. Then, beads were centrifuged and 0.5% or 1% of the unbound fraction was used for immunoblot analysis. All immunoprecipitation samples were washed 4 times with TNTE buffer before adding 30 μL of 2× SDS sample buffer and boiling for 5 minutes at 100°C. After centrifugation of the beads, 20 μL of the supernatant was loaded for immunoblot analysis.

### Life span analysis

#### Experiments with mutant lines.

Life span of *myt-1(pan8) I* and *myt-1(pan9) I* worms was firstly assessed compared to Bristol N2 animals. Moreover, *myt-1(pan8) I*, *myt-1(pan9) I*, *daf-2(e1370)* *III*, *eat-2(ad1116)* *II*, and *glp-1(e2141) III* worms were crossed with *fer-15(b26) II* animals. *myt-1(pan8) I; fer-15(b26) II* were then crossed with *eat-2(ad1116) II, glp-1(e2141) III*, or *daf-2 (e1370) III* strains in order to obtain *myt-1(pan8) I; myt-1(pan8) I; fer-15(b26) II, daf-2(e1370) III, myt-1(pan8) I; fer-15(b26) II; eat-2(ad1116) II,t-1(pan8) I; fer-15(b26) II; glp-1(e2141) III* worms.

#### RNAi experiments.

The *myt-1* coding sequence (which included both isoforms) was cloned into the pL4440 empty vector (Fire Kit, Addgene) [*myt-1(RNAi)*], the *atg-18* RNAi clone (V-14D09) was purchased from Source BioScience, and the *bec-1* RNAi clone was a gift from Julián Cerón Madrigal (Modeling Human Diseases in *C*. *elegans* Group; Genes, Disease and Therapy Program, Institut d’Investigació Biomèdica de Bellvitge - IDIBELL, L’Hospitalet de Llobregat, Barcelona, Spain). Plasmids pAD48-*daf-2* RNAi (Addgene plasmid 34834; http://n2t.net/addgene:34834) and pAD12 (Addgene plasmid 34832; http://n2t.net/addgene:34832) were a gift from Cynthia Kenyon (Department of Biochemistry and Biophysics, UCSF, San Francisco, California, USA) (47). HT115(DE3) bacteria, transformed with each RNAi construct, were seeded on NGM plates containing 1 mM isopropyl-β-D-thiogalactopyranoside (IPTG) and 100 μg/mL carbenicillin (RNAi plates). For each RNAi experiment, a positive control obtained from feeding worms with bacteria carrying the pLT61 vector (Fire Kit, Addgene) was included. This plasmid contains 0.8 kb of *unc-22*, a gene whose silencing causes a visible shaking phenotype, inserted into the pL4440 vector.

RNAi was performed following 2 different protocols. For maternal RNAi treatment, *fer-15(b26)* *II* worms were allowed to grow until L4 on NGM plates seeded with OP50 at 16°C and then transferred to RNAi plates for 2 days at 20°C. Progeny were then transferred at the L4 stage to a new RNAi plate for the beginning of the longevity experiment. For adulthood RNAi treatment, *fer-15(b26)* *II* worms were seeded on NGM plates seeded with OP50 and left to grow until L4/young adult stage. Then animals were transferred to RNAi plates for life span experiments.

Life span determination with mutant lines and RNAi-treated worms was performed as previously reported (48) at 20°C (N2 background) or 25°C [*fer-15(b26) II* background]. Animals were scored every day and counted as dead if they did not move after repeated stimuli with platinum wire, while those that crawled off the plate, had extruded organs, or showed hatched progeny inside the uterus (“bag of worms” or “bagging”), were censured. Bagging was observed only in experiments performed at 20°C in the N2 background. Survival curves, which represent the composite of at least 2 independent experiments performed, were compared using the log-rank test.

### Worm movement analysis

Worms were individually transferred onto NGM plates seeded with *E*. *coli* OP50 bacteria at the L4 stage and maintained at 25°C until the experiment (see graphs/figure legends for details about timing and genotypes analyzed). Spontaneous locomotion was observed and measured for 30 seconds in 3 separate intervals and for each worm the total number of body and head bends, reversals, and duration of stillness periods was calculated as reported previously (49, 50). Analysis was also performed after a harsh-touch stimulus at the tail and worm locomotion was observed and measured 20 or 30 seconds after the stimulus. Body and head bends, reversals, duration of stillness periods, and movement duration until the first stop were measured. In both cases, the experiment was performed at least twice.

### Pharynx pumping assay

L4 *fer-15(b26) II* and *myt-1(pan8) I; fer-15(b26) II* worms were individually transferred onto NGM plates seeded with OP50 bacteria and kept at 25°C until the experiment (see graphs/figure legends for details). The pharyngeal pumping rate was assessed as previously reported (51). Briefly, at least four 10-second videos separated by 20-second intervals were recorded for each worm using a digital camera (Leica IC80HD, 29 frames/second) at ×31.5 magnification and replayed at one-third of the original speed to count the number of worm grinder movements. Finally, pumps per minute (ppm) were calculated for each animal.

### Autophagosome/lysosome analysis

*myt-1(pan8)I* animals were crossed with DA2123 (*adIs2122 [lgg-1p:GFP:lgg-1* + *rol-6(su1006)*]) (52) or MAH215 (*sqIs11* [*lgg-1p:mCherry:GFP:lgg-1* + *rol-6(su1006)*]) worm strains and autophagosomal pool size was evaluated in adult worms compared to control animals of the same age (3-day-old worms or L4/young adult stage, respectively), in basal fed conditions or after 24-hour starvation with agitation at room temperature in M9 buffer. Worms were anesthetized with 10 mM NaN_3_, mounted live on a 2% agarose pad, and *Z*-stack images of the pharynx posterior bulb and body wall muscles were acquired using a Leica TCS SP5 scanning confocal microscope with 0.6 μm slice intervals at ×63 magnification, as previously described (53). Images were manually analyzed by counting GFP:LGG-1–positive puncta in the posterior bulb of the pharynx, while mCherry:LGG-1 puncta (autolysosomes) were quantified in the posterior bulb of the pharynx and in the body wall muscle. At least 2 independent experiments were performed.

### Statistics summary

All data are expressed as mean values ± SEM. The specific test for each panel is reported in the Supporting Data Values Excel (XLS) file. For survival curves, the log-rank (Mantel-Cox) test was used. SPSS and Graphpad Prism 8 were used to calculate mean, median, and χ^2^ values for survival curves between groups. All data of this study were first tested for normality to perform a parametric or nonparametric statistical test. Parametric tests were used only where a normal distribution was assumed. Comparisons between 2 groups were done by 2-tailed Student’s *t* tests. To determine whether there was a significant difference between more than 2 groups, 1-way ANOVA was used. Kruskal-Wallis test was used when the measurement variable did not meet the normality assumption of a 1-way ANOVA. GraphPad Prism 8 was used for all statistical analyses. A *P* value of less than 0.05 was considered statistically significant. In all figures, *P < 0.05, **P < 0.01, ***P < 0.001, ****P < 0.0001.

### Study approval

For human data, written informed consent was obtained from all patients, and the study was approved by the responsible Ethical Committee of Istituto Ortopedico Rizzoli (protocol no. 10823, issued on April 26, 2010, Bologna, Italy).

Animal studies were approved by the Italian Ministero della Salute, Ufficio VI (authorization numbers 1060/2015) and by the Ethics Committee of the University of Padova.

### Data availability

Raw data for this article and the statistical test for each figure panel are provided in the Supporting Data Values Excel (XLS) file, with separate tabs for each applicable figure panel.

The mass spectrometry proteomics data have been deposited in the ProteomeXchange Consortium via the PRIDE (54) partner repository with the data set identifier PXD022180.

## Author contributions

The number of experiments performed by each researcher was the method used for assigning the order of the 2 co–first authors. AFR designed and performed both mammal and *C*. *elegans* experiments, analyzed data, interpret results, designed the figures, and wrote the manuscript. VM performed *C*. *elegans* experiments, generated the C2C12 *Mytho*-KO cell line, analyzed data, interpreted results, and helped write the manuscript. GM, RS, and HBJJ designed and performed some experiments. JVL performed statistical Cox proportional hazards analysis. LP and SM generated the worm KO model by CRISPR/Cas9 technology. JW and PG generated the endogenous MYTHO-HA cells. VB and PG generated the MYTHO-HA–overexpressing stable cell line and performed the mass spectrometry analysis. AA provided muscle lysates of aged mice. ID, VR, and FC helped in interpreting results. Myoatlas figure panels were elaborated from a snRNA-seq project previously published by DPM and COS. Human biopsies were obtained by MC, SS, and CF. ET, LS, and SAT helped with writing the manuscript, interpreting the results, and editing the manuscript. MS conceived the project, planned experiments, interpreted the results, and wrote the manuscript. SAT provided the Cherry-LC3B, LAMP2-Cherry, Golgi-GFP, GFP-WIPI2, ATG16L1-FLAG, GFP-WIPI2b RERE (R108E/R128E) mutant, and GFP-WIPI2b FTTG mutant plasmids.

## Supplementary Material

Supplemental data

Unedited blot and gel images

Supporting data values

## Version 1 06/13/2024

In-Press Preview

## Version 2 08/01/2024

Electronic publication

## References

[1] Leidal AM (2018). Autophagy and the cell biology of age-related disease. Nat Cell Biol.

[2] Kenyon C (1993). A C. elegans mutant that lives twice as long as wild type. Nature.

[3] Johnson TE (1990). Increased life-span of age-1 mutants in Caenorhabditis elegans and lower Gompertz rate of aging. Science.

[4] Ogg S (1997). The Fork head transcription factor DAF-16 transduces insulin-like metabolic and longevity signals in C. elegans. Nature.

[5] Lin K (1997). daf-16: An HNF-3/forkhead family member that can function to double the life-span of Caenorhabditis elegans. Science.

[6] Webb AE, Brunet A (2014). FOXO transcription factors: key regulators of cellular quality control. Trends Biochem Sci.

[7] Martins R (2016). Long live FOXO: unraveling the role of FOXO proteins in aging and longevity. Aging Cell.

[8] Friedman DB, Johnson TE (1988). A mutation in the age-1 gene in Caenorhabditis elegans lengthens life and reduces hermaphrodite fertility. Genetics.

[9] Lopez-Otin C (2013). The hallmarks of aging. Cell.

[10] Carnio S (2014). Autophagy impairment in muscle induces neuromuscular junction degeneration and precocious aging. Cell Rep.

[11] Fernandez AF (2018). Disruption of the beclin 1-BCL2 autophagy regulatory complex promotes longevity in mice. Nature.

[12] Madeo F (2015). Essential role for autophagy in life span extension. J Clin Invest.

[13] Melendez A (2003). Autophagy genes are essential for dauer development and life-span extension in C. elegans. Science.

[14] Mizushima N, Levine B (2020). Autophagy in human diseases. N Engl J Med.

[15] Mizushima N (2020). The ATG conjugation systems in autophagy. Curr Opin Cell Biol.

[16] Sawa-Makarska J (2020). Reconstitution of autophagosome nucleation defines Atg9 vesicles as seeds for membrane formation. Science.

[17] Sebastian D, Zorzano A (2020). Self-eating for muscle fitness: autophagy in the control of energy metabolism. Dev Cell.

[18] Stoeger T (2018). Large-scale investigation of the reasons why potentially important genes are ignored. PLoS Biol.

[19] Milan G (2015). Regulation of autophagy and the ubiquitin-proteasome system by the FoxO transcriptional network during muscle atrophy. Nat Commun.

[20] Petrany MJ (2020). Single-nucleus RNA-seq identifies transcriptional heterogeneity in multinucleated skeletal myofibers. Nat Commun.

[21] Huang C (2004). Measurements of age-related changes of physiological processes that predict lifespan of Caenorhabditis elegans. Proc Natl Acad Sci U S A.

[22] Mizushima N (2004). In vivo analysis of autophagy in response to nutrient starvation using transgenic mice expressing a fluorescent autophagosome marker. Mol Biol Cell.

[23] Fueller J (2020). CRISPR-Cas12a-assisted PCR tagging of mammalian genes. J Cell Biol.

[24] Kojima W (2021). Mammalian BCAS3 and C16orf70 associate with the phagophore assembly site in response to selective and non-selective autophagy. Autophagy.

[25] McKay JP (2004). eat-2 and eat-18 are required for nicotinic neurotransmission in the Caenorhabditis elegans pharynx. Genetics.

[26] Chi C (2016). Nucleotide levels regulate germline proliferation through modulating GLP-1/Notch signaling in C. elegans. Genes Dev.

[27] Berry LW (1997). Germ-line tumor formation caused by activation of glp-1, a Caenorhabditis elegans member of the Notch family of receptors. Development.

[28] Arantes-Oliveira N (2002). Regulation of life-span by germ-line stem cells in Caenorhabditis elegans. Science.

[29] Hansen M (2008). A role for autophagy in the extension of lifespan by dietary restriction in C. elegans. PLoS Genet.

[30] Bakula D (2017). WIPI3 and WIPI4 β-propellers are scaffolds for LKB1-AMPK-TSC signalling circuits in the control of autophagy. Nat Commun.

[31] Huttlin EL (2017). Architecture of the human interactome defines protein communities and disease networks. Nature.

[32] Dooley HC (2014). WIPI2 links LC3 conjugation with PI3P, autophagosome formation, and pathogen clearance by recruiting Atg12-5-16L1. Mol Cell.

[33] Fracchiolla D (2020). A PI3K-WIPI2 positive feedback loop allosterically activates LC3 lipidation in autophagy. J Cell Biol.

[34] Harada K (2019). Two distinct mechanisms target the autophagy-related E3 complex to the pre-autophagosomal structure. Elife.

[35] Nishimura T (2013). FIP200 regulates targeting of Atg16L1 to the isolation membrane. EMBO Rep.

[36] Lystad AH (2019). Distinct functions of ATG16L1 isoforms in membrane binding and LC3B lipidation in autophagy-related processes. Nat Cell Biol.

[37] Dudley LJ (2019). Intrinsic lipid binding activity of ATG16L1 supports efficient membrane anchoring and autophagy. EMBO J.

[38] Jelani M (2019). A mutation in the major autophagy gene, WIPI2, associated with global developmental abnormalities. Brain.

[39] Yoshihara K (2015). The landscape and therapeutic relevance of cancer-associated transcript fusions. Oncogene.

[40] Leduc-Gaudet JP (2023). MYTHO is a novel regulator of skeletal muscle autophagy and integrity. Nat Commun.

[41] Brenner S (1974). The genetics of Caenorhabditis elegans. Genetics.

[42] Paix A (2015). High efficiency, homology-directed genome editing in Caenorhabditis elegans using CRISPR-Cas9 ribonucleoprotein complexes. Genetics.

[43] Hobert O (2002). PCR fusion-based approach to create reporter gene constructs for expression analysis in transgenic C. elegans. Biotechniques.

[44] Wang H (2017). cGAL, a temperature-robust GAL4-UAS system for Caenorhabditis elegans. Nat Methods.

[45] Jacomin AC (2016). iLIR database: a web resource for LIR motif-containing proteins in eukaryotes. Autophagy.

[46] Puntervoll P (2003). ELM server: A new resource for investigating short functional sites in modular eukaryotic proteins. Nucleic Acids Res.

[47] Dillin A (2002). Timing requirements for insulin/IGF-1 signaling in C. elegans. Science.

[48] Hsin H, Kenyon C (1999). Signals from the reproductive system regulate the lifespan of C. elegans. Nature.

[49] Sawin ER (2000). C. elegans locomotory rate is modulated by the environment through a dopaminergic pathway and by experience through a serotonergic pathway. Neuron.

[50] Chiba CM, Rankin CH (1990). A developmental analysis of spontaneous and reflexive reversals in the nematode Caenorhabditis elegans. J Neurobiol.

[51] http://www.wormbook.org/chapters/www_measurepharyngeal/measurepharyngeal.html.

[52] Kang C (2007). Dual roles of autophagy in the survival of Caenorhabditis elegans during starvation. Genes Dev.

[53] Chang JT (2017). Spatiotemporal regulation of autophagy during *Caenorhabditis elegans* aging. Elife.

[54] Perez-Riverol Y (2019). The PRIDE database and related tools and resources in 2019: improving support for quantification data. Nucleic Acids Res.

