# Supplementary material for: C16ORF70/MYTHO promotes healthy aging in C.elegans and prevents cellular senescence in mammals
Source: J Clin Invest. 2024 Jun 13;134(15):e165814. doi: 10.1172/JCI165814 (PMC11291266; doi:10.1172/JCI165814)
Supplement: Supplemental data [file jci-134-165814-s023.pdf]

Supplemental Figures and Methods

*C16ORF70/*Mytho promotes healthy ageing in *C.elegans* and prevents cellular senescence in mammals

Anais Franco-Romero[1,2#], Valeria Morbidoni[3,4#], Giulia Milan[5], Roberta Sartori[1,2], Jesper Wulff[6], Vanina Romanello[1,2], Andrea Armani[1,2], Leonardo Salviati[3,4], Maria Conte[7], Stefano Salvioli[7,8], Claudio Franceschi[9], Viviana Buonomo[10], Casey O. Swoboda[11, 12], Paolo Grumati [10,13], Luca Pannone[14], Simone Martinelli[14], Harold B.J. Jefferies[15], Ivan Dikic[6,16], Jennifer Van der Laan[17,18] , Filipe Cabreiro[17, 18], , Douglas P. Millay[11, 19], Sharon A. Tooze[15], Eva Trevisson[3,4*], Marco Sandri[1,2,20,21,22*]

[1] Department of Biomedical Sciences, University of Padova, via U. Bassi 58b, 35121 Padova, Italy.[2] Veneto Institute of Molecular Medicine, via Orus 2, 35129 Padova, Italy. [3]Clinical Genetics Unit, Department of Women's and Children's Health, University of Padova, via Giustiniani 3, 35128 Padova, Italy. [4] IRP Città della Speranza Corso Stati Uniti 4 35127 Padova, Italy. [5] Department of Cardiac Surgery, University Hospital Basel, Basel, Switzerland; Department of Biomedicine, University of Basel, Basel, Switzerland [6] Institute of Biochemistry II, Goethe University Frankfurt - Medical Faculty, University Hospital, Theodor Stern Kai 7, 60590 Frankfurt am Main, Germany. [7] Department of Medical and Surgical Science (DIMEC), University of Bologna, via San Giacomo 12, 40126 Bologna, Italy.. [8] IRCCS Azienda Ospedaliero-Universitaria di Bologna, via San Giacomo 12, 40126, Bologna, Italy [9]Institute of Information Technologies, Mathematics and Mechanics, Lobachevsky State University, Nizhny Novgorod, Russia [10] Telethon Institute of Genetics and Medicine (TIGEM), via Campi Flegrei 34, 80078 Pozzuoli, Italy. [11] Division of Molecular Cardiovascular Biology, Cincinnati Children's Hospital Medical Center, Cincinnati, OH, USA.[12] Division of Biomedical Informatics, Cincinnati Children's Hospital Medical Center, Cincinnati, OH, 10 USA. [13] Department of Clinical Medicine and Surgery, University of Naples Federico II, via Pansini 5, 80131 Naples, Italy. [14] Department of Oncology and Molecular Medicine, Istituto Superiore di Sanità, Rome, Italy. [15] The Francis Crick Institute, Molecular Cell Biology of Autophagy, 1 Midland Road, London, NW1 1AT, UK nb vu8. [16] Buchmann Institute for Molecular Life Sciences, Goethe University Frankfurt - Riedberg Campus, 60438 Frankfurt am Main, Germany. [17] CECAD Research Cluster, University of Cologne, Joseph-Stelzmann-Straße 26, 50931 Cologne, Germany. [18]Institute of Clinical Sciences, Imperial College London, Hammersmith Hospital Campus, Du Cane Road, London W12 0NN, UK. [19] Department of Pediatrics, University of Cincinnati College of Medicine, Cincinnati, OH, USA. [20] Myology Center, University of Padova, via G. Colombo 3, 35100 Padova, Italy. [21] Department of Medicine, McGill University, Montreal, Canada. [22] Department of Pharmacology & Therapeutics, College of Medicine, University of Florida, USA.

Content:

Supplemental Figures 1-11

Supplemental Methods

References

Supplemental Figure 1

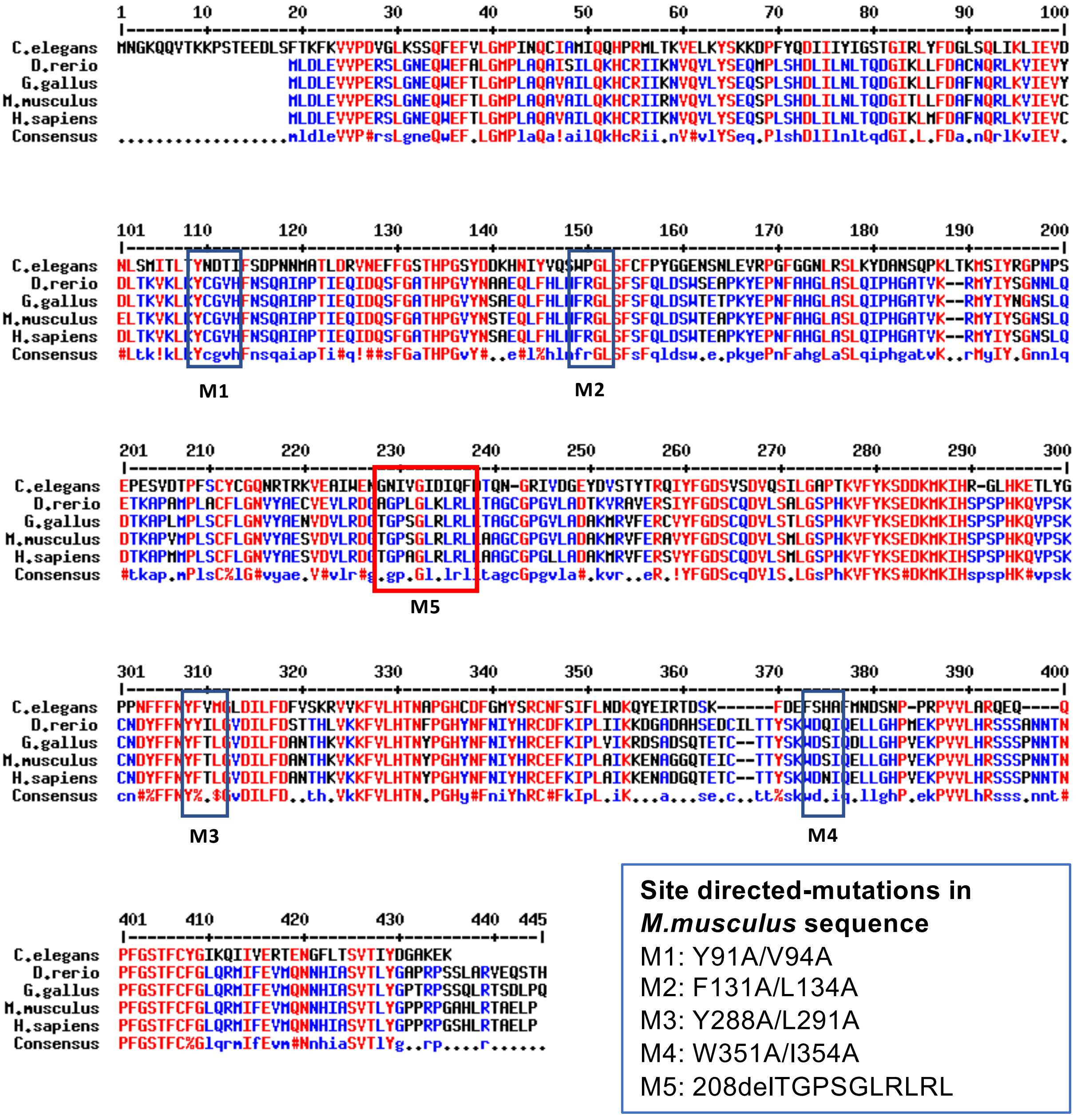

Supplemental Figure 1. BLASTp alignment of MYTHO protein sequences of *C. elegans* (NP_740894), *G. gallus* (NP_001025736), *D. rerio* (NP_001076282), *M. musculus* (NP_663579), *H. sapiens* (NP_001307469). The M1-M5 regions indicated in the sequence alignment are the putative LIR or WD40 domains of MYTHO.

Supplemental Figure 2

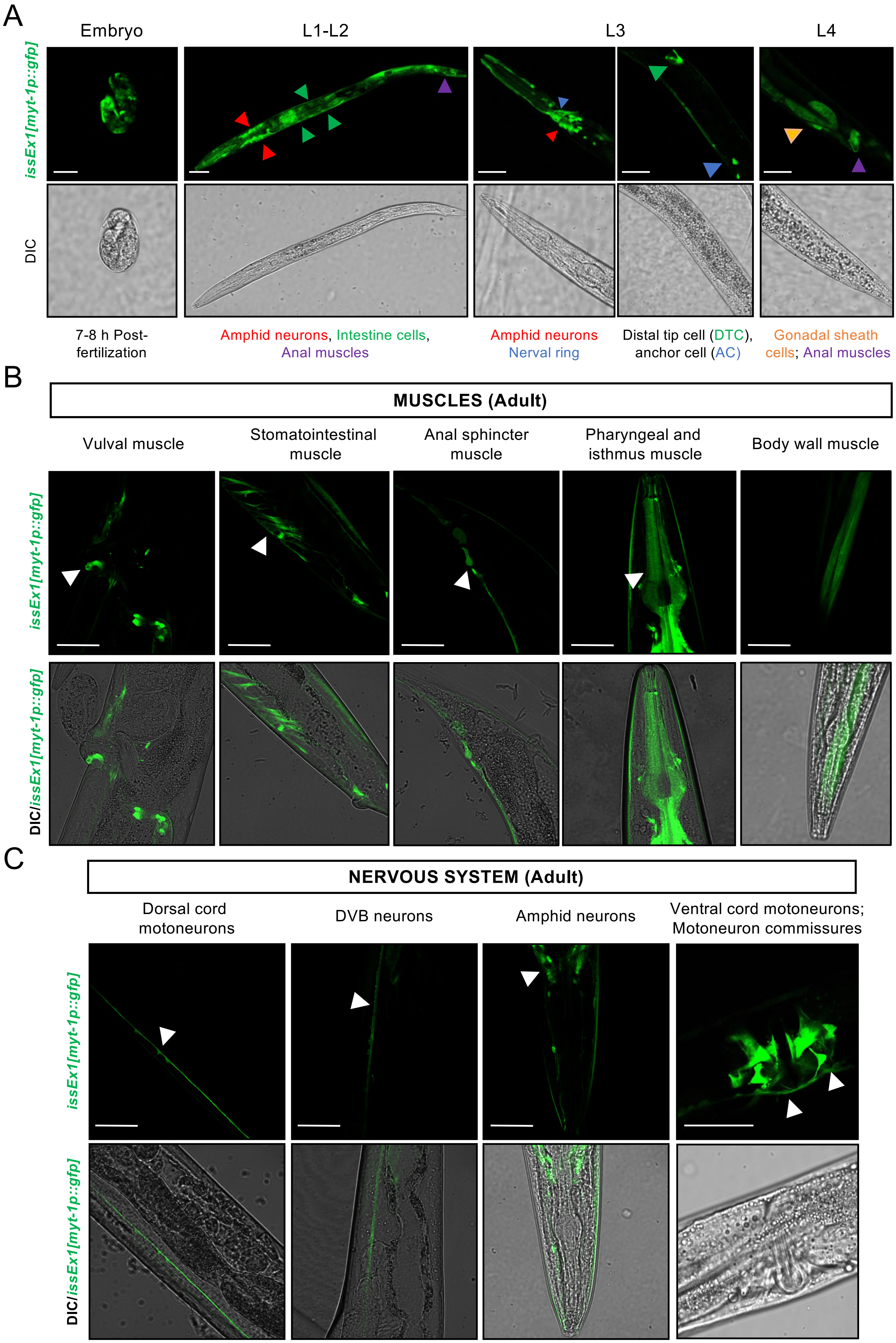

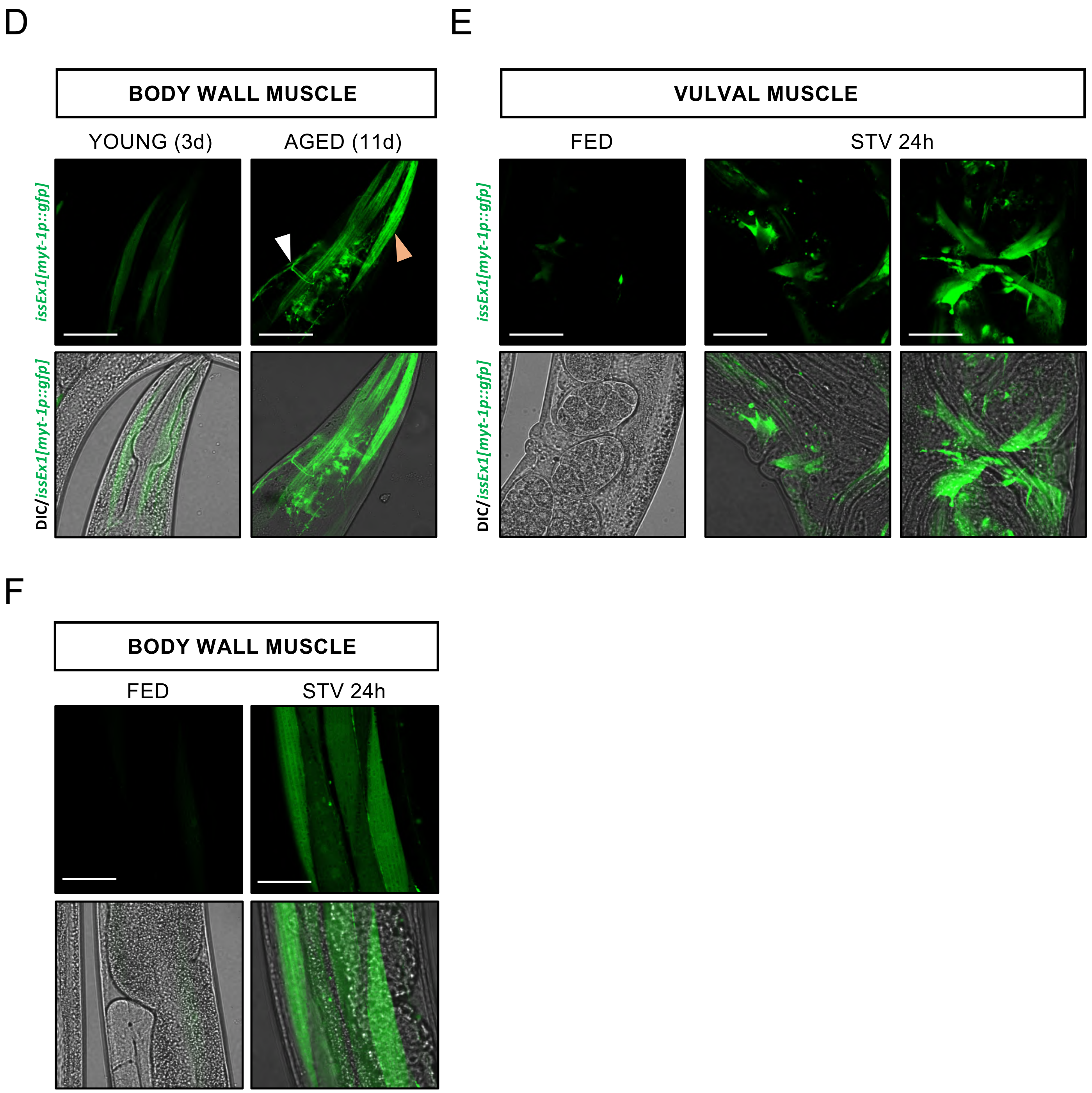

Supplemental Figure 2. *myt-1* expression during development and adulthood in *C. elegans*. (A) Representative fluorescent and DIC images of *issEx1 [myt-1p::gfp]* transgenic worm line during development. *myt-1* expression was observed at 7-8 h post-fertilization (embryo), L1-L2 stage, L3 and L4 stage. Coloured arrowheads indicate cells or organs where *myt-1* is particularly expressed. Scale bar=10µm. **(B)** Representative fluorescent images of *issEx1[myt-1p::gfp]* transgenic worm line and the overlay with DIC showing *myt-1* expression in muscles of adult worms. Arrowheads indicate vulval muscle, stomatointestinal muscle, anal sphincter muscle, pharyngeal and isthmus muscle, body wall muscle. Scale bar=50µm. **(C)** Representative fluorescent images of *issEx1 [myt-1p::gfp]* transgenic worm line and the overlay with DIC showing *myt-1* expression in neurons of adult worms. Arrowheads indicate dorsal cord motoneurons, DVB neurons, amphid neurons, ventral cord motoneurons and motoneuron commissures. Scale bar=50µm. **(D-E)** Representative fluorescent images of *issEx1 [myt-1p::gfp]* transgenic worm line and the overlay with DIC showing a higher *myt-1* expression in vulval muscle **(D)** and body wall muscle **(E)** after 24 h of starvation compared to controls. Scale bar=50µm. **(F)** Representative fluorescent images of *issEx1 [myt-1p::gfp]* transgenic worm line and overlay with DIC showing a higher *myt-1* expression in aging (3-day-old vs 11-day-old worms). The white arrowhead points at neurons and the orange one shows muscles. Scale bar=50µm.

Supplemental Figure 3

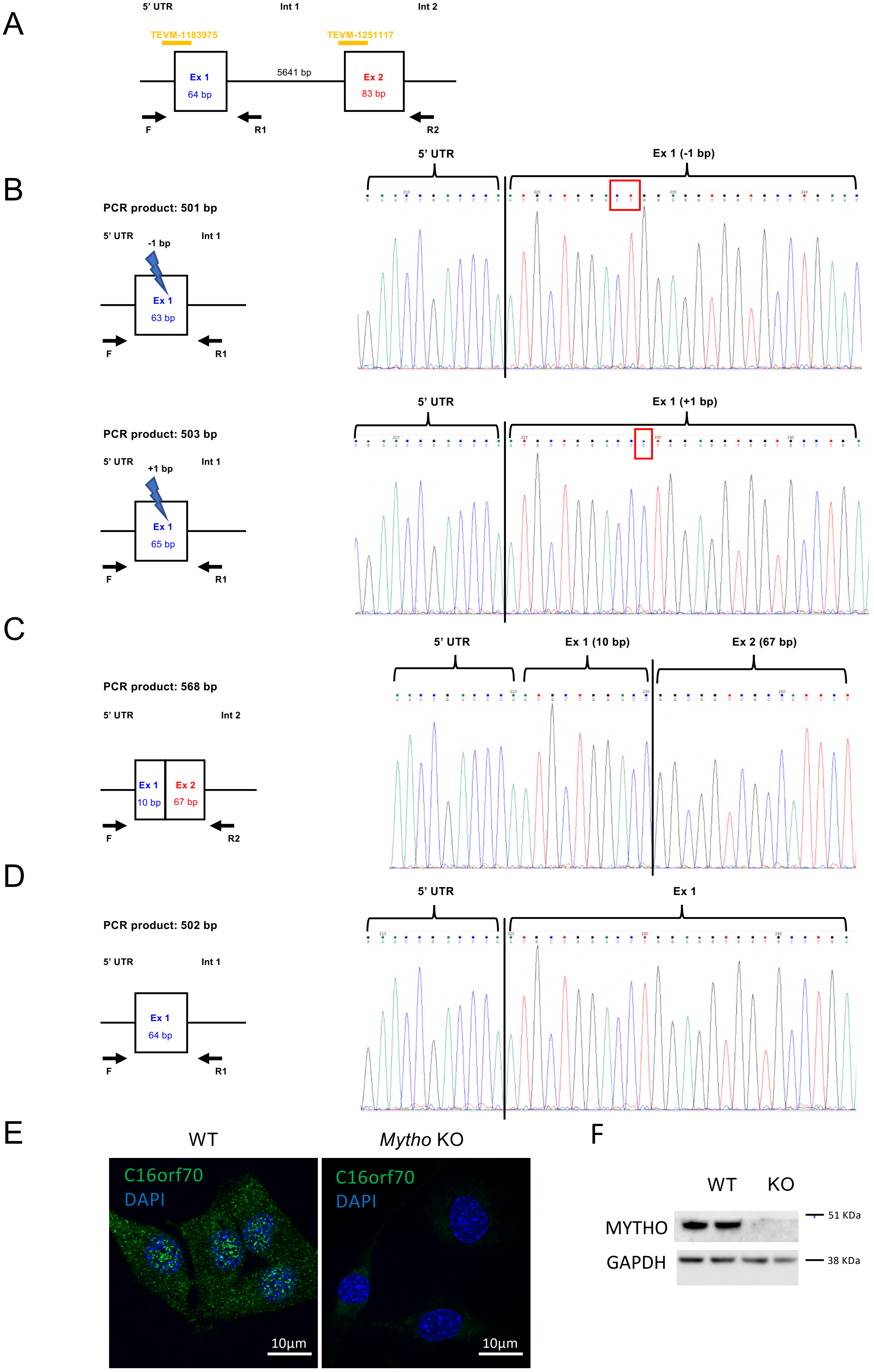

Supplemental Figure 3. Generation of a *Mytho* KO C2C12 cell line **(A)** A schematic representation of the 5' portion of murine *Mytho* gene highlighting the two different CRISPR target sequences (TEVM-1183975 and TEVM-1251117, Transomic Technology) targeting exon 1 and 2 of *Mytho*, respectively. The arrows indicate forward (F) and reverse (R1 and R2) primers used for PCR genotyping. **(B)** Examples of isolated cell clones harboring frameshift mutations: deletion of 1 base pair (above) or insertion of 1 base pair (below) in exon 1. **(C)** Example of a cell clone with a big genomic deletion between the target sequences of exon 1 and 2**. (D)** A cell clone obtained transfecting cells with a non-targeting control gRNA (TELA1011, Transomic Technology). In b), c) and d) a schematic representation of genome editing outcome is depicted on the left, while Sanger sequencing of the PCR reaction performed for clone genotyping is shown on the right. **(E)** Abcam c16orf70 antibody specificity using WT and *Mytho* KO cell line. Puncta signal in the cytoplasm is specific. Nucleus dots are sometimes present in the *Mytho* KO cell line suggesting an unspecific signal. **(F)** Example of immunoblot showing the total depletion of Mytho protein in *Mytho* KO C2C12 cell line.

Supplemental Figure 4

A

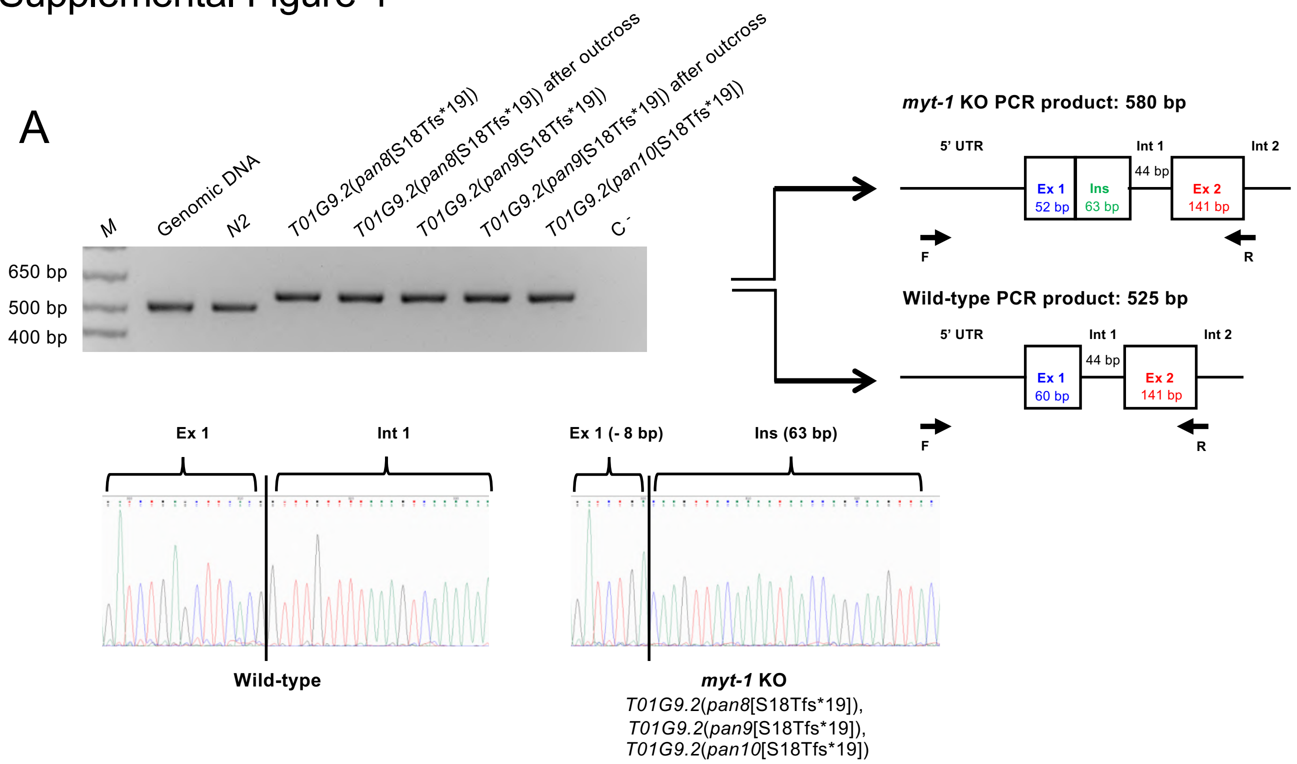

B

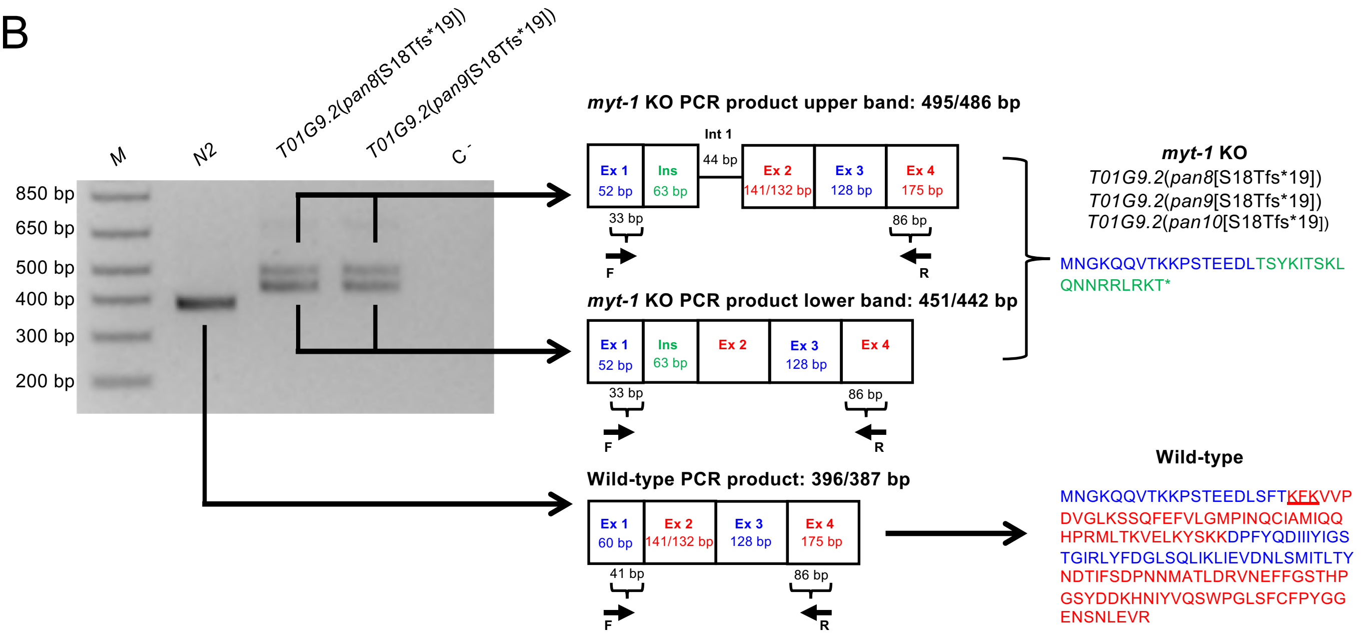

C

Strains generated for this study	Number of analysed lines	Backcrosses
myt-1(pan8) I	1	2
myt-1(pan9) I	1	2
myt-1(pan8) I; fer-15(b26) II	1	2
myt-1(pan9) I; fer-15(b26) II	2	3
myt-1(pan8) I; adIs2122 [lgg-1p::GFP::lgg-1 + rol-6(su1006)]	2	2
myt-1(pan8) I; sqIs11 [lgg-1p::mCherry::GFP::lgg-1 + rol-6]	2	3
myt-1 (pan8) I; fer-15(b26) II; daf-2 (e1370) III	1	3
myt-1(pan8) I; daf-16 (mu86) I; fer-15(b26) II	1	3
myt-1(pan8) I; fer-15(b26) II; glp-1(e2141) III	2	3
myt-1(pan8) I; fer-15(b26) II ; eat-2(ad1116) II	2	3
issEx1[myt-1p::gfp]	3	-
oxTi10882; syIs321	1	1
fer-15(b26) II; oxTi10882; syIs321 (OE myt-1)	2	2

D

Sequence	Off-target score (IDT) *	CFD specificity score (CRISPOR) °
TGAAGAAGATCTGAGCTTCA	96	100

* A higher value of the off-target score (0-100) indicates lower off-target risk (**IDT Technologies**)
° The higher the specificity score, the lower are off-target effects in the genome (**CFD**, cutting frequency determination, from Doench *et al*, 2016)

E

Sequence	PAM	# MM *	Gene (location and DSB site)	Locus (chr, strand and position)	Score (IDT) °	CFD Off-target score (CRISPOR) §
TGAAGAAGATCTGAGCTTCA	CGG		*T01G9.2* (exon)	I: -8288050		
TGTGGAAGATC-GAGCTTCA	AGG	3; 1	*fbxc-29* (exon)	II: +916768	26	
AGAGGAACATCTGAGTTTCA	AAG	4; 1	*hli-8* (intron 1; +118 bp from exon 1)	I: -5469429	27	
TGAACAAAATCTTGAGCTTCA	AGG	3; 1	*F28C10.3* (intron 3; -126 bp from exon 2)	X: -534888	30	
TGAAGAAAATCTGAAATTCA	AAG	3; 1	*F17C11.11* (exon)	V: -10938433	38	
AGGAGAAGATCTGCGCTTCA	AGG	3; 1	*lin-18* (exon)	X: +3959273	48	0.130952
CGAAGAAGAT--GAGCTTCA	AAG	3; 2	*C30B5.6* (exon)	II: -6222435	52	
CAAAGAAAATCTGAGCTTGA	GAG	4; 2	-	X: +13179664	52	
TGAAGAAGACATGAGTTTCA	CAG	3; 3	*bath-45* (exon)	II: -13123305	60	
TGGAGAATTTCTGAGTTTCA	GAG	4; 2	-	IV: -9110898	61	
TGAAGAAGATCCCAAATTCA	GAG	4; 4	*C38C3.8* (pseudogene)	V: -1503168	75	
TAAAGAACATCTCATCTTCA	AAG	4; 2	*fbxb-56* (3' UTR, 145 bp from exon 2)	I: +14085501	84	
AGAAGTATATCTGATCTTCA	GAG	4; 1	*C18H9.13* (pseudogene)	II: +6679543	84	
TGAAGAAAATCTGAAATTAA	AAG	4; 3	-	II: +10091576	84	
GGAAGAAGCTTTGAGCTCCA	CAG	4; 3	-	I: -7033158	87	
TGAAGAATATTTGGGATTCA	TGG	4; 3	*T28H10.5* (exon)	V: +12506380	91	0.089744
TGAAGTAGATCCTAGATTCA	CAG	4; 3	R03D7.8 (intron 7; +19 bp from exon 7)	II: +10954817	96	
TGAAGAAGTTC-GAGCTGCA	GAG	3; 3	*fbxc-30* (exon)	II: +918075	98	
TGGAGAAGCTCGGAGGTTCA	GAG	4; 3	*cdh-12* (exon)	III:-1172657	101	
TGAAGAAGAAAAGATCTTCA	AAG	4; 4	*R09H10.2* (5' UTR, 377 bp from exon 1)	IV: -10604895	116	
TGAAGAAGAGCTGAGCACAA	AAG	4; 4	*syd-2* (exon)	X: -10554163	152	

* Total and seed region-specific mismatches in the guide RNA compared to *T01G9.2* guide.
° A lower score indicates higher potential editing.
§ The higher the CFD score, the more likely there is off-target cleavage at that site.

Supplemental Figure 4. (A) Genotyping of *myt-1* KO strains. On the left, an illustrative agarose gel image showing genotyping of strains *T01G9.2(pan8[S18Tfs*19])* and *T01G9.2(pan9[S18Tfs*19])*, before and after outcrosses, and *T01G9.2(pan10[S18Tfs*19])*. M: 1 kb plus molecular marker (Thermo Fisher). In the manuscript these strains are referred to as: *myt-1(pan8) I* and *myt-1(pan9) I*. On the right, a schematic representation of the 5' portion of *myt-1* gene showing the predictive genome editing outcome. In particular, exon 1 loses the final 8 base pairs (bp) with the concomitant insertion (Ins) of 63 nucleotides (nt) deriving from the *T01G9.2* KO ssODN (single-stranded oligodeoxynucleotide). The arrows indicate forward (F) and reverse (R) primers used for PCR genotyping. The resulting *myt-1* KO PCR product is 580 bp, compared to the 525 bp long wild-type. Below, Sanger sequencing of *myt-1* wild-type and KO PCR products. On the left, the wild-type *myt-1* sequence pointing out exon1 - intron1 junction, while on the right, the same gene portion harboring the 63 nt insertion in *myt-1* KO strains. **(B)** RT-PCR analysis of *myt-1* KO strains. After RNA extraction and cDNA synthesis, a PCR reaction was performed using forward (F) and reverse (R) primers designed on exon 1 and 4 of *myt-1*, respectively. PCR products were run on a 2% agarose gel (shown on the left) and bands were then excised from gel and sequenced. The results are depicted on the right and as highlighted in the scheme, both isoforms of *myt-1* transcript, differing of the first nine nucleotides of exon 2, are present in total worm RNA extracts. The resulting aminoacidic sequences are shown next: the predicted protein sequence in *T01G9.2(pan8[S18Tfs*19])*, *T01G9.2(pan9[S18Tfs*19])*, and *T01G9.2(pan10[S18Tfs*19])* strains displays a stop codon after 35 amino acids; the wild-type MYT-1 protein (the N-terminal portion up to amino acid 168), carrying or not amino acids KFK (position 21-23) is shown below. **(C)** Table showing all strains generated for this article. **(D)** The specificity of the *myt-1* guideRNA used to generate *myt-1* KO strains by CRISPR/Cas9 technology was evaluated by on-line predictive tools: the off-target score from IDT technologies website

(https://eu.idtdna.com/site/order/designtool/index/CRISPR_SEQUENCE) and the CFD specificity score predicted with the CRISPOR website (http://crispor.gi.ucsc.edu/) are shown in the table. **(E)** Table of off-target sites for the *myt-1* guideRNA predicted by IDT technologies and CRISPOR websites. In each off-target sequence, mismatches compared to the *myt-1* guideRNA sequence are shown in red, while insertions are shown in yellow. The presence of NGG, NGA and NAG as PAM sequences, the number of mismatches (total and in the seed region, which is critical for the guideRNA specificity), the predicted location of double break site and the predicted score were evaluated and potential candidates were excluded by Sanger sequencing.

Supplemental Figure 5

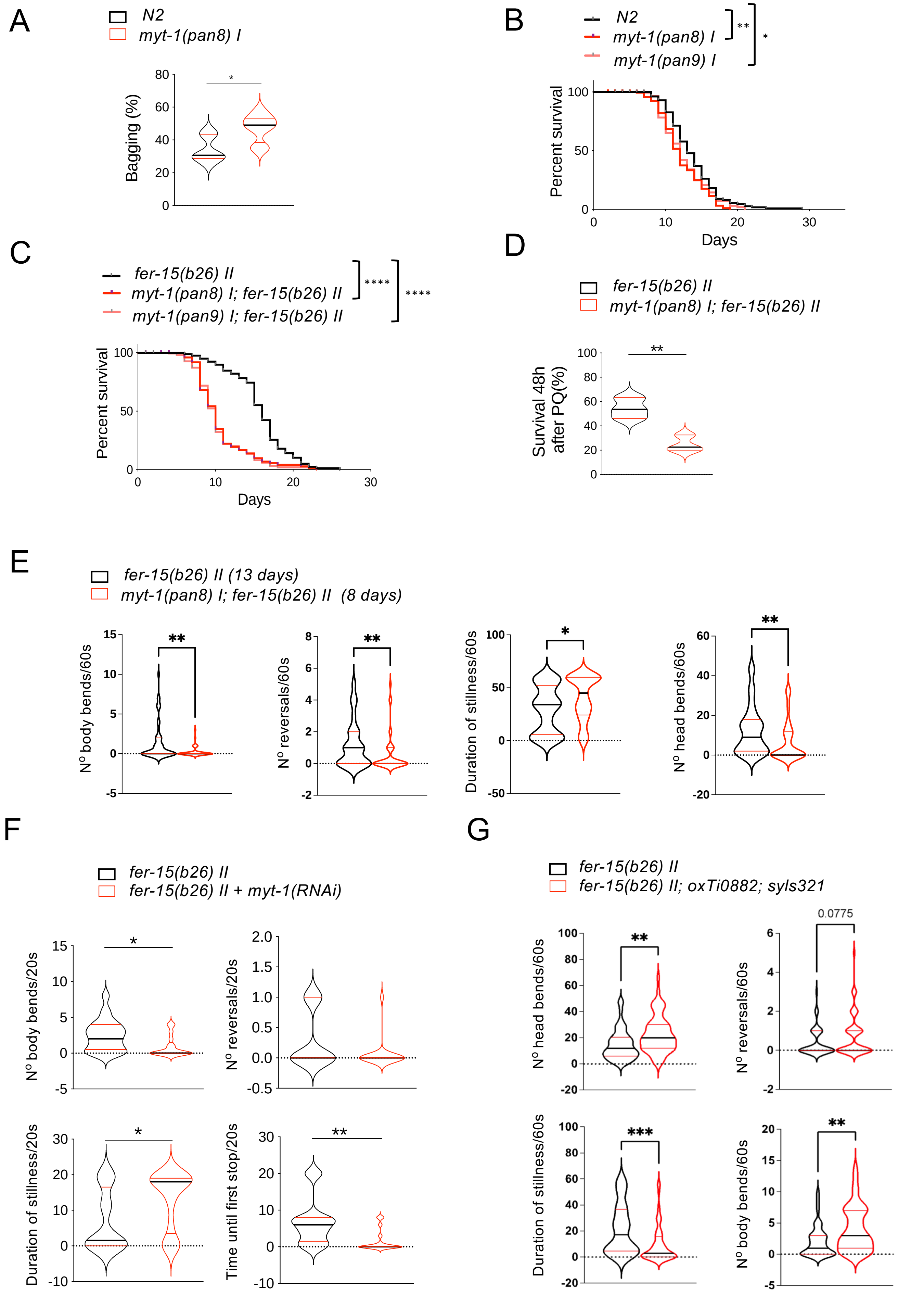

Supplemental Figure 5. (A) Quantification of larvae hatching inside worms (worm bagging) in N2 and *myt-1(pan8) I*, *myt-1(pan9) I* animals. Six independent experiments (>80 worms / experiment) were performed. **(B)** The survival curve of N2 (n=320) and *myt-1(pan8) I*, *myt-1(pan9) I* worms (n=200) incubated at 20 °C is plotted as the average for two independent experiments. Bag-of-worms were censored. **(C)** Survival curve of *fer-15(b26) II* (n=81), *myt-1(pan8) I; fer-15(b26) II*

(n=81) and *myt-1(pan9) I; fer-15(b26) II* (n=160, 2 lines analysed) worms incubated at 25 °C. **(D)** 5-day-old *fer-15(b26) II* (n=95) and *myt-1(pan8) I; fer-15(b26) II* (n=83) worms were incubated with 125 mM paraquat (PQ) for 1 hour at 25 °C and survival fractions were calculated 48 h later. The graph represents the mean of two independent experiments. **(E)** Total number of body and head bends, reversals and duration of stillness periods per minute were calculated at mean lifespan (13 days for *fer-15(b26)* and 8 days for *myt-1(pan8) I; fer-15(b26) II* worms). Two independent experiments were performed. *fer-15(b26) II* (n=50) *myt-1(pan8) I; fer-15(b26) II* (n=40). *(***F)** Movement analysis after a harsh stimulus at the tail of 11-day-old *fer-15(b26)* animals feeded with HT115(DE3) bacteria carrying either the pL4440 empty vector or the pL4440 vector with *myt-1* coding sequence (*myt-1(RNAi*), using a maternal RNAi protocol. Number of body bends, number of reversals, duration of stillness periods and movement duration until the first stop after touch were analysed. *fer-15(b26)II* (n=21), *fer-15(b26) II* + *myt-1(RNAi)* (n=17). **(G)** Movement analysis after a harsh stimulus at the tail of 14-day-old *fer-15(b26) II* worms (n=49) or 14-day-old *fer-15(b26) II; oxTi10882; syIs321* (n=62). Total number of body and head bends, reversals, and duration of stillness periods per minute were calculated. Data in A, D, E, F, G were analysed with Student's *t*-test, *p <0.05, **p <0.01, ***p <0.001. Data in B and C were analysed using the log-rank test. ****p<0.0001 (See figure S6A for lifespan experiments details and statistics).

Supplemental Figure 6

A

	Mean survival (days)	Mean life extension (%)	Median survival (days)	Median life extension (%)	*p* value (log rank)	Chi square	Events/n	N	Events/n (each repl)	Mean and median survival (days, each repl)	*p* value and Chi square (each repl)	Temp (°C)	GRAPH
N2	13.68		13				113/160	2	1) 56/80<br>2) 57/80	1) 13.12, 12<br>2) 14.24, 14	1) <0.01, 6.70 (*pan8* vs WT)<br>2) 0.191, 1.71 (*pan8* vs WT)<br>1) 0.079, 3.08 (*pan9* vs WT)<br>2) 0.05, 3.84 (*pan9* vs WT)	20 °C	Fig.S5b
myt-1(pan8) I	12.19	-10.9 (vs WT)	12	-7.7 (vs WT)	<0.01 (vs WT)	9.92 (vs WT)	102/200	2	1) 57/100<br>2) 45/100	1) 11.50, 11<br>2) 13.00, 13			
myt-1(pan9) I	12.51	-8.6 (vs WT)	12	-7.7 (vs WT)	<0.05 (vs WT)	5.04 (vs WT)	103/200	2	1) 52/100<br>2) 51/100	1) 11.92, 10<br>2) 12.98, 13			
fer-15(b26) II	15.70		16				78/81	1				25°C from L4	Fig.S5c
myt-1(pan8) I; fer-15(b26) II	10.56	-32.7 (vs WT)	10	-38.0 (vs WT)	<0.0001 (vs WT)	43.88 (vs WT)	72/81	1					
myt-1(pan9) I; fer-15(b26) II	10.33	-34.2 (vs WT)	10	-38.0 (vs WT)	<0.0001 (vs WT)	66 (vs WT)	149/160	1					
fer-15(b26) II	13.00	-30.2	13	-38.5	<0.0001	236.80	485/521	5	1) 132/150<br>2) 142/150<br>3) 72/81<br>4) 80/80<br>5) 59/60	1) 14.09, 15<br>2) 13.62, 14<br>3) 13.76, 14<br>4) 11.63, 12<br>5) 9.90, 10	1) <0.0001, 64.42<br>2) <0.0001, 104.14<br>3) <0.0001, 41.73<br>4) <0.0001, 30.85<br>5) 0.0002, 14.36	25°C from L4	Fig 4A
myt-1(pan8) I; fer-15(b26) II	9.07		8				461/521	5	1) 123/150<br>2) 128/151<br>3) 75/80<br>4) 79/80<br>5) 56/60	1) 10.06, 9<br>2) 8.80, 8<br>3) 9.31, 8<br>4) 8.56, 8<br>5) 7.89, 7			
fer-15(b26) II	10.92	+154.0	11	+145.5	<0.0001	311.50	139/140	2	1) 80/80<br>2) 59/60	1) 11.63, 12<br>2) 9.90, 10	1) <0.0001, 174.56<br>2) <0.0001, 132.40	25°C from L4	Fig 8A
fer-15(b26) II; daf-2(e1370) III	27.74		27				140/140	2	1) 80/80<br>2) 60/60	1) 26.35, 26<br>2) 29.58, 30			
myt-1(pan8) I; fer-15(b26) II	8.28	+231.9	7	+300.0	<0.0001	397.40	135/140	2	1) 79/80<br>2) 56/60	1) 8.56, 8<br>2) 7.89, 7	1) <0.0001, 222.64<br>2) <0.0001, 172.9		
myt-1(pan8) I; fer-15(b26) II; daf-2(e1370) III	27.48		28				174/180	2	1) 95/100<br>2) 79/80	1) 25.75, 25<br>2) 29.56, 29			
fer-15(b26) II	12.70	-29.4	13	-38.5	<0.0001	87.82	204/216	3	1) 78/81<br>2) 49/54<br>3) 77/81	1) 12.01, 12<br>2) 12.19, 12<br>3) 13.61, 14	1) <0.0001, 36.44<br>2) <0.0001, 33.22<br>3) <0.0001, 22.15	25°C from L4	Fig 8B
myt-1(pan8) I; fer-15(b26) II	8.97		8				218/244	3	1) 71/81<br>2) 74/81<br>3) 73/82	1) 8.42, 8<br>2) 8.14, 8<br>3) 10.34, 9			
fer-15(b26) II; eat-2(ad1116) II	14.33	-9.8	14	-7.1	<0.05	4.53	130/171	2	1) 60/81<br>3) 70/90	1) 13.84, 14<br>3) 14.73, 14	1) 0.25, 1.33<br>3) 0.21, 1.56		
myt-1(pan8) I; fer-15(b26) II; eat-2(ad1116) II	12.92		13				168/263	3	1) 56/81<br>2) 52/81<br>3) 60/101	1) 12.39, 13<br>2) 12.33, 12<br>3) 13.82, 14			
fer-15(b26) II	12.13	+22.4	12	+25.0	<0.0001	41.09	127/135	2	1) 78/81<br>2) 49/54	1) 12.10,12<br>2) 12.19, 12	1) <0.0001, 23.80<br>2) <0.0001, 17.10	25°C from L4	Fig 8C
fer-15(b26) II; glp-1(e2141) III	14.85		15				157/163	2	1) 76/81<br>2) 81/82	1) 14.25, 15<br>2) 15.46, 15			
myt-1(pan8) I; fer-15(b26) II	8.27	-1.0	8	0.0	0.6694	0.18	145/162	2	1) 71/81<br>2) 74/81	1) 8.42, 8<br>2) 8.14, 8	1) 0.045, 4.01<br>2) 0.456, 0.56		
myt-1(pan8) I; fer-15(b26) II; glp-1(e2141) III	8.19		8				130/162	2	1) 64/81<br>2) 66/81	1) 7.58, 7<br>2) 8.75, 8			
fer-15(b26) II	14.37		15				158/162	3	1) 30/31<br>2) 47/50<br>3) 81/81	1) 13.16, 14<br>2) 15.36, 16<br>3) 14.25, 15	1) 0.6911,0.16 (line A vs WT)<br>2) 0.08, 3.00 (line A vs WT)<br>3) 0.0011, 10.58 (line A vs WT)	25°C from L4	NOT shown
fer-15(b26) II; oxTi10882; syIs321 line A	13.33	-7.2 (vs WT)	14	-6.7	0.0055 (vs WT)	7.71 (vs WT)	215/226	3	1) 55/62<br>2) 72/76<br>3) 88/88	1) 13.27, 15<br>2) 13.89, 15<br>3) 12.92, 13	1) 0.76, 0.09 (line B vs WT)<br>2) 0.0026, 9.08 (line B vs WT)<br>3) 0.0021, 9.44 (line B vs WT)		
fer-15(b26) II; oxTi10882; syIs321 line B	12.56	-12.6 (vs WT)	12	-20	<0.0001 (vs WT)	15.47 (vs WT)	229/259	3	1) 76/96<br>2) 63/72<br>3) 89/90	1) 12.13, 10.5<br>2) 11.52, 10<br>3) 12.91, 13			

#For more information please check Excel file with Supporting Data Values

B

	Mean survival (days)	Mean life extension (%)	Median survival (days)	Median life extension (%)	*p* value (log rank)	Chi square	Events/n	N	Events/n (each repl)	Mean and median survival (days, each repl)	*p* value and Chi square (each repl)	RNAi protocol	Temp (^{o}C)	GRAPH
fer-15(b26) II	13.66	-12.0	14	-14.3	<0.0001	42.02	231/252	3	1) 94/97<br>2) 52/63<br>3) 85/92	1) 14.28, 14<br>2) 13.28, 13<br>3) 13.22, 13	1) <0.0001, 40.04<br>2) <0.001, 10.91<br>3) 0.126, 2.34	Maternal	2nd generation at 25 ^{o}C from L4	Fig 4B
fer-15(b26) II + myt-1(RNAi)	12.02		12				244/253	3	1) 65/65<br>2) 93/96<br>3) 86/92	1) 11.46, 12<br>2) 11.93, 12<br>3) 12.53, 12				
fer-15(b26) II	13.02	-17.0	13	-15.4	<0.0001	40.04	240/260	3	1) 58/61<br>2) 83/90<br>3) 99/109	1) 11.91, 12<br>2) 14.01, 15<br>3) 12.83, 13	1) 0.063, 3.45<br>2) 0.0001, 14.75<br>3) <0.001, 26.04	Adulthood	Switched to 25 ^{o}C in RNAi plates at young adult stage	Fig 8D
myt-1(pan8) I; fer-15(b26) II	10.81		11				250/290	3	1) 79/91<br>2) 79/90<br>3) 92/109	1) 10.23, 10<br>2) 11.74, 12<br>3) 10.50, 10				
fer-15(b26) II + atg-18(RNAi)	10.81	-8.5	11	-9.1	<0.0001	19.75	286/313	3	1) 77/88<br>2) 81/90<br>3) 128/135	1) 11.18, 11<br>2) 11.79, 12<br>3) 10.10, 10	1) 0.004, 8.42<br>2) 0.314, 1.01<br>3) <0.0001, 18.81			
myt-1(pan8) I; fer-15(b26) II+ atg-18(RNAi)	9.89		10				280/340	3	1) 68/88<br>2) 79/90<br>3) 133/162	1) 9.84, 9<br>2) 11.35, 12<br>3) 9.04, 10				
fer-15(b26) II	13.02	-17.0	13	-15.4	<0.0001	40.04	240/260	3	1) 58/61<br>2) 83/90<br>3) 99/109	1) 11.91, 12<br>2) 14.01, 15<br>3) 12.83, 13	1) 0.063, 3.45<br>2) 0.0001, 14.75<br>3) <0.0001, 26.04	Adulthood	Switched to 25 ^{o}C in RNAi plates at young adult stage	Fig 8E
myt-1(pan8) I; fer-15(b26) II	10.81		11				250/290	3	1) 79/91<br>2) 79/90<br>3) 92/109	1) 10.23, 10<br>2) 11.74, 12<br>3) 10.50, 10				
fer-15(b26) II + bec-1(RNAi)	14.03	-23.4	14	-28.6	<0.0001	75.77	200/232	3	1) 72/88<br>2) 77/90<br>3) 51/54	1) 12.79, 13<br>2) 14.95, 14<br>3) 14.35,14	1) 0.006, 7.65<br>2) <0.0001, 35.24<br>3) <0.0001, 37.98			
myt-1(pan8) I; fer-15(b26) II+ bec-1(RNAi)	10.75		10				181/228	3	1) 42/57<br>2) 70/90<br>3) 69/81	1) 10.95, 10<br>2) 10.86, 10<br>3) 10.49, 11				
fer-15(b26) II	13.41	+64.3	14	+57.1	<0.0001	285.7	178/187	3	1) 34/36<br>2) 66/70<br>3) 78/81	1) 12.88, 13<br>2) 13.76, 14<br>3) 13.35, 13	1) <0.0001, 64.70<br>2) <0.0001, 88.97<br>3) <0.0001, 140.25	Adulthood	Switched to 25 ^{o}C in RNAi plates at L4	Fig S11A
fer-15(b26) II + daf-2(RNAi)	22.03		22				209/220	3	1) 64/69<br>2) 68/70<br>3) 77/81	1) 19.93, 20<br>2) 22.16, 22<br>3) 23.66, 24				
myt-1(pan8) I; fer-15(b26) II	10.38	+53.9	10	+50	<0.0001	101.8	185/224	3	1) 66/69<br>2) 40/71<br>3) 79/84	1) 10.88, 10<br>2) 11.90, 11<br>3) 9.02, 9	1) <0.0001, 33.04<br>2) 0.007, 7.32<br>3) <0.0001, 82.30			
myt-1(pan8) I; fer-15(b26) II + daf-2(RNAi)	15.97		15				161/186	3	1) 62/70<br>2) 35/35<br>3) 64/81	1) 16.73, 16<br>2) 15.31, 15<br>3) 15.37, 14				
fer-15(b26) II	12.83	+15.7	13	+7.7	<0.0001	29.51	179/195	3	1) 67/70<br>2) 65/71<br>3) 47/54	1) 12.21, 12<br>2) 13.59, 14<br>3) 12.61, 13	1) 0.007, 7.35<br>2) 0.218, 1.52<br>3) <0.0001, 33.29	Maternal	2nd generation at 25 ^{o}C from L4	Fig S11B
fer-15(b26) II + glp-1(RNAi)	14.84		14				204/223	3	1) 61/70<br>2) 63/72<br>3) 80/81	1) 13.87, 14<br>2) 14.65, 14<br>3) 15.74, 16				
myt-1(pan8) I; fer-15(b26) II	11.56	+4.5	12	0.0	0.073	3.22	180/223	3	1) 60/70<br>2) 63/71<br>3) 57/82	1) 10.76, 11<br>2) 11.87, 12<br>3) 12.05, 13	1) 0.146, 2.12<br>2) 0.207, 1.59<br>3) 0.661, 0.192			
myt-1(pan8) I; fer-15(b26) II + glp-1(RNAi)	12.08		12				204/223	3	1) 66/71<br>2) 59/70<br>3) 79/82	1) 11.58, 12<br>2) 12.44, 13<br>3) 12.24, 12				
fer-15(b26) II	12.93	+20.7	13	+15.4	<0.0001	61.40	223/252	3	1) 66/72<br>2) 58/71<br>3) 99/109	1) 12.15, 13<br>2) 14.00, 14<br>3) 12.83, 13	1) <0.0001, 23.21<br>2) <0.0001, 28.01<br>3) 0.002, 9.44	Adulthood	Switched to 25 ^{o}C in RNAi plates at young adult stage	Fig S11D
fer-15(b26) II + bec-1(RNAi)	15.61		15				177/195	3	1) 61/70<br>2) 65/71<br>3) 51/54	1) 14.91, 14<br>2) 17.25, 18<br>3) 14.35, 14				
fer-15(b26) II; eat-2(ad1116) II	14.96	-5.4	15	0.0	<0.0001	16.13	172/232	3	1) 49/71<br>2) 61/71<br>3) 62/90	1) 14.86, 15<br>2) 15.65, 16<br>3) 14.21, 14	1) 0.125, 2.35<br>2) 0.002, 9.26<br>3) 0.014, 5.99			
fer-15(b26) II; eat-2(ad1116) II + bec-1(RNAi)	14.16		15				133/186	3	1) 40/70<br>2) 59/71<br>3) 34/45	1) 14.16, 15<br>2) 14.86, 15<br>3) 12.97, 13				

#For more information please check Excel file with Supporting Data Values

Supplemental Figure 6 (A-B). Tables with the statistics (log-rank test) of lifespan graphs shown in fig. 4A, 8A, 8B, 8C, S5B and S5C for experiments with mutant lines (**A**) or in fig. 4B, 8D, 8E, S11A, S11B and S11D for RNAi experiments **(B).** For each experiment and for each replicate (repl) of the same experiment mean and median survival, *p* value, chi square, amount of deaths (events)/total number of worms (n) were indicated. Moreover, number of replicates performed for each experiment (N), calculation of mean and median life extension/decrease percentage, RNAi protocol, temperature and reference graph are specified. NGM medium and OP50 bacteria were used in A, while NGM supplemented with carbenicillin and IPTG and HT115(DE3) bacteria were used in B.

Supplemental Figure 7

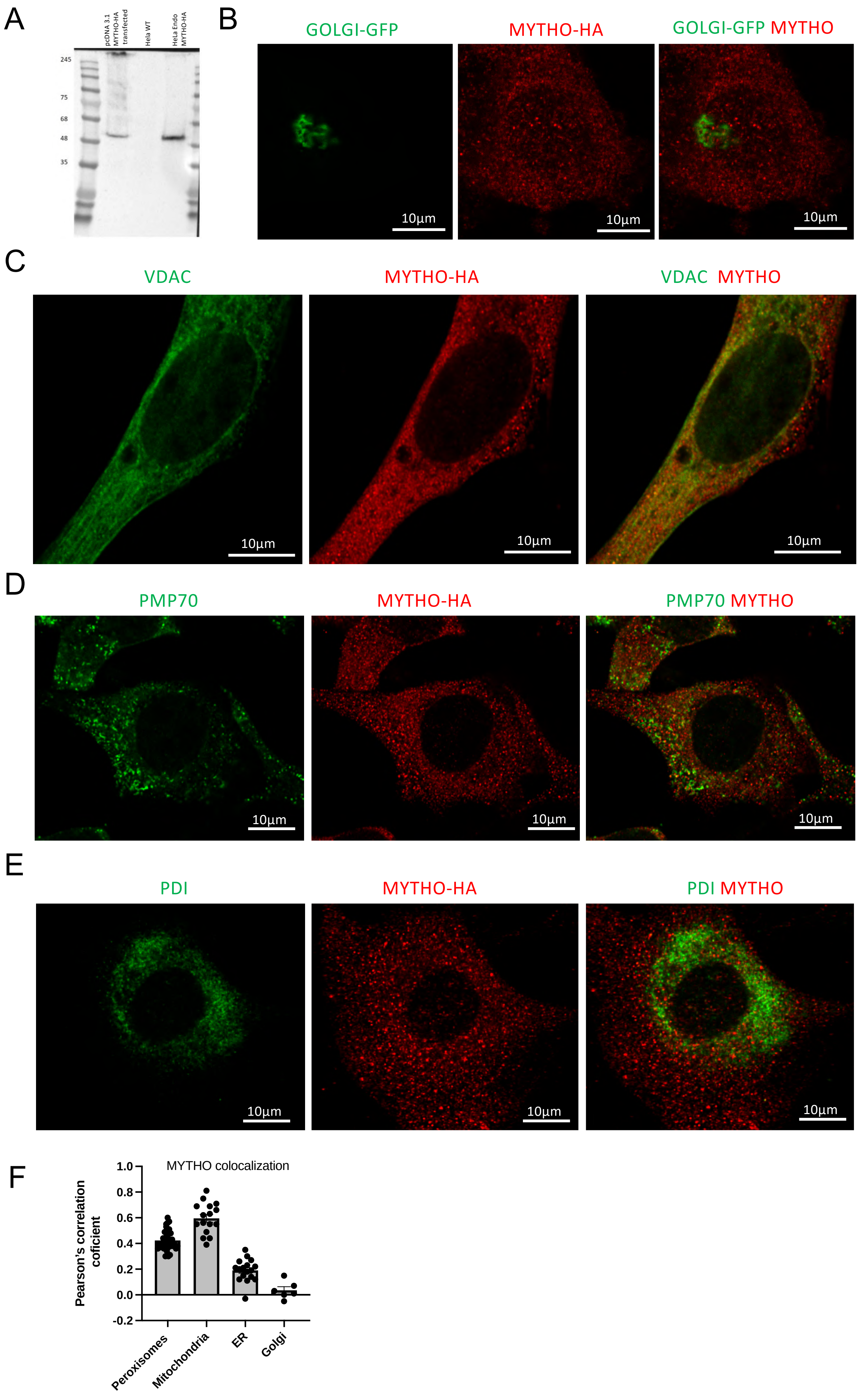

G

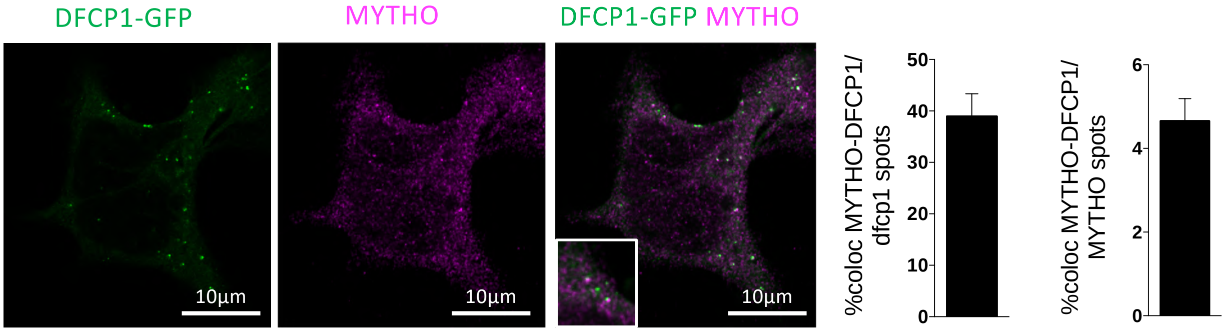

Supplemental figure 7. (A) CRISPR-CAS12 endogenous HA tagging of MYTHO. Western blot analysis using HA specific antibody in cell protein extracts from HeLa transiently transfected with MYTHO-HA pcDNA3.1 plasmid, parental HeLa and HeLa where MYTHO was HA-endogenously tagged. **(B-E)** Representative fluorescent images of MYTHO-HA HeLa cells. Scale bar=10µm. **(B)** Cells were transfected with a Golgi marker tagged with GFP and immunofluorescence was performed with an anti-HA antibody to investigate MYTHO colocalization with Golgi. **(C)** Immunofluorescence was performed against VDAC and HA to investigate MYTHO colocalization with mitochondria. **(D)** Immunofluorescence was performed against PMP70 and HA to investigate MYTHO colocalization with peroxisomes. **(E)** Immunofluorescence was performed against PDI to investigate MYTHO colocalization with ER. **(F)** Quantification of Pearson correlation coefficient between MYTHO-HA and mitochondria, peroxisomes, Endoplasmic reticulum (ER) and Golgi. (**G**) HeLa cells stably overexpressing DFCP1-GFP were fixed and the antibody against the endogenous C16orf70 was used to analyze MYTHO colocalization with DFCP1 dots in starvation. The experiment was performed three times and the colocalization quantification was done automatically using ImageJ. For each experiment 10 images at 40x magnification were analyzed. The graphs on the right represents the % of colocalized dots divided the total number of DFCP1 spots (left) or divided the total number of MYTHO spots (right). Scale bar=10µm.

Supplemental Figure 8

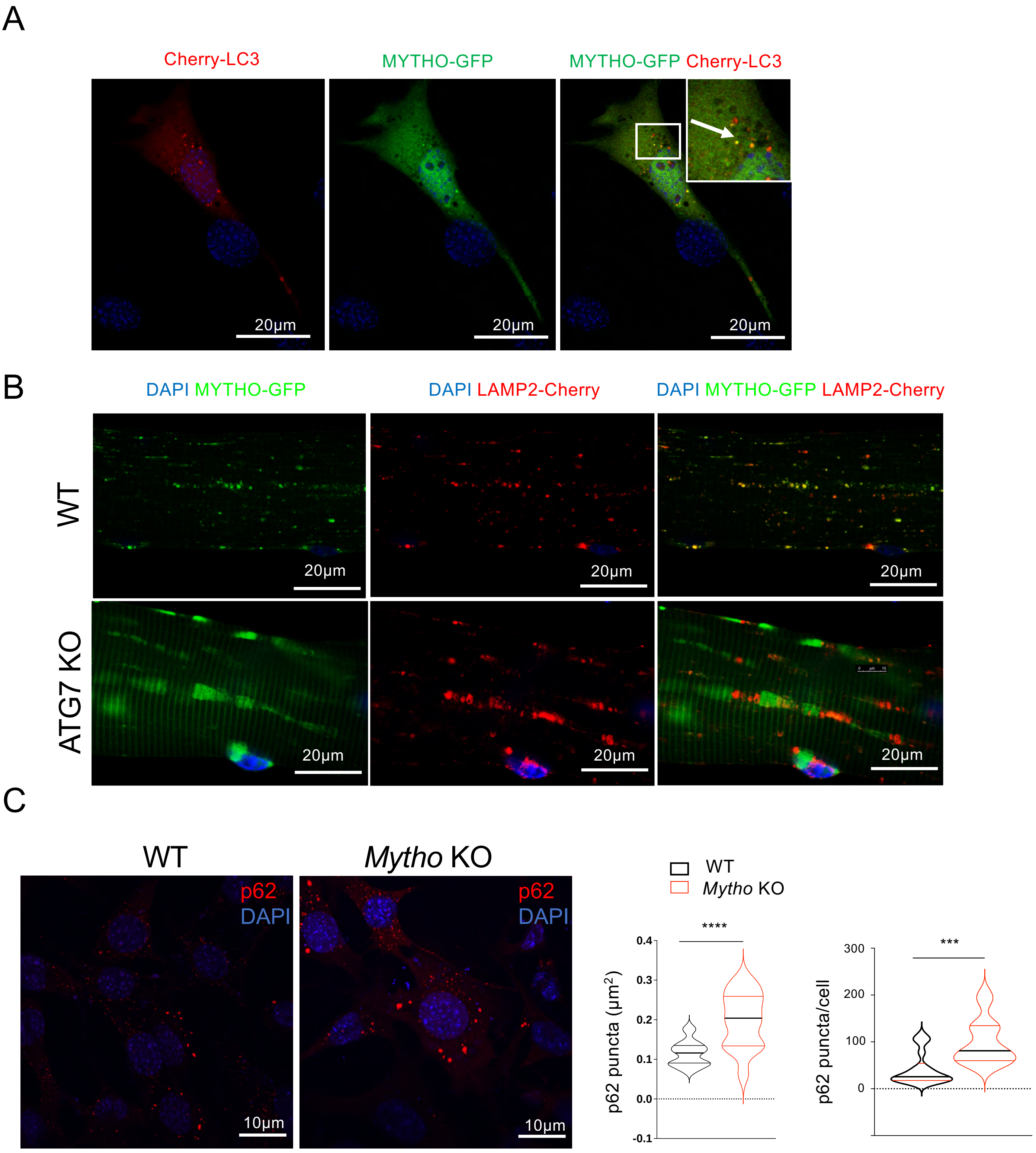

Supplemental Figure 8. (A) Representative fluorescent images of C2C12 cells co-transfected with MYTHO-GFP (green) and Cherry-LC3(red). Colocalization dots are shown in yellow. Scale bar=25µm. **(B)** Representative fluorescent images of FDB muscle fibers of *Atg7* KO or WT mice were transfected with MYTHO-GFP and LAMP-Cherry and analyzed at confocal microscope. Scale bar=20µm. (C) Representative fluorescent images of p62 puncta in WT and *Mytho* KO C2C12 (left) and quantification of the size of p62 puncta (μm^2) and the amount of puncta/cell (middle and right graphs). Scale bar=10µm. Student's t-test 2-tailed were used for statistics.

Supplemental Figure 9

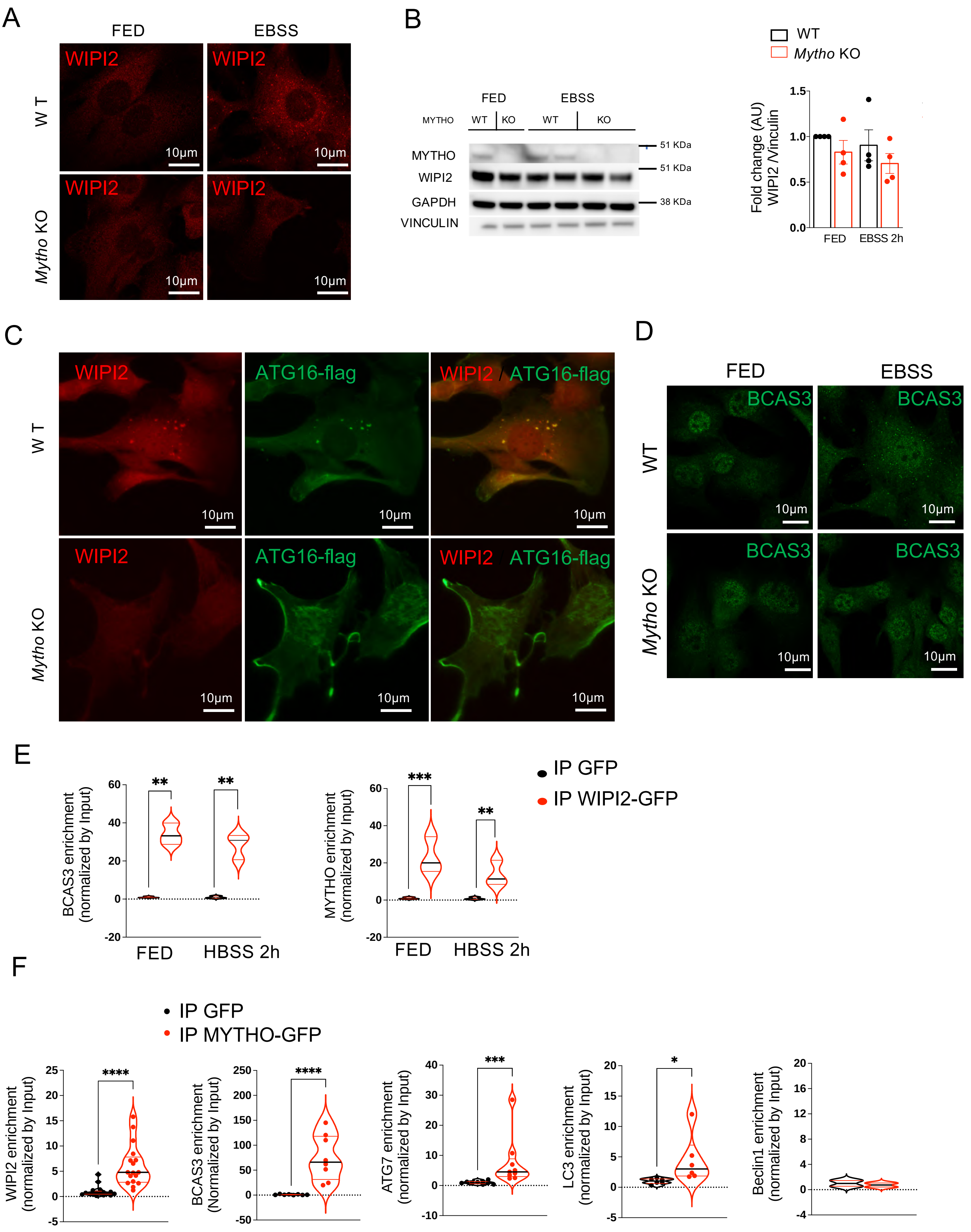

Supplemental Figure 9. (A) Representative fluorescent images of endogenous WIPI2 in WT or *Mytho* KO C2C12 cell line in FED and starved (EBSS 2 h) conditions. The quantification of three independent experiments is shown in figure 3A. Scale bar=10μm. **(B)** Left: Example of immunoblot of lysates from WT or *Mytho* KO C2C12 cell line in fed and starved (EBSS 2h) conditions. Right: Quantification of four independent experiments (the ANOVA test showed no significance between groups/conditions). **(C)** Representative fluorescent images of WT and *Mytho* KO C2C12 cell line transfected with

ATG16L1-Flag. Immunofluorescence was performed against the endogenous WIPI2 and the Flag tag. Scale bar=10μm.**(D)** Representative fluorescent images of endogenous BCAS3 in WT or *Mytho* KO C2C12 cell line in FED and starved (EBSS 2h) condition. Scale bar=10μm. **(D)** Representative fluorescent images of WT and *Mytho* KO C2C12 cell line transfected with ATG16L1-Flag. Immunofluorescence was performed against the endogenous WIPI2 and the Flag tag. Scale bar=10μm. **(E)** Quantification of MYTHO and BCAS3 endogenous proteins after immunoprecipitation of WIPI2-GFP in HEK cells in FED and HBSS (2h) conditions. Enrichment of immunoprecipitated was normalized by input and showed by fold increase. **(F)** Quantification of WIPI2, BCAS3, ATG7, LC3 and BECLIN1 endogenous proteins after immunoprecipitation of MYTHO-GFP in HEK cells in HBSS (2h) conditions. Enrichment of immunoprecipitated was normalized by input and showed by fold increase. For figure S9 E-F paired t-test 2-tailed was used for statistical analysis.

Supplemental Figure 10

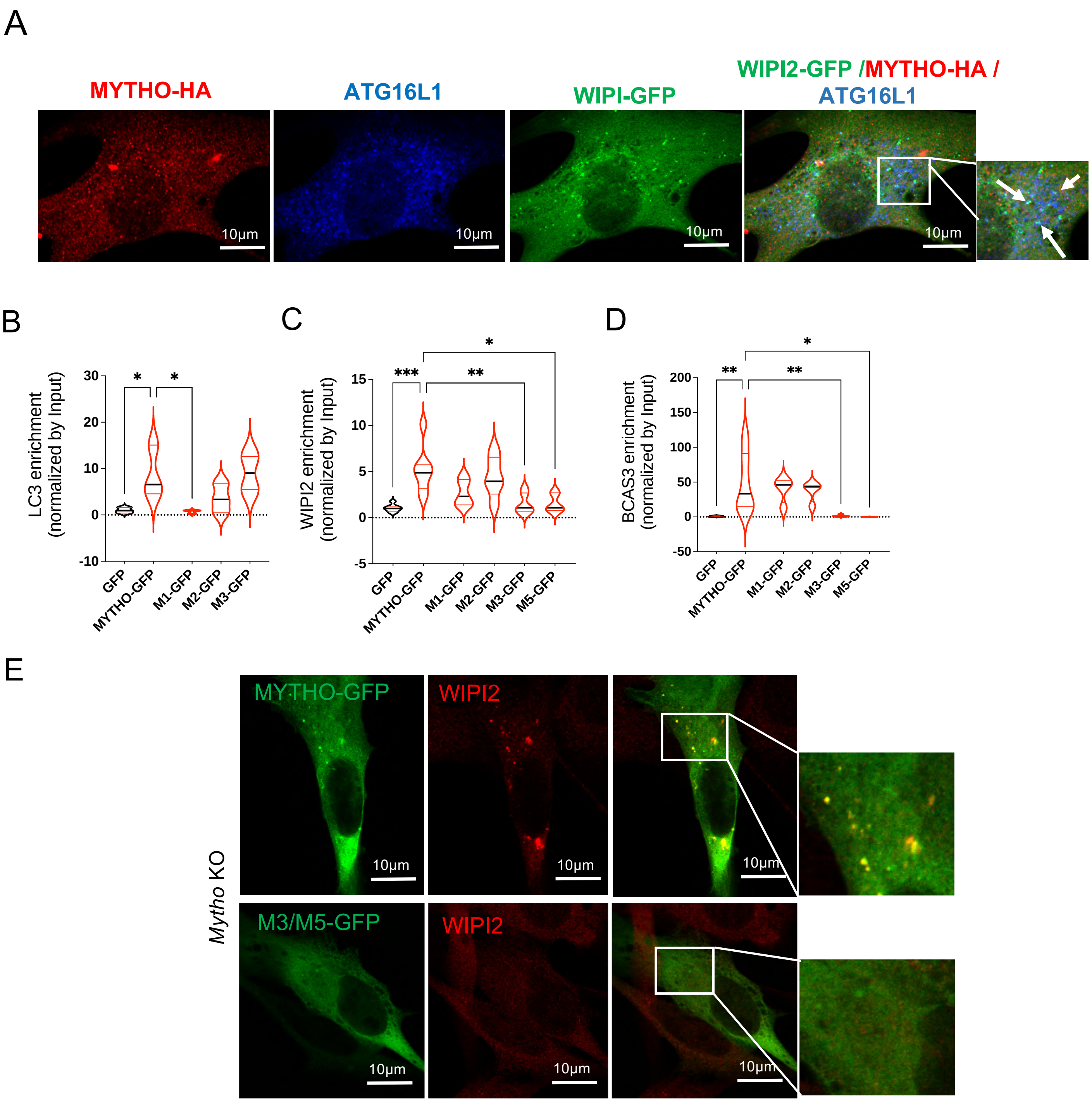

Supplemental Figure 10. (A) Representative fluorescent images of endogenous MYTHO-HA tag and ATG16L1. HEK cells were previously co-transfected with WIPI2-GFP. Arrows indicate sites of colocalization. Scale bar=10µm. **(B-D)** Quantification of LC3, WIPI2 and BCAS3 endogenous proteins after immunoprecipitation of wild-type MYTHO-GFP or mutants M1, M2, M3, M5 in HEK cells in HBSS (2 h) conditions. Enrichment of immunoprecipitated was normalized by input and showed by fold increase. 1-way ANOVA was used for statistical analysis. **(E)** Representative fluorescent images of *Mytho* KO C2C12 cells transfected with wild-type MYTHO-GFP or M3/M5 mutant. Immunofluorescence was performed against endogenous WIPI2 and the colocalization with wild-type MYTHO-GFP or M3/M5 mutants is shown in the overlay image magnification. Scale bar=10µm.

Supplemental Figure 11

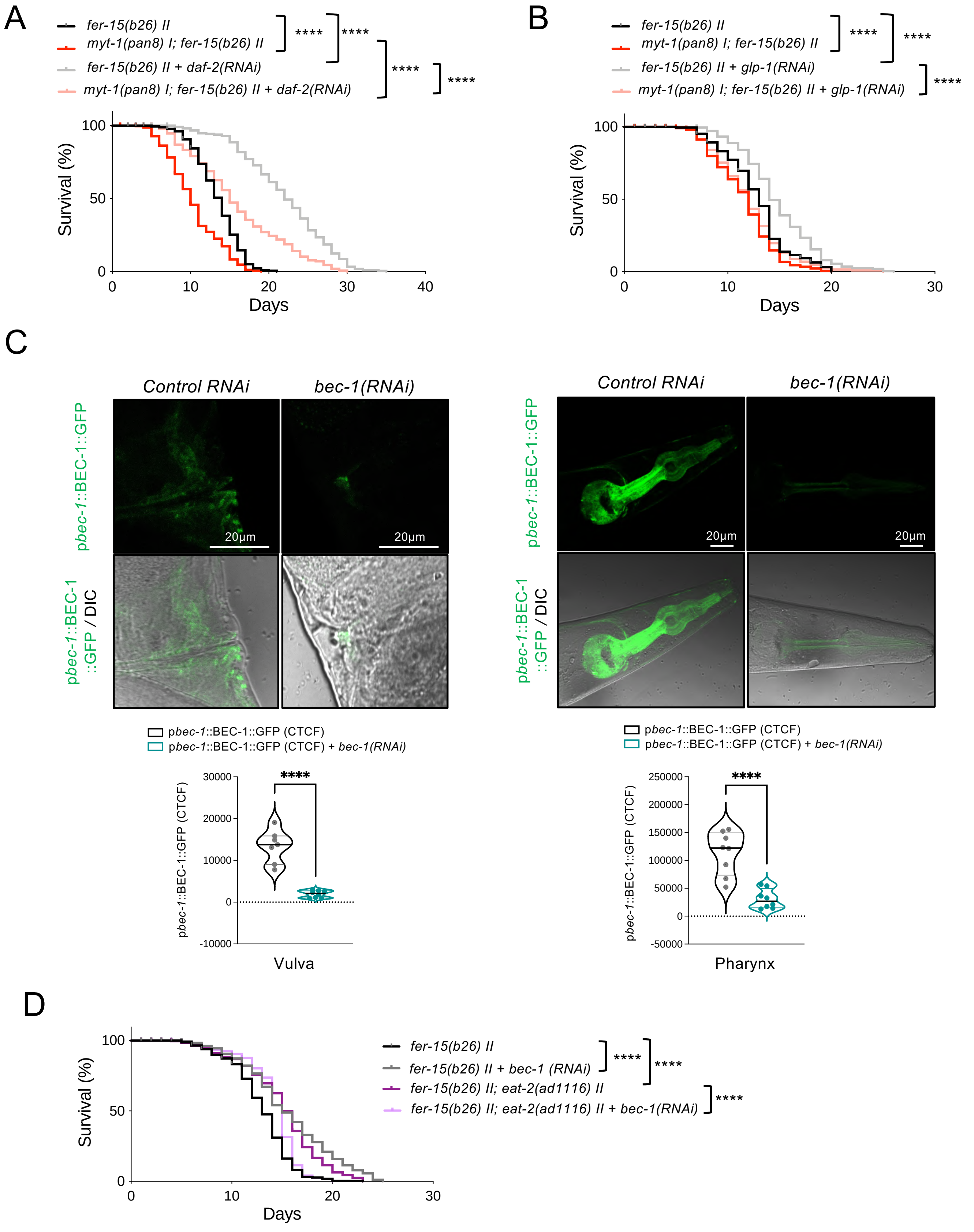

Supplemental Figure 11. (A) Survival curve of *fer-15(b26) II* (n=187), *myt-1(pan8) I; fer-15(b26) II* (n=224), *fer-15(b26) II + daf-2(RNAi)* (n=220), *myt-1(pan8) I; fer-15(b26) II + daf-2(RNAi)* (n=186) worms. **(B)** Survival curve of *fer-15(b26) II* (n=195), *myt-1(pan8) I; fer-15(b26) II* (n=223), *fer-15(b26) II+ glp-1(RNAi)* (n=223), *myt-1(pan8) I; fer-15(b26) II + glp-1(RNAi)* (n=223) worms. **(C)** Representative images of vulva (left) or pharynx (right) of transgenic FR758 (*swEx520[pbec-1::BEC-1::GFP + rol-6(su1006)]*) worms feeded with HT115(DE3) bacteria expressing control *pL4440* vector or *bec-1(RNAi)*. Corrected total cell fluorescence (CTCF) was calculated with Fiji(ImaJ) and Student's *t*-test 2-tailed was used for statistical analysis (n=7-8/condition). **(D)** Survival curve of *fer-15(b26) II* (n=252); *fer-15(b26) II + bec-1(RNAi)* (n=195); *fer-15(b26) II; eat-2(ad1116) II* (n=232) *and fer-15(b26) II; eat-2(ad1116) II + bec-1(RNAi)* (n=186) worms. For A, B and D graphs represent the composite of three independent experiments and data were analysed using the log-rank test. ****p<0.0001 (See figure S6B for lifespan experiments details and statistics).

Supplemental Methods

Muscle-specific *Atg7* KO mice
Mice bearing an *Atg7* Flox allele (Atg7 $^{f/f}$) was crossed with transgenic expressing Cre under the control of *Myosin Light Chain 1 fast* promoter (MLC1f-Atg7) as previously described(1). Mice were handled by specialized personnel under the control of inspectors of the Veterinary Service of the local Sanitary Service (ASL 16-Padova) and the local officers of the ministry of Health. Mice were housed in individual cages in an environmentally controlled room (23°C, 12h light dark cycle) with *ad libitum* access to food and water.

Mouse transfection experiments by electroporation
In vivo transfection experiments were performed in the *flexor digitorum brevis* (FDB) muscle of 3 months old C57BL/6 females (25-28 g). The animals were anesthetized by intraperitoneal injection of xylazine (Xilor) (20 mg/kg) and Zoletil (10 mg/kg). FDB muscles were injected with 10 μL of 1x hyaluronidase solution (2mg/ml) between skin and muscle using a sterile 1 ml syringe. 50 minutes later the diluted plasmidic DNA (10 μg in a maximum of 10 μL) was injected into the footpad. After 10 minutes a 27 G needle was placed through the balls of skin near the toes in a position perpendicular to the heal-toe line of the foot. The second sterile 27 G needle was placed horizontally through the skin at the heel parallel to the other 27G needle ~1 cm apart from each other. Electric pulses were applied (100 Volts/cm, 20 pulses, 20 ms intervals). FDB myofibers were first enzymatically isolated with collagenase (3mg/mL) for 1h at 37 °C, then mechanically dissociated. Isolated myofibers were plated on Matrigel overnight at 37 °C. and finally fixed with 4% paraformaldehyde in PBS for 20 minutes at room temperature in order to analyse them by confocal analysis.

Human skeletal muscle samples collection
In the present study, *Vastus lateralis* muscle biopsies from patients who underwent surgery for hip replacement were included. Patients were divided in four group of different age: 8 young (4 males and 4 females – 24-38 years old, mean age: 31.5 years ± 5.2); 7 adult (2 males and 5 females – 52-58 years old, mean age: 54.7 years ± 2.4); 7 old (3 males and 4 females – 66-75 years old, mean age: 70.0 years ± 3.8); 8 oldest-old (3 males and 5 females – 84-96 years old, mean age: 90.5 years ± 4.5). During surgery, biopsies from *Vastus lateralis* (VL) muscle were obtained and immediately snap frozen in liquid nitrogen and stored at -80 °C. Age (>20 years) and ability to provide informed consent were inclusion criteria, while exclusion criteria were chronic kidney or liver diseases, bleeding disorders, severe diabetes mellitus, rheumatic diseases other than osteoarthritis, neuromuscular disorders, malignancies and systemic infections, chronic steroid use, major psychological problems or history of alcohol or drug abuse, and evidence of prior surgery in the involved hip.

Electron microscopy analysis
5x10^{4} WT and *Mytho* KO C2C12 cells were seeded on 24-well plate in DMEM medium. 24h after cells fixation with 2.5% glutaraldehyde in buffer cacodylate, cell plate was incubated 1h at 4 ^{o}C. Then the fixative was removed and cells were conserved in fridge with cacodylate buffer until electron microscope preparation. Images were taken using the transmission Electron Microscope (TEM) FEI Tecnai G2 equipped with a side-mounted camera Olympus Veleta and a bottom-mounted camera TVIPS F114.

Mass spectrometry
All the experiments were performed in a labeling free setting. MYTHO was immunoprecipitated using HA Epitope Tag Antibody (ThermoFisher Scientific) from HeLa cells over-expressing HA tagged MYTHO. Proteins were eluted, precipitated in acetone, reduced and alkylated in a solution of 6M Guanidine-HCl, 5 mM TCEP, and 55 mM chloroacetamide. Peptides were obtained digesting proteins with LysC (FUJIFILM Wako Pure Chemical Corporation, Osaka, Japan) and Trypsin (Promega, Madison, WI, USA). Collected peptide mixtures were concentrated and desalted using the Stop and Go Extraction (STAGE) technique (2).
Instruments for LC-MS/MS analysis consisted of a Nano LC1200 coupled via a nano-electrospray ionization source to the quadrupole-based Q Exactive HF bench top mass spectrometer(3). Peptide separation was carried out according to their hydrophobicity on a PicoFrit column, 75um ID, 8Um tip, 250mm bed packed with Reprosil-PUR, C18-AQ, 1.9um particle size, 120 Ångström pore size (New Objective, Inc., cat. PF7508-250H363), using a binary buffer system consisting of solution A: 0.1% formic acid and B: 80% acetonitrile, 0.1% formic acid. Runs of 75 minutes were used for immunoprecipitated samples, with a constant flow rate of 300 nl/minute. Q Exactive HF settings: MS spectra were acquired during 3E6 as an AGC target, a maximal injection time of 20 ms and a 60,000 resolution at 200 m/z.
The mass spectrometer operated in a data dependent Top15 mode with subsequent acquisition of higher-energy collisional dissociation (HCD)fragmentation MS/MS spectra of the top 15 most intense peaks. Resolution for MS/MS spectra were set to 30,000 at 200 m/z, AGC target to 1E5, max injection time to 75 ms and the isolation window to 1.6 Th. The intensity threshold was set at 2.0e4 and Dynamic exclusion at 20 seconds.

Mass spectrometry analysis

RAW files were processed using MaxQuant (1.6.2.10) and the implemented Andromeda search engine. For protein assignment, spectra were correlated with the Uniprot Homo sapiens (v 2019) including a list of common contaminants. Searches were performed with tryptic specifications and default settings for mass tolerances for MS and MS/MS spectra. Fixed modifications Carbamidomethyl; Variable modifications: oxidation and acetylation.

Minimal peptide length was set to 7 amino acids and false discovery for proteins and peptide-spectrum matches to 1%. The Perseus software (1.6.2.3) was used and first filtered for contaminants and reverse entries as well as proteins that were only identified by a modified peptide. The LFQ Ratios were logarithmized, grouped and filtered for min. valid number (min. 3 in at least one group). Two sample *t*-test was performed using FDR=0.05. Probability values $p < 0.05$ were considered statistically significant. To identify significant enriched GO terms, we utilized the 1D enrichment tool in Perseus. Data visualization was done in the statistical environment R. MS analyses of three independent samples for each experiment were performed. Peptides with Log_2 Difference ≥ 1 and $-Log_{10}$ p-value > 1.3 were considered significantly enriched.

Paraquat analysis

The resistance to oxidative stress was tested as previously described(4) with some modifications. Briefly, L4 *fer-15(b26) II* and *myt-1(pan8) I; fer-15(b26) II* worms were seeded on NGM plates seeded with OP50 and maintained at 25 °C for 5 days. Worms were then collected in M9 buffer supplemented with paraquat at the final concentration of 125 mM and incubated at 25 °C with agitation for 1 hour. To remove the oxidant, worms were washed three times in M9 buffer and transferred to fresh NGM plates that were incubated for 48 hours at 25 °C and then survival was calculated as a ratio of worms alive to the total number of animals transferred to the plate after oxidant treatment. The experiment was performed three times.

Gene expression analysis

Total RNA was extracted from TA, Gastrocnemius muscles or from WT and Mytho KO C2C12 using TRIzol (ThermoFisher Scientific) according to the manufacturer's instructions. 400 ng of total RNA was incubated with random primer hexamers and dNTPs in a total volume of 11µL 5 minutes at 65 °C to prevent formation of RNA secondary structures. RNase Out (ThermoFisher Scientific), DTT and SuperScript IV (ThermoFisher Scientific) were added in a final volume of 20 µL. Retrotranscription reactions were performed incubating samples 23 °C 10 minutes, then 55 °C 10 minutes and finally 80 °C 10 minutes.

Quantitative real-time PCR was performed with SYBR Green chemistry (Applied Biosystems, ThermoFisher Scientific) using the following primers: murine Fw 5'-TACAGCAAGTGGGACAGCAT -3', murine Rw 5'-TGAGGCAATGTGGTTGTTCT -3', human Fw 5'- CACCATCTTCCAGGAGCGAG -3', human Rw 5'-CTTTGTGTGGAGAGCCAAGC -3'. All data were normalized to GAPDH expression. Fold change values were calculated using the Delta Delta Ct method and an unpaired t test was used to calculate statistical significance.

References:

1 Masiero E, Agatea L, Mammucari C, Blaauw B, Loro E, Komatsu M, et al. Autophagy is required to maintain muscle mass. *Cell Metab.* 2009;10(6):507-15.

2 Rappsilber J, Ishihama Y, and Mann M. Stop and go extraction tips for matrix-assisted laser desorption/ionization, nanoelectrospray, and LC/MS sample pretreatment in proteomics. *Anal Chem.* 2003;75(3):663-70.

3 Michalski A, Cox J, and Mann M. More than 100,000 detectable peptide species elute in single shotgun proteomics runs but the majority is inaccessible to data-dependent LC-MS/MS. J Proteome Res. 2011;10(4):1785-93.

4. Gusarov I, Pani B, Gautier L, Smolentseva O, Eremina S, Shamovsky I, et al. Glycogen controls Caenorhabditis elegans lifespan and resistance to oxidative stress. Nat Commun. 2017;8:15868.
