# Supplementary material for: C16ORF70/MYTHO promotes healthy aging in C.elegans and prevents cellular senescence in mammals
Source: J Clin Invest. 2024 Jun 13;134(15):e165814. doi: 10.1172/JCI165814 (PMC11291266; doi:10.1172/JCI165814)

Uncropped western blots

Full unedited gel for Figure 1A

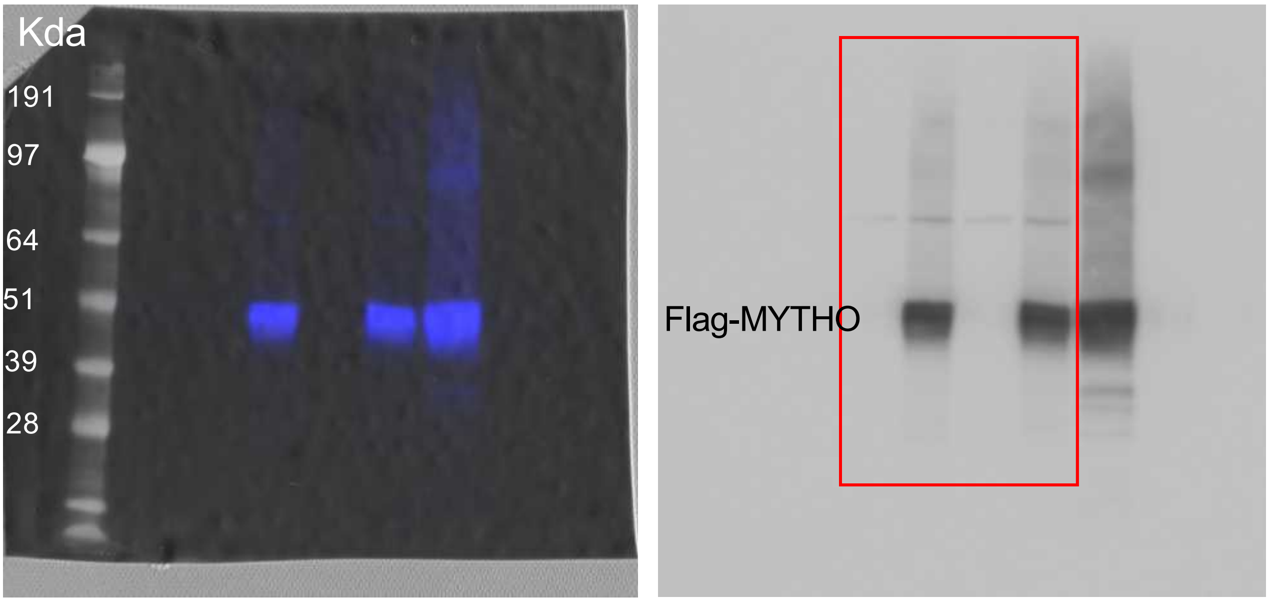

Full unedited gel for Figure 1E

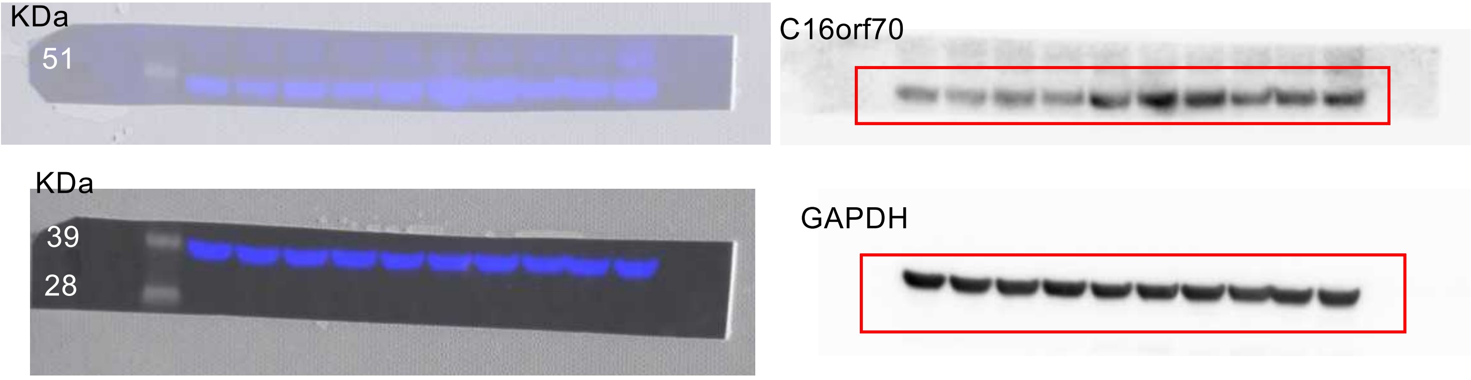

Full unedited gel for Figure 5C

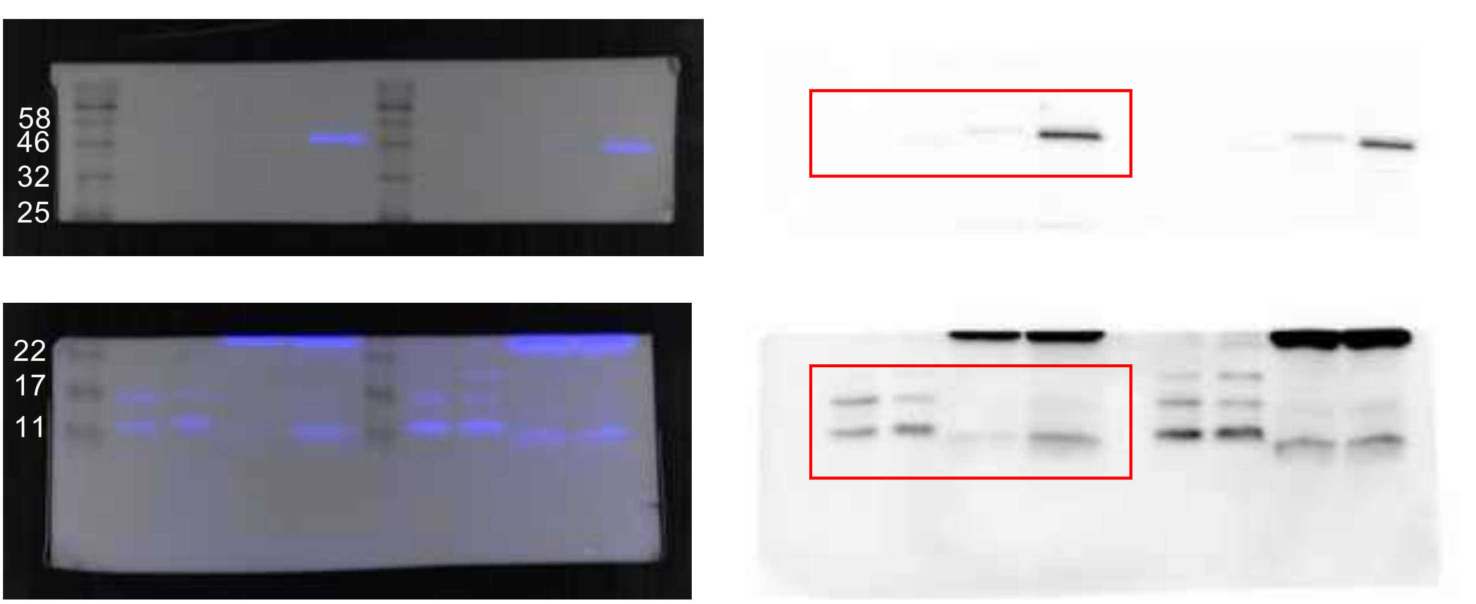

Full unedited gel for Figure 5D

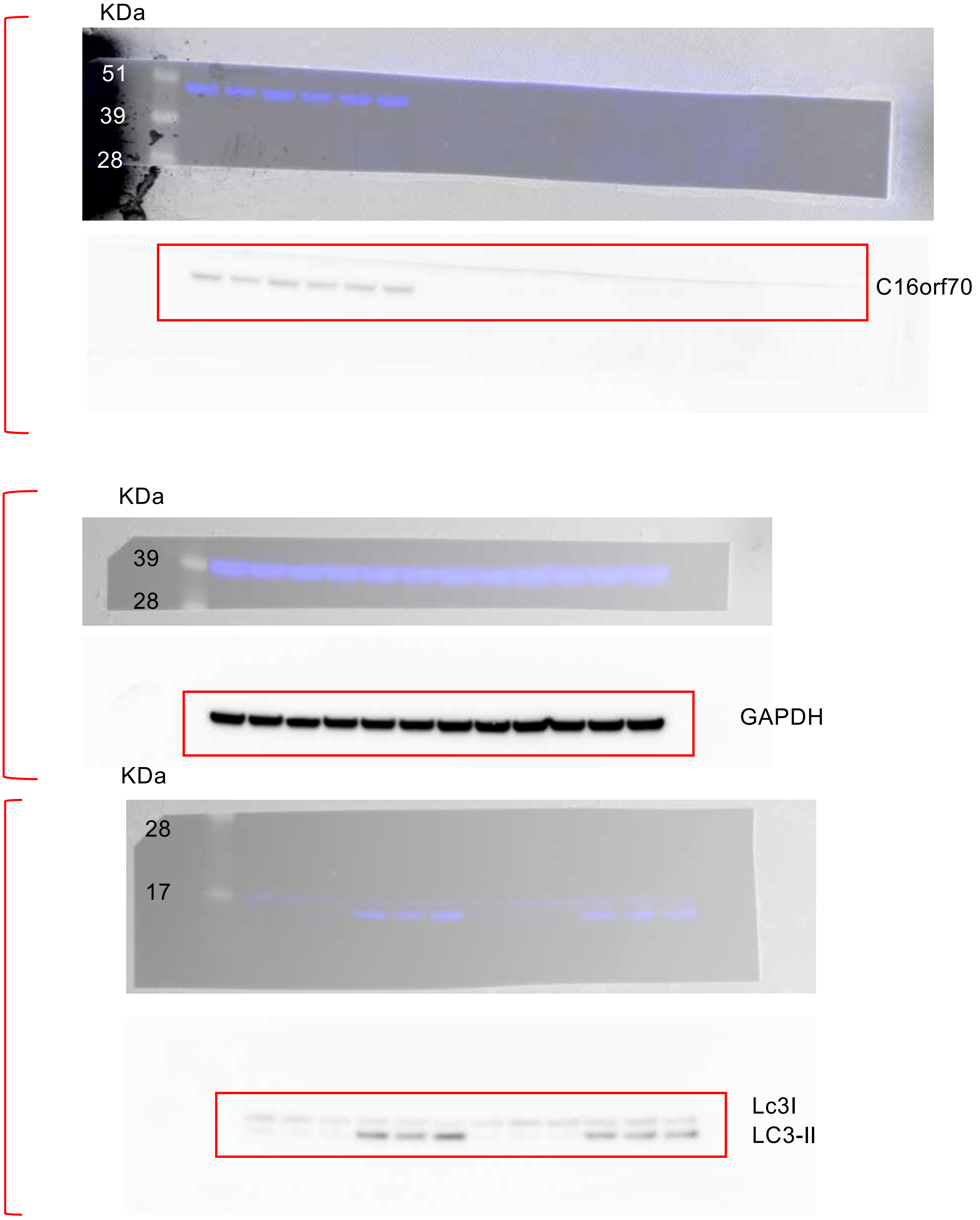

Full unedited gel for Figure 6C

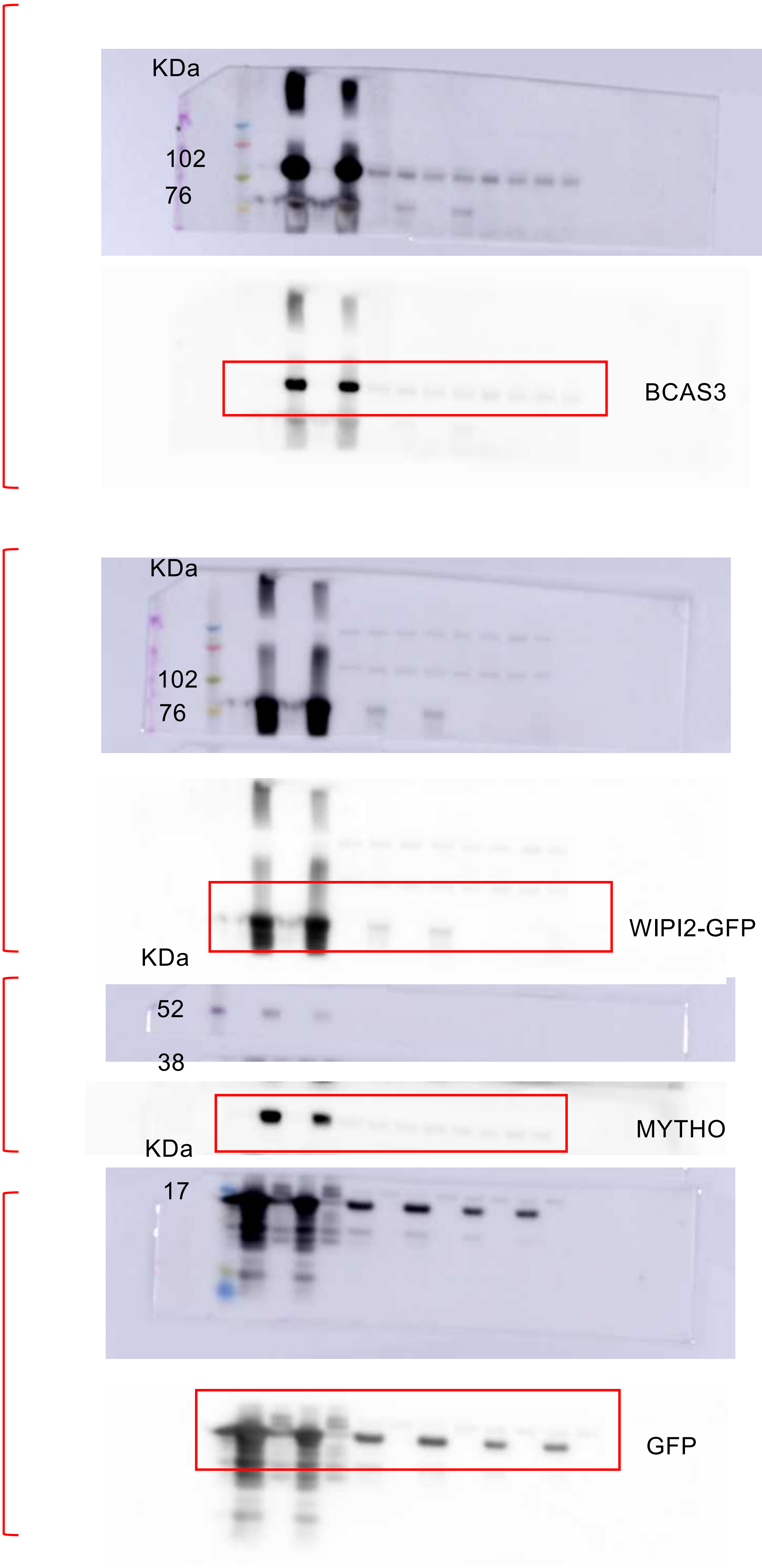

Full unedited gel for Figure 6D

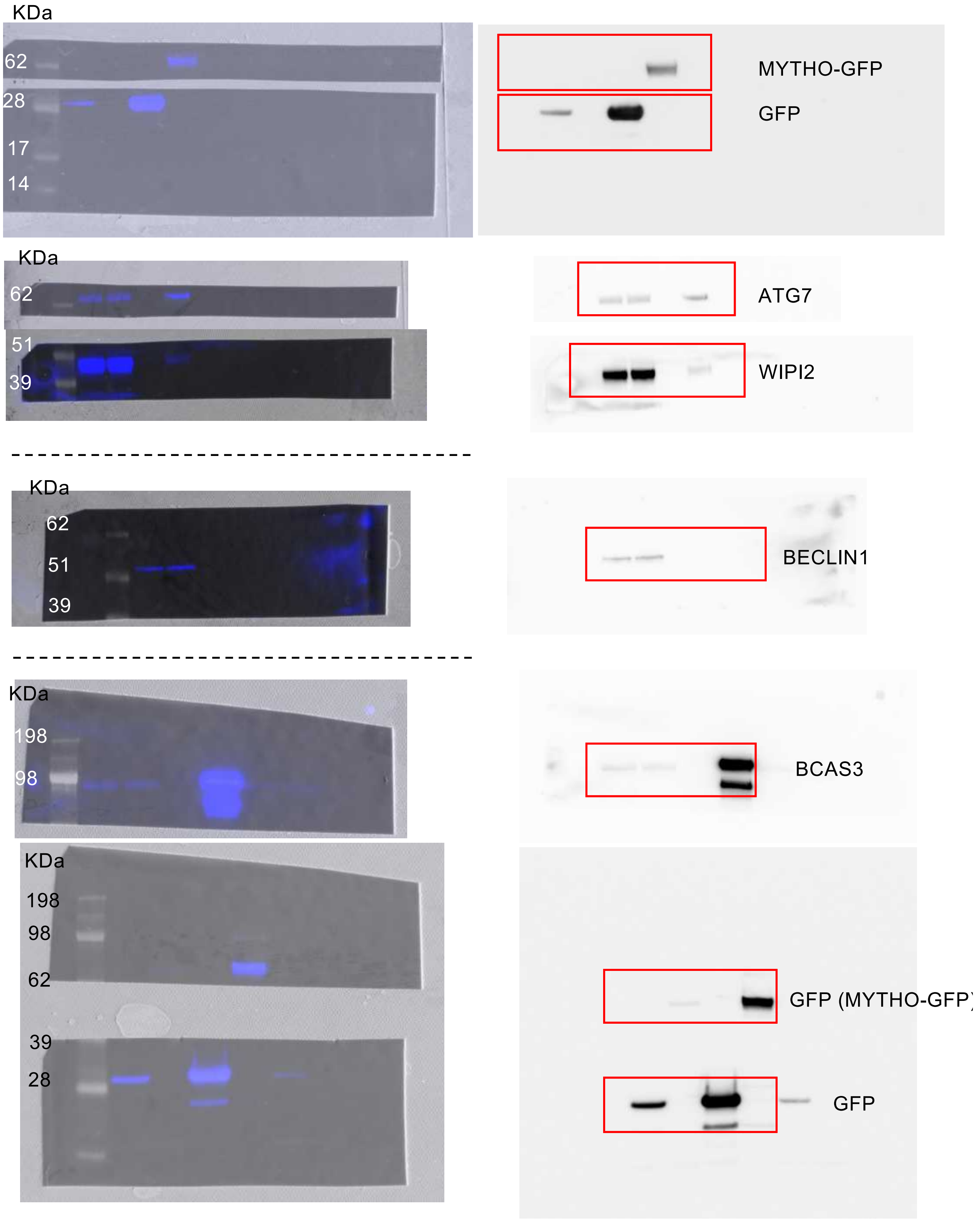

Full unedited gel for Figure 7B

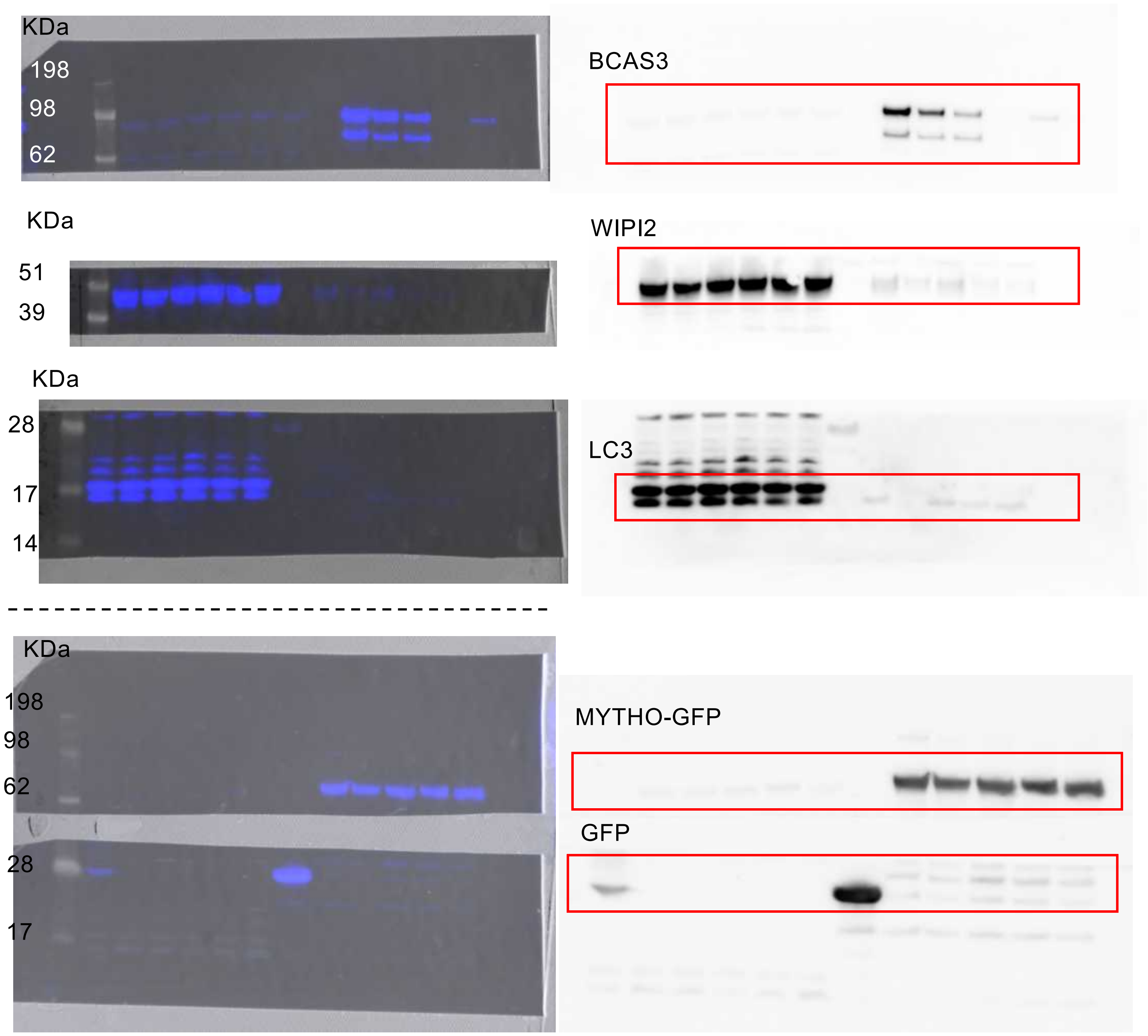

Full unedited gel for Figure 7C

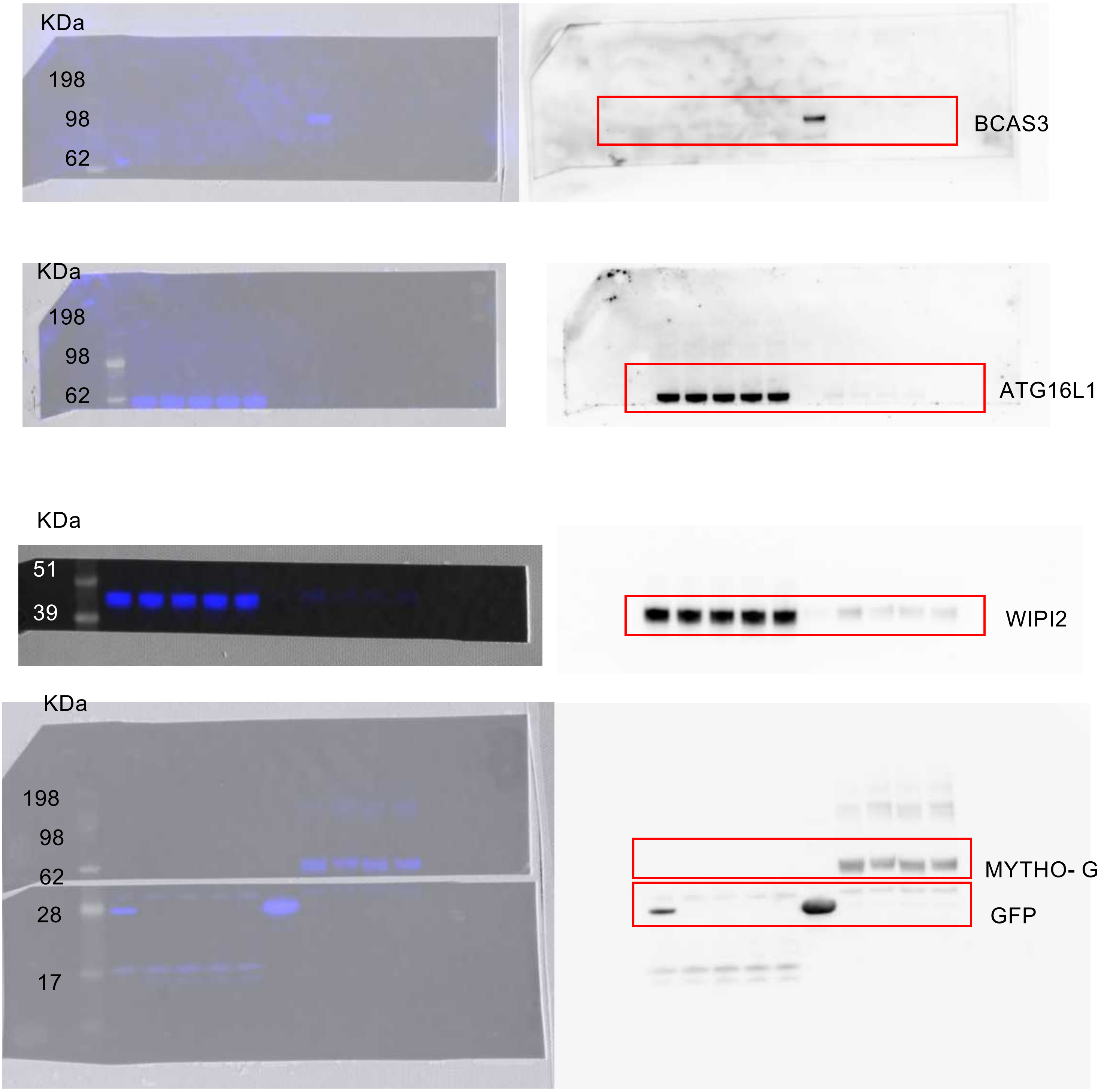

Full unedited gel for Figure 7D

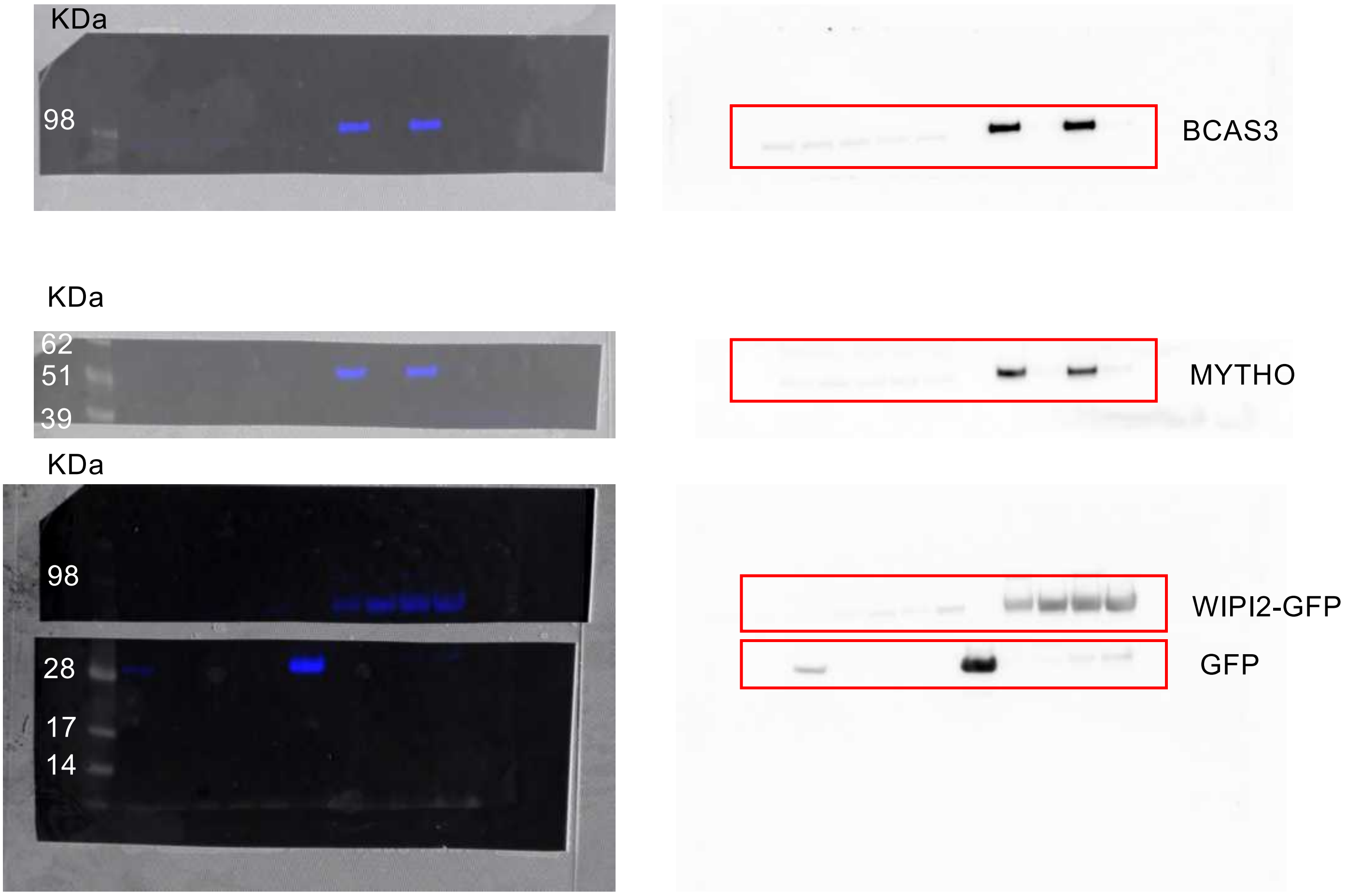

Full unedited gel for Figure S3F

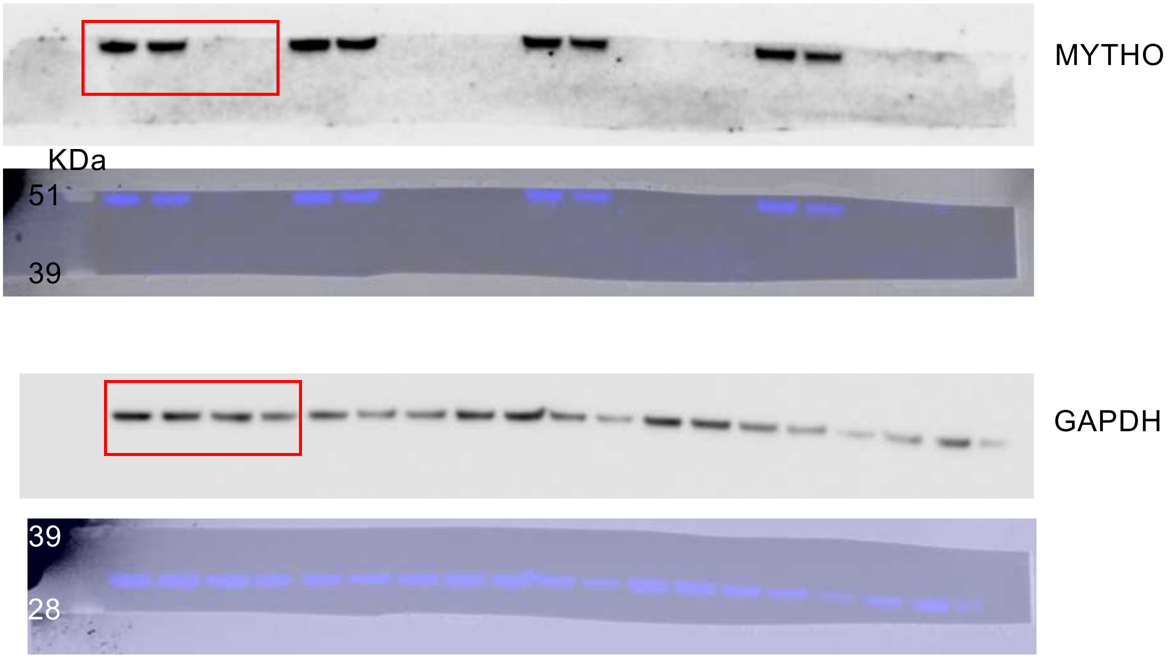

Full unedited gel for Figure S7A

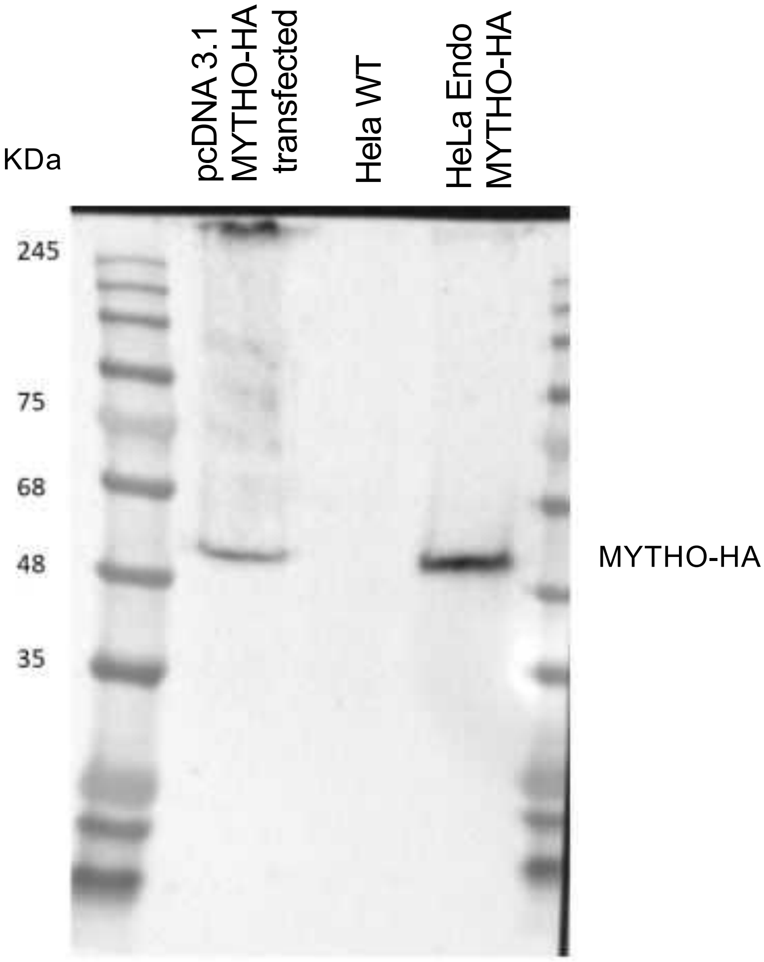

Full unedited gel for Figure S9B

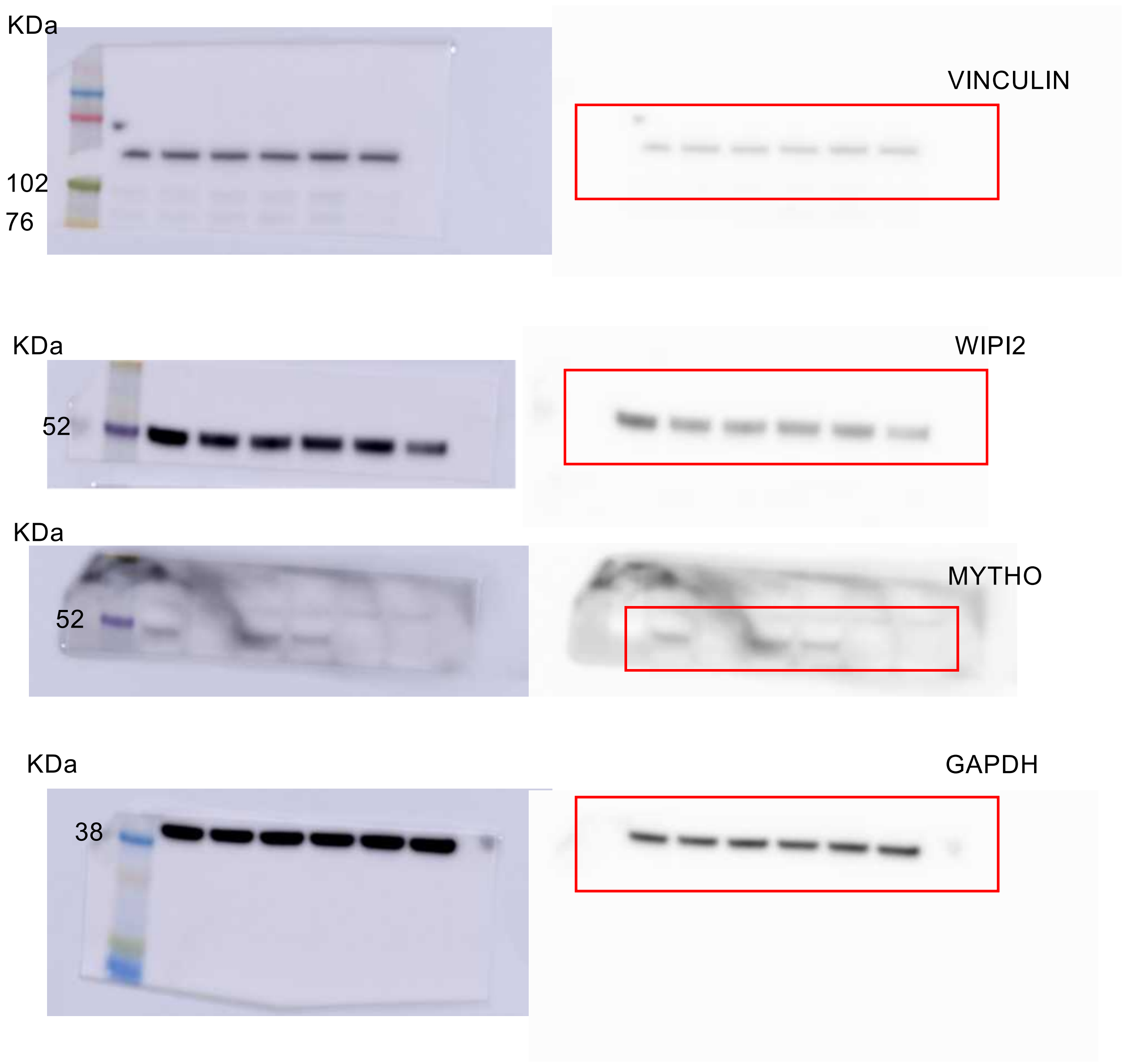

Uncropped agarose gels

Full unedited gel for Figure S4A

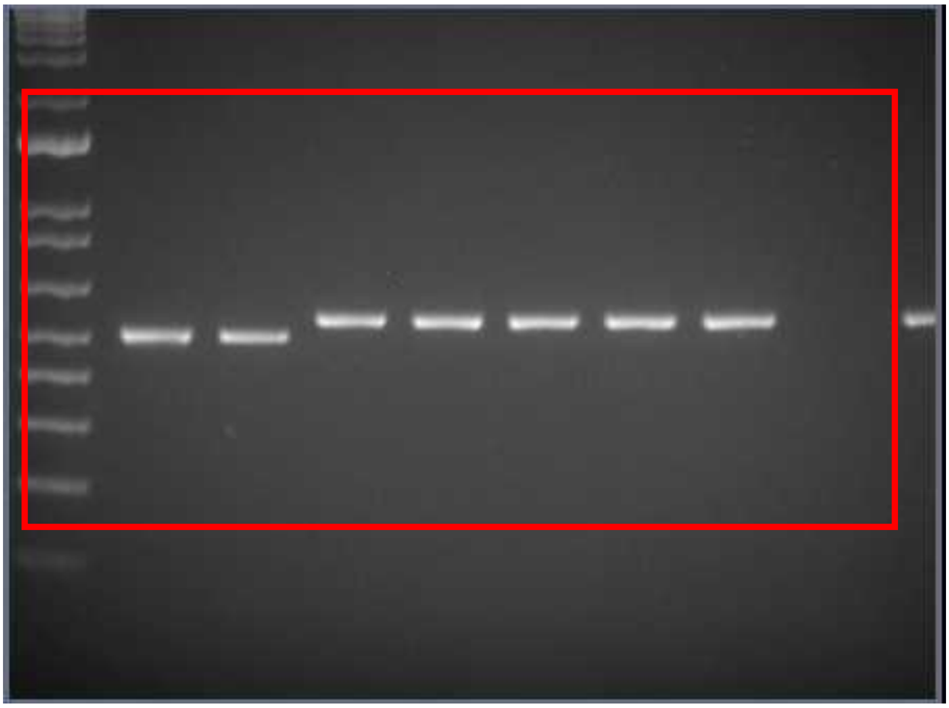

Full unedited gel for Figure S4B

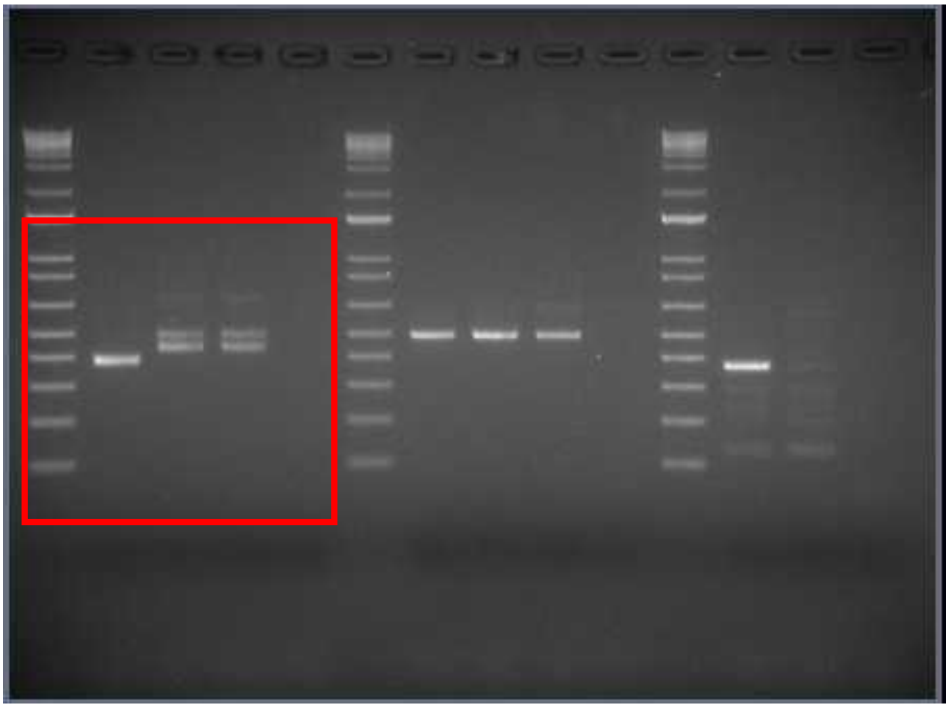

Supplement: Unedited blot and gel images [file jci-134-165814-s024.pdf]
